# Sex-specific hippocampal metabolic signatures at the onset of systemic inflammation with lipopolysaccharide in the APPswe/PS1dE9 mouse model of Alzheimer’s disease

**DOI:** 10.1016/j.bbi.2019.09.019

**Published:** 2020-01

**Authors:** Alessandra Agostini, Ding Yuchun, Bai Li, David A. Kendall, Marie-Christine Pardon

**Affiliations:** a School of Life Sciences, Division of Physiology, Pharmacology and Neuroscience, University of Nottingham, Medical School, Queens Medical Centre, Nottingham NG7 2UH, UK; b School of Computer Sciences, University of Nottingham, Jubilee Campus, Wollaton Road, Nottingham NG8 1BB, UK; c School of Computing Science, Urban Sciences Building, Newcastle University, 1 Science Square, Science Central, Newcastle upon Tyne NE4 5TG, UK^1^

**Keywords:** Inflammation, Lipopolysaccharide, Alzheimer’s disease, APP/PS1 mouse model, Sex differences, Hippocampus, Microglia, Metabolomics, Serotonin, Methionine

## Abstract

Systemic inflammation enhances the risk and progression of Alzheimer’s disease (AD). Lipopolysaccharide (LPS), a potent pro-inflammatory endotoxin produced by the gut, is found in excess levels in AD where it associates with neurological hallmarks of pathology. Sex differences in susceptibility to inflammation and AD progression have been reported, but how this impacts on LPS responses remains under investigated. We previously reported in an APP/PS1 model of AD that systemic LPS administration rapidly altered hippocampal metabolism in males. Here, we used untargeted metabolomics to comprehensively identify hippocampal metabolic processes occurring at onset of systemic inflammation with LPS (100 µg/kg, i.v.) in APP/PS1 mice, at an early pathological stage, and investigated the sexual dimorphism in this response. Four hours after LPS administration, pathways regulating energy metabolism, immune and oxidative stress responses were simultaneously recruited in the hippocampi of 4.5-month-old mice with a more protective response in females despite their pro-inflammatory and pro-oxidant metabolic signature in the absence of immune stimulation. LPS induced comparable behavioural sickness responses in male and female wild-type and APP/PS1 mice and comparable activation of both the serotonin and nicotinamide pathways of tryptophan metabolism in their hippocampi. Elevations in N-methyl-2-pyridone-5-carboxamide, a major toxic metabolite of nicotinamide, correlated with behavioural sickness regardless of sex, as well as with the LPS-induced hypothermia seen in males. Males also exhibited a pro-inflammatory-like downregulation of pyruvate metabolism, exacerbated in APP/PS1 males, and methionine metabolism whereas females showed a greater cytokine response and anti-inflammatory-like downregulation of hippocampal methylglyoxal and methionine metabolism. Metabolic changes were not associated with morphological markers of immune cell activation suggesting that they constitute an early event in the development of LPS-induced neuroinflammation and AD exacerbation. These data suggest that the female hippocampus is more tolerant to acute systemic inflammation.

## Highlights

• Hippocampal metabolic profile of females is more pro-inflammatory and pro-oxidant.
• Comparable LPS-induced sickness behaviour in male and female WT and APP/PS1 mice.
• Pro- and anti-inflammatory pathways both recruited 4 h after systemic LPS.
• Predominant anti-inflammatory metabolic response to LPS in female hippocampi.

## 1 Introduction

Alzheimer’s disease (AD), the most common senile dementia, is characterised by a progressive cognitive decline accompanied by the accumulation of aggregated amyloid beta (Aβ) plaques, neurofibrillary tangles made of hyperphosphorylated tau protein, severe brain atrophy and neuroinflammation. The causes of AD are far from being understood, but systemic infection and inflammation have emerged as key modulators of its risk and progression. A number of genes conferring susceptibility to inflammatory conditions have indeed been found to be associated with a predisposition to AD (Karch and Goate, 2015, Malik et al., 2015, Yokoyama et al., 2016), whereas circulating levels of acute phase proteins or pro-inflammatory cytokines were found to be elevated in non-demented subjects presenting with a higher risk of developing late-onset AD (Eikelenboom et al., 2012, Koyama et al., 2013), and in patients in the prodromal, mild cognitive impairment (MCI) phase of AD (Bettcher and Kramer, 2014, King et al., 2018, Trollor et al., 2010). Infection-induced systemic inflammation has been proposed as a mechanistic driver of AD pathogenesis (Ashraf et al., 2019, Giridharan et al., 2019), and the presence of acute inflammatory events, such as respiratory infections or delirium have also been associated with exacerbations of clinical presentation and precipitous cognitive decline in AD patients (Holmer et al., 2018, Holmes et al., 2009, Ide et al., 2016). Altogether, this suggests that AD patients and people at risk of developing the disease are more susceptible to inflammatory conditions, and that such vulnerability contributes to the development of clinical features of AD. The incidence and prevalence of AD are generally higher in women, and although this may be due to their longer life expectancy, they exhibit faster cognitive decline and brain atrophy than men (Ferretti et al., 2018, Podcasy and Epperson, 2016) and are also thought to produce higher inflammatory responses and be more susceptible to inflammatory conditions (Klein and Flanagan, 2016, Roved et al., 2017). Some sex differences in the association between specific pro-inflammatory mediators and clinical outcomes have been noted (Trollor et al., 2010), but this has not been investigated in detail.

Systemic inflammation is thought to be the mechanism whereby acute, accumulative or chronic infections can trigger AD pathogenesis (Ashraf et al., 2019, Giridharan et al., 2019). In preclinical mouse models, lipopolysaccharide (LPS), mimicking gram-negative bacterial infection, and other acute systemic inflammatory stimuli have been found to exacerbate cognitive dysfunction, Aβ plaque load and tau phosphorylation (Barron et al., 2017, Cunningham and Hennessy, 2015, Nazem et al., 2015). While the use of LPS to model systemic inflammation has been debated, in part because of the high doses used which are more relevant to sepsis than to the chronic low grade inflammation associated with ageing, MCI and AD (Barron et al., 2017, Cunningham and Hennessy, 2015, Varatharaj and Galea, 2017), a comparison of three models yielded the conclusion that LPS is a suitable model for studying the impact of new therapies for acute systemic inflammation (Seemann et al., 2017). But importantly, this endotoxin is produced by the gut microbiota in response to systemic infections, and its subsequent release in the systemic circulation plays a key role in the development and persistence of systemic inflammation (Maldonado et al., 2016, Thorburn et al., 2018). Circulating LPS levels are elevated in AD patients (Zhang et al., 2009) and the recent discoveries of LPS infiltration in the post-mortem AD brain where it associates with Aβ plaques, highlights the clinical relevance of this immune model (Zhan et al., 2018, Zhan et al., 2016, Zhao et al., 2017). This has led to the proposal that endogenous LPS accumulation could play a critical role in the pathophysiology of the common, sporadic form of AD (Pistollato et al., 2016, Sochocka et al., 2019, Zhan et al., 2018). To the best of our knowledge, endogenous LPS levels have not been quantified in AD models. Differences in gut microbiota composition between genetic models of AD and their wild type control, consistent with endotoxemia and susceptibility to LPS, have been reported and found associated with the progression of cerebral amyloidosis (Brandscheid et al., 2017, Harach et al., 2017, Zhang et al., 2017). Removal of microbiota from a humanized AD model delayed substantially Aβ plaque deposition, while colonisation of these mice with gut microbiota from a conventional AD model, but not from their wild type control, accelerated Aβ deposition (Harach et al., 2017). There is, therefore, a need to better understand the mechanisms whereby systemic LPS affects the brain and contributes to AD progression.

LPS, is an agonist of the toll-like receptor 4, which in the brain, is almost exclusively expressed by microglia (Hanke and Kielian, 2011), the resident immune cell in the central nervous system. Microglia play a critical role in the clearance of Aβ and tau aggregates, and their dysfunction is associated with the genetic risk of developing AD (Hansen et al., 2018, Perea et al., 2018). At low doses able to induce physiologically relevant low grade inflammation, penetration of LPS in the mouse brain is limited in the absence of blood brain barrier dysfunction (Banks and Robinson, 2010, Varatharaj and Galea, 2017). However, pro-inflammatory changes in microglia can be seen as early as 4 h post-inoculation depending on the disease status (Murray et al., 2011, Pardon et al., 2016). Using magnetic resonance spectroscopy, we previously observed that mild systemic inflammation, induced with the low 100 µg/kg dose of LPS, rapidly altered hippocampal metabolism in the APPswe/PS1dE9 (APP/PS1) mouse model of amyloidosis and its wild-type (WT) littermates at early to advanced pathological stages (Pardon et al., 2016). The metabolic changes occurring within 4 h of immune stimulation also discriminated the microglial response of WT and APP/PS1 mice (Pardon et al., 2016). Variations in brain metabolism and substrate availability are thought to influence microglial function, although the mechanisms involved are not clear (Ghosh et al., 2018). In the same APP/PS1 mouse model of amyloidosis used in our previous study, age- and region-specific metabolic perturbations have been reported in the brain of males and females, but sex differences have not been systematically tested (Gonzalez-Dominguez et al., 2014, Maroof et al., 2014), although they have been seen with brain aging in WT mice and are thought to contribute to differential susceptibility to AD-like pathology (Zhao et al., 2016). Preclinical data from genetically altered mouse models of AD indeed confirm that cerebral amyloidosis develops faster in females than in males (Li et al., 2016, Wang et al., 2003). Thus, metabolic responses to systemic inflammation could mediate exacerbation of AD-like pathology and the impact of sex on disease progression.

In this context, we aimed, in the present study, to gain further understanding of the metabolic processes occurring at onset of systemic inflammation with LPS, and used untargeted metabolomics to comprehensively identify pathways that rapidly respond to immune stimulation in WT and APP/PS1 mice of both sexes. We tested the hypothesis that APP/PS1 mice would be more susceptible to the metabolic effects of LPS, and postulated a sexual dimorphism in the hippocampal metabolic response to systemic inflammation. As reviewed above, systemic inflammation is expected to be an early event in the pathogenesis of AD; we therefore used 4.5-month-old mice, an age characterised by the appearance of the first plaques and subtle cognitive deficits (Bonardi et al., 2011, Malm et al., 2011, Maroof et al., 2014). Our results indicate that pathways regulating energy metabolism, immune and oxidative stress responses are simultaneously recruited 4 h after systemic LPS, and comparably in the hippocampus of both WT and APP/PS1 mice, whose hippocampal metabolism was similar in the absence of immune stimulation. While unchallenged females exhibited a pro-inflammatory and pro-oxidant hippocampal metabolic signature compared to males, the recruitment of some pathways at onset of systemic inflammation was sex-dependent with the metabolic response of females shifting towards a more pronounced anti-inflammatory and neuroprotective component than males, which also showed more severe sickness symptoms at this time point.

## 2 Material and methods

### 2.1 Ethics statement

All procedures were carried out in accordance with the UK Animals (Scientific Procedures) Act of 1986 under project license 40/3601, approved by the University of Nottingham Ethical Review Committee and are reported according to the ARRIVE guidelines (Kilkenny et al., 2010). All analyses were performed in blind.

### 2.2 Animals

Forty-four 4.5-month-old male and female APPswe/PS1dE9 (APP/PS1, (Jankowsky et al., 2004)) mice and their wild-type (WT) littermates were used (n = 5–6 per sex, genotype and treatment). All experimental animals were bred and maintained in the University of Nottingham Biomedical Service Unit as previously described (Pardon et al., 2016). Genotyping was performed by Transnetyx (Cordova, TN, USA). Mice were maintained group-housed in individually vented cages (3–4 per cage) under standard husbandry conditions with *ad libitum* access to food and water, and were provided with nesting material and a play tube. The room was on a 12/12 h light cycle with lights on at 07:00 h; temperature, relative humidity and air exchange were automatically controlled.

### 2.3 Drug treatment

Lipopolysaccharide (LPS, Escherichia coli serotype Sigma0111:B4, Sigma Aldrich) was dissolved in phosphate buffered saline (PBS, Sigma Aldrich) at a concentration of 200 μg/ml, and stored in aliquots at −20 °C until use. On the day of the experiment, LPS was further diluted 1:2 in PBS to a final concentration of 100 μg/ml. Mice were injected intravenously (i.v.) in the lateral tail vein with 100 μg/kg of LPS, or an equivalent volume of its vehicle PBS, as previously described (Pardon et al., 2016).

### 2.4 Study design

**Fig. 1:**
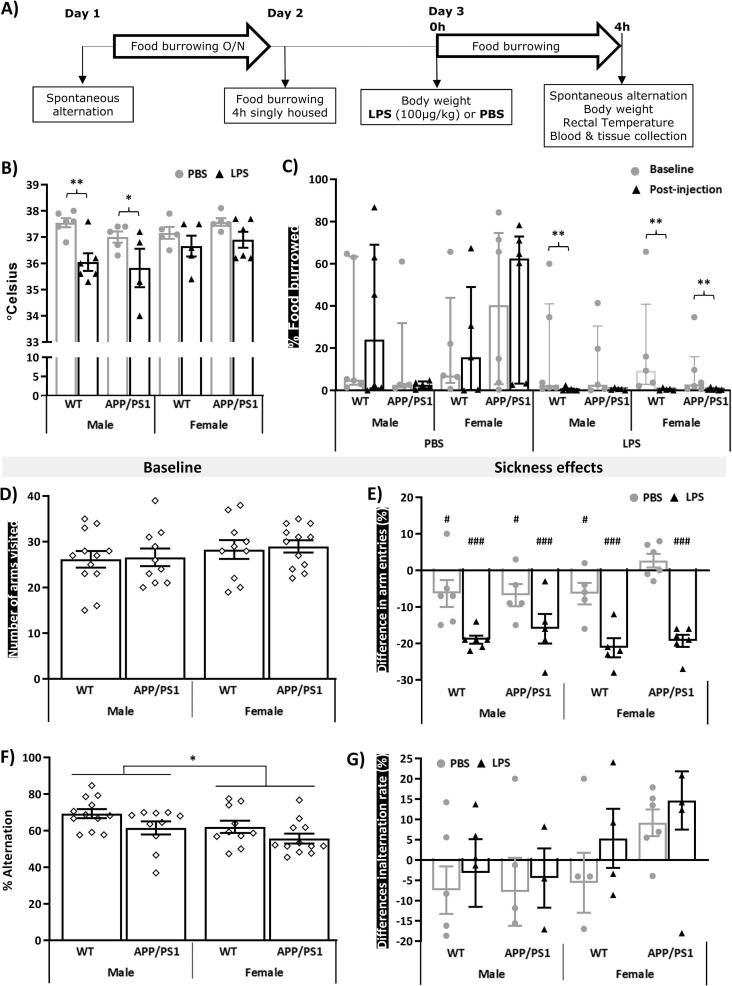
LPS-induced behavioural suppression at 4 h post-injection is independent of sex or genotype. A) Timeline of the experiment. 4.5-month-old male and female APP/PS1 mice and their wild-type (WT) littermates (n = 5–6) were subjected to baseline assessment of spatial working memory performance and exploratory drive in the spontaneous test as well as food burrowing behaviour prior to receiving a tail vein injection of lipopolysaccharide (LPS, 100 μg/kg) or its vehicle (phosphate buffer saline, PBS). Induced sickness effects were tested at 4 h post-injection in the same tests, prior to blood and tissue collection. At this time point, a significant decrease in core body temperature was observed in males, regardless of their genotype (B). LPS also suppressed food burrowing activity (C) and exploratory drive in the spontaneous alternation test, assessed through the number of arms visits (E), regardless of sex and genotype, but baseline performance for these behavioural measures did not differ between groups (C, D). Female mice overall exhibited lower spontaneous alternation performance than their male counterparts at baseline (F), but LPS had no significant impact on this measure (G). Parametric data are expressed as Means ± SEM. Dots represent individual animals. Post-hoc tests: *p < 0.05; **p < 0.01, ***p < 0.0001 *vs* PBS or baseline. Food burrowing data were rank-transformed for statistical analysis but represented as non-normalised responses and expressed as Median ± interquartile range. Sickness scores are represented as the difference between pre- and post-injection performance. Within-subjects pairwise comparisons following 3-way ANOVAs: ^#^p < 0.05; ^##^p < 0.01, ^###^p < 0.0001 compared to baseline performance (E).

The timeline of the experiment is represented in Fig. 1A. 4.5-month-old male and female APP/PS1 and WT mice were randomly allocated to the LPS or PBS treatment groups (n = 5–6). Baseline behavioural assessment was carried out on days 1 & 2. Mice were first tested for spatial working memory performance and exploratory drive in the spontaneous alternation test (Day 1). They were then trained to burrow food in groups overnight in their home cage (Deacon, 2012) and on Day 2, underwent baseline food burrowing testing over 4 h while singly housed. On Day 3, mice were challenged with LPS (100 μg/kg i.v.) or PBS (1 μl/g of body weight). Post-treatment sickness effects were assessed 4 h after injection in the food burrowing and spontaneous alternation tests, by monitoring changes in body weight and assessing body temperature taken using a rectal probe at the time of culling. Immediately after the spontaneous alternation task, mice were culled by cervical dislocation and trunk blood was collected. Their brains were removed; the hippocampi were dissected from one hemisphere, snap frozen and stored at −80 °C until use for metabolomics. The second hemisphere was post-fixed by immersion in 4% paraformaldehyde, stored at 4–8 °C for a minimum of 24  h, and then embedded in paraffin wax on a tissue embedding station (Leica TP1020).

### 2.5 Behavioural assessment

#### 2.5.1 Food burrowing

Food burrowing is a species-specific behaviour, largely dependent of the integrity of the hippocampus (Deacon et al., 2002), which is suppressed in response to systemic inflammation (Teeling et al., 2007). The protocol was adapted from one previously described (Geiszler et al., 2016). A glass jar containing 30 g of food pellets broken into small pieces was added to the home cage overnight for training in groups, or in individual cages for the two test sessions, with *ad libitum* access to food and water. The amount of food displaced from the jar was recorded, expressed as a percentage from the 30 g provided, and used as a measure of food burrowing performance. To assess sickness effects, the difference between pre- and post-injection burrowing performance was calculated.

#### 2.5.2 Spontaneous alternation

Spontaneous alternation was used as previously described (Geiszler et al., 2016, Maroof et al., 2014) to assess spatial working memory and exploratory drive. The latter is suppressed in response to LPS-induced sickness and is a potential confounding factor for the assessment of cognitive effects (Cunningham and Sanderson, 2008). The Y-shaped maze comprised three identical transparent Plexiglas® arms at a 120° angle from each other (41.5 cm in length and 6 cm in width surrounded by 15 cm high transparent Perspex walls). The start point (6 cm × 7.5 cm) was located in the center of the maze, and the mice were allowed to freely explore the three arms over five minutes. The number of alternations was recorded manually and expressed as a percentage of alternations to estimate spatial working memory performance, while the number of arms visited was used as an indication of exploratory drive. To assess sickness effects, the difference between pre- and post-injection performance was calculated. Mice that entered only one arm after the LPS challenge (1 wt female, 1 wt male and 2 APP/PS1 males) were excluded from sickness data as their alternation rate post-injection could not be calculated, but remained included in the analysis of baseline performance.

### 2.6 Immunohistochemical analyses

#### 2.6.1 Immunohistochemistry

7 μm-thick coronal sections were cut throughout the hippocampus using a microtome (Microtome Slee Cut 4060), mounted on APES-coated slides and dried overnight at 40 °C. Immunostaining of the microglial marker Ionized calcium binding adaptor molecule 1 (Iba1) and the astrocyte marker glial fibrillary acidic protein (GFAP) was carried out using standard protocols as previously described (Pardon et al., 2016), in 6–8 brain slices per brain. Incubation with rabbit anti-Iba1 [Wako, cat. nr. 019–19741; 1:6000 in PBS-Tween (0.05% Tween-20 in PBS)] or anti-GFAP (Biogenix, cat. nr. AM020-5 M, 1:4000 in PBS-T) antibodies was carried out for 1 h at room temperature. Biotinylated secondary antibody (Vectastain Elite ABC Kit, Rabbit IgG, Vector Labs, Burlingame, CA cat. nr. PK-6101, 1:200 in PBS-T) was applied for 30 min. Tissue was washed, exposed to ABC-HRP (Vectastain Elite ABC Kit R.T.U, Vector Labs, cat. nr. PK-7100), labelled with DAB peroxidase substrate (Vector Labs cat. SK-4100) according to manufacturer’s instructions, and counterstained using a haematoxylin and eosin protocol. Digital focused photo-scanning images were then acquired using a Hamamatsu NanoZoomer-XR 2.0-RS C10730 digital scanning system with TDI camera technology a NanoZoomer (Hamamatsu Photonics K.K. Systems, Japan) at 20 × magnification and visualised using NDP.view2 (NanoZoomer Digital Photography).

#### 2.6.2 Semi-automated quantification of Iba1 and GFAP immunostaining

For segmentation of microglia and astrocytes, and extraction of microglial morphometric features, we used custom made software (Matlab) adapted from our previous studies (Ding et al., 2016, Pardon et al., 2016) and applied to the following regions of interest: whole hippocampus, hippocampal CA1, CA2, CA3 and dentate gyrus (DG) subfields. Examples of the semi-automated extraction of regions of interest selection are shown in Suppl. Fig. 5A. This provided the percentage area occupied by glial cells, the number of Iba1- and GFAP- positive cells, used as a measure of microglial and astrocyte density, respectively, and the size of microglial soma, used as a morphometric marker of microglia activation and known to be sensitive to LPS (Kozlowski and Weimer, 2012, Kreisel et al., 2014, Pardon et al., 2016).

### 2.7 Multiplex

Plasma levels of interleukin 1 beta (IL-1β), IL-6, IL-10, interferon gamma (IFN-γ) and tumour necrosis factor alpha (TNF-α) were determined using the Bio-Plex ProTM Mouse Cytokine 23-Plex, Group I assay and Bio-Plex array reader, and analysed using the Bio-Plex Manager Software (Bio-Rad Laboratories, Berkeley, CA, USA) according to the manufacturer’s instructions. The cytokine panel was designed to provide a measure of key cytokines known to respond to LPS and to play a role in AD. IL-1β data were deemed unreliable and are excluded from the results section.

### 2.8 Mass spectrometry

#### 2.8.1 Metabolomic profiling by LC–MS

Hippocampal tissues were weighed and then homogenised with chloroform/methanol/water (1:3:1, 10 µl/mg) using Retsch MM301 ball mill equipment for 3 min. The extraction solvent and sample rack for the ball mill were pre-cooled at −20 °C. The homogenised tissues were mixed vigorously for 1 h at 4 °C and then centrifuged at 15,000*g* for 10 min at 4 °C. After centrifugation, the supernatant was collected and stored at −80 °C prior to LC–MS analysis. A quality control sample was prepared by mixing an equal volume of all samples in order to assess instrument performance (Pereira et al., 2010). Chromatographic separation was performed using a ZIC-pHILIC column (150 mm × 4.6 mm, 5 µm, Merck Sequant). The column was maintained at 45 °C with a flow rate of 300 µl/min as previously described (Surrati et al., 2016). Briefly, the mobile phase consisted of 20 mM ammonium carbonate in water (A) and 100% acetonitrile (B), and the tissue extracts were eluted with a linear gradient over 24 min as follows: 80% B (0 min) to 5% B over 15 min to 5% B with a 2 min linear gradient, followed by re-equilibration with 80% B. A 10 µl injection of each extract was employed for LC–MS analysis. An Exactive MS (Thermo Fisher Scientific, Hemel Hempstead, UK) was used to acquire spectral data in full scan (*m*/*z* 70–1400, resolution 50 000) and both positive and negative electrospray ion modes. The capillary temperature and probe temperature were maintained at 275 and 150 °C, respectively as previously described (Creek et al., 2011).

#### 2.8.2 LC–MS data processing

XCMS was used to pre-process raw LC–MS data for untargeted peak-picking (Tautenhahn et al., 2008) and mzMatch was employed for peak matching and annotation of related peaks (Scheltema et al., 2011). The processed data was then imported into IDEOM for noise filtering and putative metabolite identification (Creek et al., 2012). Metabolite identification was carried out by matching accurate masses and retention times of authentic standards but when standards were not available, accurate masses and predicted retention times were used (Sumner et al., 2007). Metabolites were filtered in IDEOM to have retention time errors of below 35% and mass errors below three parts per million (Vincent et al., 2014).

### 2.9 Data analysis

Data are presented as mean ± SEM (standard error of the mean) and were analysed using InVivoStat (Clark et al., 2012), unless otherwise stated. Baseline behavioural and body weight data, sickness scores, histological and cytokine data were all subjected to 3-way ANOVAs with genotype, sex and treatment, followed, where appropriate, by planned comparisons. To assess the effect of the PBS and LPS challenges on behavioural data and to compare baseline and post-injection data, we used 3-way ANOVAs with genotype, sex and treatment, and repeated measure over time, followed, when appropriate, by planned comparisons. The following pairwise comparisons were decided a priori: i) PBS-treated WT *vs* APP/PS1 mice within each sex to test for genotype differences; ii) PBS-treated males *vs* females within each genotype to test for sex differences; iii) PBS- *vs* LPS-treated mice within each sex and genotype condition to test for differences caused by systemic inflammation with LPS and, where appropriate, iv) baseline *vs* post-injection data within each experimental group to test for the effect of the PBS or LPS challenge. Cytokine and food burrowing data were rank-transformed to normalise the distribution, but presented as non-normalised responses (Deacon, 2012). The number of arm entries was used as a covariate for the analysis of spontaneous alternation performance, in order to control for confounding effects of LPS-induced behavioural suppression.

For LC–MS data, variable selection was performed as a by-product of a classification model. Data were first subjected to multivariate analyses by principal component analysis-class (PCA) and orthogonal partial least squares-discriminant analysis (OPLS-DA), using SIMCA-P version 15.02 (Umetrics AB, Umea, Sweden), in order to detect global metabolic differences between experimental conditions. This was followed by OPLS-DAs applied to models including 2 classes: i) WT *vs* APP/PS1 PBS-treated mice, to identify potential metabolic differences due to the genotype in the absence of immune stimulation, ii) male *vs* female PBS-treated mice, to identify sex-dependent metabolic differences; iii) LPS *vs* PBS for all mice to identify effects global effects of LPS; iv) LPS *vs* PBS for each sex separately to identify sex-dependent metabolic responses to LPS. Mass ions which contributed to separations and clusters were selected according to the variable importance in projection (VIP), a weighted sum of the PLS weight which indicates the importance of the model. VIP values greater than 1.5 were first considered indicative of significant differences between groups. Next, these metabolites were subjected to three-way ANOVAs with genotype, sex and treatment as between subject factors, to confirm the statistical significance of these factors and test for significant interactions between them. Metabolites from this list for which significant overall effects of treatment, or sex X treatment interaction were found, were also considered as potential discriminant of the LPS response within each sex if VIP values from the OPLS-DA models testing the effect of LPS within each sex were greater than 1. This was followed, where appropriate, by planned comparisons, as defined above.

Relationships between behavioural, cytokine, glial and metabolic data, and whether these associations were dependent upon the genotype, sex or treatment, were tested using the Pearson correlation coefficient, for which statistically significant values above 0.7 were considered as strong associations.

P ≤ 0.05 was considered statistically significant for all analyses.

## 3 Results

### 3.1 Systemic LPS-induced sickness

To assess whether APP/PS1 mice responded more strongly to LPS in the early hours after systemic injection than their WT littermates, and to explore the sex dependency of this response, we assessed LPS-induced sickness using physiological measures and by monitoring behavioural suppression from baseline pre-injection performance in two tasks. Results of the three-way ANOVAs on these measures are presented in Suppl. Table 1.

#### 3.1.1 Body mass and rectal temperature

Body mass was overall lower in females regardless of their genotype (F_(1,36)_ = 152.67, p = 0.005; Suppl. Fig. 1A). Within APP/PS1 mice, females (p < 0.0001), but not males (p = 0.87), weighed less than their WT littermates (Genotype × Sex: F_(1,36)_ = 12.22, p = 0.0013) but none of the experimental groups showed significant weight loss 4 h after the LPS or PBS challenge (Suppl. Fig. 1A). Rectal body temperature was overall reduced by LPS (F_(1,34)_ = 17.09, p = 0.0002), but partial comparisons showed that this decrease was only significant in males (minus ∼1.2–1.5 °C, p < 0.05 for both WT and APP/PS1 males compared to PBS-treated males, Fig. 1B).

#### 3.1.2 LPS suppressed food burrowing activity

Food borrowing behaviour was overall suppressed by systemic LPS (p < 0.0001) but unaffected by PBS (p = 0.52; Treatment × Time: F_(1,36)_ = 9.47, p = 0.004, Fig. 1C). Significant reductions in food burrowing behaviour 4 h after injection of LPS were seen in WT males (p = 0.008), WT females (p = 0.002) and APP/PS1 females (p = 0.04), but not in APP/PS1 males (p = 0.10) whose baseline performance was lower than of WT males females and more variable than of APP/PS1 females (Fig. 1C).

#### 3.1.3 LPS suppressed exploratory drive without altering spatial working memory performance

Exploration of the Y maze, assessed through the number of arm visits, did not differ between any experimental groups at baseline (Fig. 1D) but was suppressed by LPS, regardless of the genotype (p < 0.0001 compared to baseline in all cases, Fig. 1E, and p < 0.01 compared to PBS-treated mice in all cases, Suppl. Fig. 1B; Treatment: F_(1,36)_ = 20.66, p < 0.0001). All PBS-treated groups, but female APP/PS1, also showed a milder reduction in Y maze exploration 4 h after injection (p < 0.05 in all cases, Fig. 1E; Treatment × Time: F_(1,36)_ = 58.55, p < 0.0001), reflecting habituation to the apparatus. Spontaneous alternation performance was overall lower in females compared to males (F_(1,35)_ = 4.25, p = 0.048, Fig. 1F) at baseline but not following PBS or LPS administration (Suppl. Fig. 1B), and none of the treatments altered the alternation rate (Fig. 1G).

### 3.2 Systemic LPS-induced circulating cytokines

We assessed systemic inflammation 4 h after inoculation with LPS by quantifying plasma levels of 5 pro- or anti-inflammatory cytokines. Results of the three-way ANOVAs applied to circulating cytokine levels are presented in Suppl. Table 2.

**Fig. 2:**
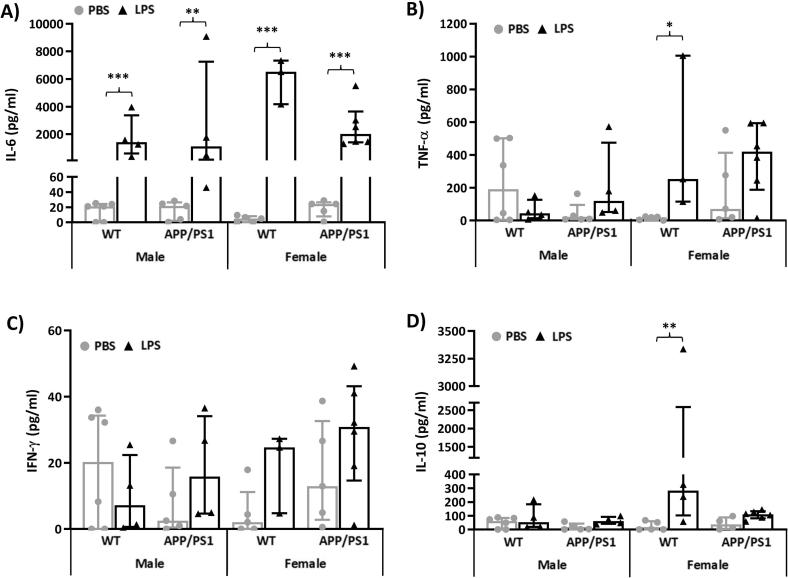
LPS-induced plasma cytokines at 4 h post-injection. 4.5-month-old male and female APP/PS1 mice and their wild-type (WT) littermates were challenged with LPS (100µ/kg *i.v.*) or its vehicle PBS. Their plasma was collected 4 h later, immediately after behavioural assessment, for measurement of induced levels of pro- and anti-inflammatory cytokines. At this time point, a significant increase in circulating Interleukin 6 (IL-6, A), which has both pro- and anti-inflammatory effects, was observed regardless of sex and genotype (A). Levels of the pro-inflammatory cytokine tumour necrosis factor alpha (TNF-α) were increased by LPS in females, particularly WT females (B), but the levels of the other pro-inflammatory mediator, interferon gamma (IFN-γ), were unaltered (C). A significant increase in circulating levels of the anti-inflammatory cytokine IL-10, was also observed in females, regardless of genotype (E). Data were rank-transformed for statistical analyses but are expressed as Median ± interquartile range of non-normalised responses. Dots represent individual animals. Pairwise comparisons: *p < 0.05; **p < 0.01, ***p < 0.0001 *vs* PBS.

We found that, regardless of sex and genotype, LPS led to significant increases in plasma levels of IL-6 (F_(1,30)_ = 116.2, p < 0.0001, post-hoc tests: p < 0.0002 compared to PBS-treated mice in all cases, Fig. 2A), a cytokine known to exert both pro-and anti-inflammatory effects. Elevated levels of the pro-inflammatory cytokine TNF-α after LPS (F_(1,30)_ = 7.82, p = 0.009) were only significant in WT females (p = 0.02 compared to PBS-treated mice, Fig. 2B), whereas LPS-treated females also exhibited significantly higher levels of the anti-inflammatory cytokine IL-10 (Treatment × Sex: F_(1,30)_ = 4.54, p = 0.04), regardless of their genotype (WT: p = 0.0004 and APP/PS1: p = 0.007, compared to PBS-treated mice, Fig. 2D). Circulating INF-γ (F_(1,30)_ = 2.70, p = 0.11, Fig. 2C) levels were unaltered by LPS.

### 3.3 Hippocampal metabolic profiles

#### 3.3.1 Identification of discriminant metabolites

To investigate LPS-induced metabolic changes in the hippocampus, and whether this was dependent upon the genotype and/or and sex of the mice, we used LC–MS analysis. Metabolic data from all hippocampal extracts were first subjected to PCA, to identify trends, and OPLS-DA to detect global metabolic differences between experimental conditions. Then, OPLS-DAs applied to models including 2 classes were carried out in order to identify metabolites differentially expressed in response to LPS or as a function of sex or genotype. The quality of these models was assessed by the R^2^ and Q^2^ parameters which indicate the variance explained by the model and predicted variance after cross-validation, respectively, and range between 0 and 1, with Q2 values above 0.5 (50% of variance predicted) revealing good separation between the classes tested.

Metabolites were considered to contribute to the separations and clusters associated with each experimental condition when their VIP values from OPLS-DA models was greater than 1.5 if subsequent 3-way ANOVAs confirmed their ability to discriminate genotypes, sexes, treatment conditions and/or interactions between these factors. As shown in Table 1, 98 metabolites were identified as potential discriminators of the disease status, sex and/or LPS response, after confirmation with ANOVAs. Their function in the brain and potential implication in sex differences in brain function, AD progression and/or inflammatory processes, when known, is presented in Suppl. Table 3.

**Table 1:** Metabolites differently expressed between males and females and in response to LPS. Statistical significance from 3-way ANOVAs followed by pairwise comparisons testing the effect of LPS within each sex, when appropriate. When significant genotype, sex and/or their interaction with treatment were observed, metabolites with VIP values below 1.5 were considered discriminant if pairwise comparisons confirmed genotype or sex differences in PBS-treated mice and/or a sexually dimorphic LPS response.

				Genotype effect (PBS-treated)			Sex effect (PBS-treated)			Overall LPS effect			LPS effect in males			LPS effect in females		
Exact mass	RT (min)	Putative metabolite Formula	Pathway	VIP	*p*	*vs.* WT	VIP	*p*	*vs.* males	VIP	*p*	*vs.* PBS	VIP	*p*	*vs.* PBS	VIP	*p*	*vs.* PBS
*Amino acid metabolism*																		
131.09	11.79	(3R)-beta-Leucine C6H13NO2	Valine, leucine and isoleucine degradation	1.37			2.04	<0.0001	↘	0.75			0.39			0.64		
103.10	15.33	Choline C5H13NO	Glycine, serine and threonine metabolism	0.83			0.71			1.45			2.00	<0.0001	↗	0.69		
222.07	11.11	l-cystathionine C7H14N2O4S	Glycine, serine and threonine metabolism Methionine metabolism	1.35			1.39	0.01	↘	0.89			0.65			0.88		
149.05	8.79	l-methionine C5H11NO2S	Methionine metabolism	0.69			0.57			2.92	<0.0001	↘	2.14	<0.0001	↘	1.88	<0.0001	↘
165.05	9.32	l-methionine S-oxide C5H11NO3S	Methionine metabolism	0.69			0.83			2.52	<0.0001	↘	1.84	<0.0001	↘	1.77	<0.0001	↘
384.12	9.50	S-adenosyl-l-homocysteine C14H20N6O5S	Methionine metabolism	0.92			1.18			0.91			1.70	0.0001	↗	1.10	0.036	↘
219.07	9.59	O-succinyl-l-homoserine C14H20N6O5S	Methionine metabolism	0.72			1.18			2.04	<0.0001	↘	1.31	0.03	↘	1.50	<0.0001	↘
398.14	11.02	S-adenosyl-l-methionine C15H22N6O5S	Methionine metabolism Arginine and proline metabolism	1.13			0.70			1.82	0.0002	↘	1.30			1.37	0.0002	↘
297.09	6.79	5′-methylthioadenosine C11H15N5O3S	Methionine metabolism Arginine and proline metabolism	1.31			1.57	0.0001	↘	2.44	<0.0001	↘	2.00	<0.0001	↘	1.70	<0.0001	↘
132.05	8.62	N-carbamoylsarcosine C4H8N2O3	Arginine and proline metabolism	0.92			1.07			1.84	0.0002	↗	1.37	0.004	↗	1.21	0.005	↗
231.07	8.97	N-succinyl-l-glutamate 5-semialdehyde C9H13NO6	Arginine and proline metabolism	1.25			1.75	0.0005	↗	0.72			1.34	0.009	↗	1.37	0.005	↘
104.02	9.07	Urea-1-carboxylate C2H4N2O3	Arginine and proline metabolism	1.20			0.60			1.87	<0.0001	↗	1.80	<0.0001	↗	1.07	0.03	↗in WT
188.13	16.71	Homoarginine C7H16N4O2	Arginine and proline metabolism	1.17			1.88	<0.0001	↗	1.06			0.63			1.38		
133.04	10.25	l-aspartate C4H7NO4	Arginine and proline metabolism Lysine biosynthesis	1.00			1.39			1.02			1.78	0.004	↗	0.97		
276.13	10.24	N6-(l-1,3-Dicarboxypropyl)-l-lysine C11H20N2O6	Lysine biosynthesis	1.16			1.52	0.002	↗	0.72			0.32			0.93		
203.08	9.24	N2-acetyl-l-aminoadipate C8H13NO5	Lysine biosynthesis	1.31			1.75	0.001	↗	0.90			0.75			1.14		
161.07	10.10	l-2-aminoadipate C6H11NO4	Lysine biosynthesis	1.44			1.69	0.008	↗	0.80			1.06			0.78		
129.08	9.34	l-pipecolate C6H11NO2	Lysine degradation Alkaloid biosynthesis I	1.38			1.65	<0.0001	↘	0.91			0.86			0.79		
151.06	5.15	(Z)-4-hydroxyphenylacetaldehyde-oxime C8H9NO2	Tyrosine metabolism	1.28			1.85	<0.0001	↘	0.82			0.97			0.57		
190.05	8.17	[FA hydroxy,oxo(7:0/2:0)] 4-hydroxy-2-oxo-Heptanedioic acid C7H10O6	Tyrosine metabolism	1.12			1.59	0.003	↗	0.91			1.30	0.03	↗	1.51	0.0002	↘
165.08	8.00	l-phenylalanine C9H11NO2	Phenylalanine, tyrosine and tryptophan biosynthesis	0.83			0.78			2.19	<0.0001	↗	1.87	<0.0001	↗	1.44	0.003	↗in WT
204.09	9.16	l-tryptophan C11H12N2O2	Phenylalanine, tyrosine and tryptophan biosynthesis Tryptophan metabolism	1.47			1.64	0.09		2.55	<0.0001	↗	1.94	<0.0001	↗	1.67	<0.0001	↗
191.06	9.66	5-hydroxyindoleacetate C10H9NO3	Tryptophan metabolism	1.07			0.84			2.32	<0.0001	↗	2.04	<0.0001	↗	1.31	0.0012	↗
219.11	6.80	Pantothenate C9H17NO5	beta-Alanine metabolism Pantothenate and CoA biosynthesis	1.27			2.06	0.0001	↗	0.75			0.93			1.16		
160.08	6.83	d-alanyl-d-alanine C6H12N2O3	d-Alanine metabolism Peptidoglycan biosynthesis	1.35	0.03	↘	2.08	<0.0001	↘	0.99			0.98			0.72		
612.15	11.03	Glutathione disulfide C20H32N6O12S2	Glutamate metabolism Glutathione metabolism	0.82			1.37			0.77			1.51	0.001	↗	1.10		
140.06	7.25	Methylimidazoleacetic acid C6H8N2O2	Histidine metabolism	0.97			1.40			0.88			1.51	0.002	↗	1.01		
169.08	9.39	N(pi)-methyl-l-histidine C7H11N3O2	Histidine metabolism	1.32			2.00	<0.0001	↘	1.62	<0.0001	↗	1.70	<0.0001	↗	1.59	0.0005	↗

109.02	10.64	Hypotaurine C2H7NO2S	Taurine and hypotaurine metabolism	1.43			2.07	<0.0001	↘	0.95			0.87			0.95		

*Carbohydrate metabolism*																		
118.03	10.42	Succinate C4H6O4	Citrate cycle (TCA cycle) Glyoxylate and dicarboxylate metabolism	1.14			0.65			1.69	0.0003	↘	1.40	0.005	↘	1.18	0.01	↘
192.03	8.66	Isocitrate C6H8O7	Citrate cycle (TCA cycle) Glyoxylate and dicarboxylate metabolism	1.34			1.91	<0.0001	↘	0.96			1.02			0.82		
134.02	10.92	(S)-malate C4H6O5	Citrate cycle (TCA cycle) Pyruvate metabolism Glyoxylate and dicarboxylate metabolism	0.97			0.82			1.46			0.79			1.53	0.0001	↘
90.03	7.25	(d)-lactate C3H6O3	Pyruvate metabolism	0.90			0.95			1.88			1.08			1.55	<0.0001	↘
379.10	9.05	(d)-S-lactoylglutathione C13H21N3O8S	Pyruvate metabolism	0.90			0.93			1.26			0.53			1.63	0.0008	↘
167.98	11.75	Phosphoenolpyruvate C3H5O6P	Citrate cycle (TCA cycle) Pyruvate metabolism Glycolysis/Gluconeogenesis	1.08			1.48	<0.0001	↗	1.23			1.79	0.01	↘	0.78		
88.01	6.81	Pyruvate C3H4O3	Citrate cycle (TCA cycle) Glycolysis/Gluconeogenesis	0.73			0.16			0.70			1.66	0.03	↘	0.53		
170.00	10.31	d-glyceraldehyde 3-phosphate C3H7O6P	Glycolysis/Gluconeogenesis	0.87			0.33			1.70	0.0007	↘	1.10	0.04	↘in APP/PS1	1.28	0.003	↘in APP/PS1
185.99	11.39	3-phospho-d-glycerate C3H7O7P	Glycolysis/Gluconeogenesis Glyoxylate and dicarboxylate metabolism	0.99			1.35	<0.0001	↗	1.10			1.69	0.007	↘	0.67		
155.98	11.41	2-phosphoglycolate C2H5O6P	Glyoxolate and dicatboxylate metabolism	1.28			2.11	<0.0001	↘	1.08			1.54	0.0007	↘	0.71		
206.01	11.83	3-oxalomalate C6H6O8	Glyoxolate and dicatboxylate metabolism	1.28			2.00	<0.0001	↘	0.85			0.91			0.61		
164.07	9.24	l-rhamnose C6H12O5	Fructose and mannose metabolism	0.99			0.78			1.16			1.51	0.002	↗	0.85		
182.08	10.09	d-sorbitol C6H14O6	Fructose and mannose metabolism	1.07			0.96			2.06	<0.0001	↘	1.40	0.002	↘in WT	1.65	<0.0001	↘
276.02	11.62	6-phospho-d-gluconate C6H13O10P	Pentose phosphate pathway	0.79			0.86			1.70	0.0006	↘	1.07	0.08		1.33	0.001	↘
154.00	8.48	Propanoyl phosphate C6H13O10P	Propanoate metabolism C5-Branched dibasic acid metabolism	1.05			0.85			0.98			1.53	0.0009	↗	0.64		
130.03	8.65	Itaconate C5H6O4	C5-Branched dibasic acid metabolism Citrate cycle (TCA cycle)	1.34			1.91	<0.0001	↘	0.94			1.10			0.74		
146.02	9.93	Methyloxaloacetate C5H6O5	C5-Branched dibasic acid metabolism	1.20			1.53	0.006	↘	0.95			0.88			0.94		

*Nucleotide metabolism*																		
136.04	8.58	Hypoxanthine C5H4N4O	Purine metabolism	0.77			0.78			1.00			1.66	0.03	↗	0.94		
168.03	9.07	Urate C5H4N4O3	Purine metabolism	0.22			0.59			2.17	<0.0001	↗	1.77	<0.0001	↗	1.34	0.006	↗
463.07	11.88	N6-(1,2-Dicarboxyethyl)-AMP C14H18N5O11P	Purine metabolism	0.85			0.71			1.52			0.69			1.52	<0.0001	↘
156.02	7.91	Orotate C5H4N2O4	Pyrimidine metabolism	1.21			1.58	0.005	↗	0.83			1.08			1.18	0.01	↘
242.09	6.82	Thymidine C10H14N2O5	Pyrimidine metabolism	0.57			0.26			2.05	<0.0001	↗	1.88	0.002	↗	1.18	0.0007	↗
126.04	6.82	Thymine C5H6N2O2	Pyrimidine metabolism	0.74			0.98			1.87	<0.0001	↗	1.77	0.002	↗	1.03	0.005	↗
114.04	7.17	5,6-dihydrouracil C4H6N2O2	Pyrimidine metabolism Beta-Alanine metabolism Pantothenate and CoA biosynthesis	0.70			0.88			0.77			1.52	0.0007	↗	0.93		

*Lipid metabolism and Fatty acyls*																		
284.27	3.88	Octadecanoic acid C18H36O2	Fatty acids biosynthesis Biosynthesis of unsaturated fatty acids	1.13			0.99			1.50	0.004	↘in WT	0.89			1.46	0.005	↘
256.24	3.91	Hexadecanoic acid C16H32O2	Biosynthesis of unsaturated fatty acids	1.21			1.18			1.52	0.002	↘	0.92			1.35	0.01	↘
306.25	3.88	Icosatrienoic acid C20H34O2	Biosynthesis of unsaturated fatty acids	0.70			094			0.88			0.57			1.41	0.001	↘
304.24	3.88	[FA (20:4)] 5Z,8Z,11Z,14Z-eicosatetraenoic acid C20H32O2	Fatty Acids and Conjugates	0.96			0.85			1.32			0.42			1.62	<0.0001	↘
118.06	5.16	Formyl 3-hydroxy-butanoate C5H10O3	Fatty esters	1.44			2.17	<0.0001	↘	0.97			1.16			0.88		
172.01	10.13	*sn*-glycerol 3-phosphate C3H9O6P	Glycerolipid metabolism Glycerophospholipid metabolism	0.99			1.34			0.98			0.76			1.33	0.0009	↘
306.26	3.88	*sn*-glycero-3-Phosphoethanolamine C5H14NO6P	Glycerophospholipid metabolism Ether lipid metabolism	1.26			1.37			1.79			0.47			1.69	<0.0001	↘
393.29	4.82	PGH2-EA C23H39NO4	Eicosanoids	1.11			1.05			1.73	0.0003	↗	1.40	0.01	↗	1.18	0.005	↗

*Energy Metabolism*																		
506.99	10.98	ATP C10H16N5O13P3	Oxidative phosphorylation Purine metabolism	1.04			0.80			1.97	<0.0001	↘	1.47	0.002	↘	1.28	0.0004	↘
340.00	11.91	d-fructose 1,6-bisphosphate C6H14O12P2	Carbon fixation	1.04			1.54	0.002	↗	1.44	0.0005	↘	1.15	0.05	↘	1.21	0.003	↘
370.01	12.01	d-sedoheptulose 1,7-bisphosphate C7H16O13P2	Carbon fixation	1.23			1.22			1.32			0.81			1.52	<0.0001	↘

*Metabolism of Cofactors and Vitamins*																		
73.02	10.25	Iminoglycine C2H3NO2	Thiamine metabolism	0.71			0.98			1.00			1.56	0.001	↗	0.91		
152.06	6.87	N1-methyl-2-pyridone-5-carboxamide C7H8N2O2	Nicotinate and nicotinamide metabolism	0.81			0.61			2.73	<0.0001	↗	2.25	<0.0001	↗	1.62	<0.0001	↗

*Peptides*																		
276.10	11.03	Gamma glutamylglutamic acid C10H16N2O7	Peptide	0.95			0.94			0.89			1.59	0.005	↗	0.90		
262.08	9.71	l-beta-aspartyl-l-glutamicacid C9H14N2O7	Peptide	1.52	0.03	↘in ♀	0.82			0.65			0.56			0.58		
357.13	7.80	Asp-Ser-His C13H19N5O7	Basic peptide	0.71			0.74			1.54	0.005	↘	0.82			1.36	0.002	↘
508.18	6.81	Asn-Met-Met-Asn C18H32N6O7S2	Hydrophobic peptide	0.52			1.01			1.60			1.57	0.003	↘	0.95		
482.20	8.23	Asp-Phe-Thr-Thr C21H30N4O9	Hydrophobic peptide	1.34	0.03	↗in ♂	1.59	0.003	↗	0.86			0.69			0.64		
360.14	8.79	Asn-Asn-Asn C12H20N6O7	Polar peptide	0.72			0.15			2.87	<0.0001	↘	2.10	<0.0001	↘	1.85	<0.0001	↘

*Biosynthesis of Polyketides and nonribosomal Peptides*																		
509.33	4.64	Narbomycin C28H47NO7	Biosynthesis of 12-, 14- and 16-membered macrolides	0.61			0.29			2.14	<0.0001	↗	1.44	0.003	↗	1.50	<0.0001	↗
515.18	11.20	13-dihydrocarminomycin C26H29NO10	Biosynthesis of type II polyketide products	1.21			1.71	0.004	↗	0.84			0.86			0.69		

*Biosynthesis of Secondary metabolites*																		
200.08	7.89	Dihydroclavaminic acid C8H12N2O4	Clavulanic acid biosynthesis	1.27			2.04	<0.0001	↘	0.80			0.79			0.83		

*Not known*																		
102.08	16.19	γ-aminobutyramide C4H10N2O	N known	1.23			1.73	0.004	↗	0.77			1.48	0.003	↗	1.37	0.006	↘
274.05	10.27	1-deoxy-d-altro-heptulose 7-phosphate C7H15O9P	Not known	1.13			1.19			1.24			1.65	0.01	↗	0.58		
281.11	10.68	1-methyladenosine C11H15N5O4	Not known	1.54	0.03	↗in ♀	0.61			0.1			0.79			0.53		
367.27	4.95	3,5-tetradecadiencarnitine C21H37NO4	Not known	1.06			1.24			1.57	0.002	↗	1.15	0.01	↗	1.44	0.04	↗
181.99	9.73	3-methylphosphoenolpyruvate C4H7O6P	Not known	1.20			1.77	<0.0001	↘	0.88			0.75			0.83		
181.10	8.58	6-methyltetrahydropterin C7H11N5O	Not known	1.00			1.11			0.83			1.79	0.0006	↗	1.05	0.02	↘
430.20	5.36	Athamantin C24H30O7	Not known	1.31			1.79	0.01	↗	0.85			0.78			0.65		
348.11	9.24	Camptothecin C20H16N2O4	Not known	0.97			0.72			1.59	0.02	↘	1.37	0.009	↘in WT	1.11		
158.06	4.39	Dimethyl citraconate C7H10O4	Not known	0.97			1.72	0.0005	↘	0.53			0.92			1.11		
159.13	9.46	dL-2-sulfoctanoicacid C8H17NO2	Not known	1.09			1.67	0.0002	↗	0.60			0.41			0.88		
425.35	4.65	Elaidiccarnitine C25H47NO4	Not known	1.20			1.44			1.53	0.004	↗	1.19	0.04	↗in APP/PS1	1.14	0.03	↗in WT
275.14	8.59	Glutarylcarnitine C12H21NO6	Not known	1.36			1.89	0.007	↗	0.68			0.70			0.87		
246.05	8.65	Glycerophosphoglycerol C6H15O8P	Not known	1.28			1.52	0.003	↗	1.29			0.68			1.49	0.0001	↘
423.33	4.68	Linoelaidylcarnitine C25H45NO4	Not known	1.28			1.42			1.52	0.003	↗	1.24	0.02	↗	0.98	0.04	↗
216.12	10.34	N-acetyl-(l)-arginine C8H16N4O3	Not known	1.15	0.03	↘in ♀	1.53	0.005	↗	0.87			0.76			1.28	0.0008	↘
202.14	14.08	NG,NG-dimethyl-l-arginine C8H18N4O2	Not known	1.22			0.97			1.32			1.60	0.001	↗	0.81		
243.09	8.89	Nocardicin C C23H26N4O8	Not known	0.91			0.71			1.65			0.71			1.53	<0.0001	↘
175.03	5.16	Nonulose 9-phosphate C9H19O12P	Not known	1.15			1.48	0.004	↘	1.81			1.91	<0.0001	↘	0.70		
249.03	12.35	Norepinephrinesulfate C8H11NO6S	Not known	0.97			1.87	0.003	↗	0.75			1.35	0.008	↗	0.72		
288.06	8.85	Orotidine C10H12N2O8	Not known	1.29			1.56	0.0003	↗	0.92			0.59			1.19	0.007	↘
371.30	4.84	Tetradecanoylcarnitine C21H41NO4	Not known	1.22			1.53	0.04	↘	1.00			0.90			0.77		
573.09	8.83	GDP-3,6-dideoxy-d-galactose C16H25N5O14P2	Not known	1.17			0.81			1.10			1.79	0.002	↗	0.63		
133.07	6.76	N-hydroxyvaline C5H11NO3	Linamarin biosynthesis	1.29			1.70	<0.0001	↘	2.45	<0.0001	↘	2.14	<0.0001	↘	1.61	<0.0001	↘

PBS: Phosphate-buffered saline; WT: wild-type; ♀: female; ♂: male.

##### 3.3.1.1 Global metabolic differences reveal distinct clustering between PBS- and LPS treated mice

**Fig. 3:**
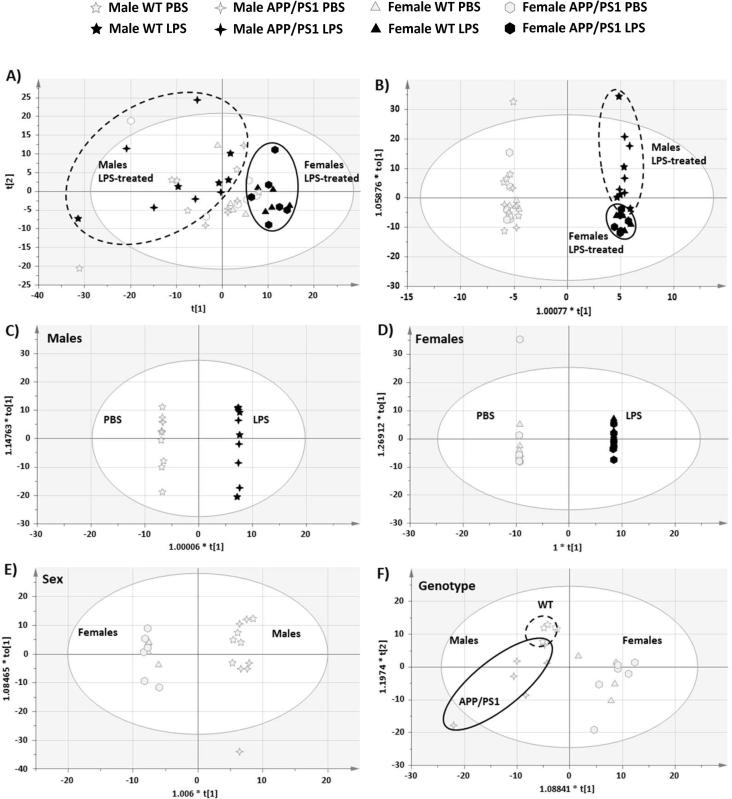
Score plots of Principal Component Analysis (PCA) and two-class Orthogonal Partial Least Square Discriminant Analysis (OPLS-DA) models for hippocampal metabolism at 4 h post-injection with LPS or PBS. R^2^: variance explained, Q^2^: variance predicted. Dots represent individual animals. A). PCA analysis reveals global metabolic differences between LPS-treated males and females regardless of genotype [PC1 (X axis): R^2^X[1] = 0.232, Q^2^ = 0.115; PC2 (Y axis): R*^2^*X[2] = 0.123, Q^2^ = 0.082). B) Global OPLS-DA model (R^2^ = 0.994, Q2 = 0.886) revealing clear separations between PBS- and LPS-treated mice (X axis: predictive component), but also some separation between LPS-treated male and female mice (Y axis: first orthogonal component), regardless of genotype. C) Accordingly, the class OPLS-DA model comparing genotypes in PBS-treated mice confirmed the lack of clear separation between WT and APP/PS1 mice (R^2^ = 0.697, Q^2^ = 0.244). Predictive component 1 (X axis) *vs* 2 (Y axis). 2-class OPLS-DA models confirmed the strong differences in hippocampal metabolism due to sex in the absence of immune stimulation, (R^2^ = 0.985, Q^2^ = 0.809; D) as well as the excellent separation between PBS- and LPS-treated mice males (R^2^ = 1.00, Q^2^ = 0.857; E) and females (R^2^ = 1.00, Q2 = 0.863, F). D-F: Predictive (X axis) *vs* 1st orthogonal (Y axis) component.

PCA analysis preformed on all animals gave 6 components explaining 59.6% of the variance. The plots pertaining to the first two components revealed, as the major trend, a separation between LPS-treated males and females (Fig. 3A). This was confirmed by the global OPLS-DA which gave 5 components (1 predictive and 4 orthogonal) with a variance explained (R2) of 99.4% and predictive variance (Q2) of 88.6%. As shown on Fig. 3B, a clear separation was found between PBS- and LPS-treated male and female WT and APP/PS1 mice 4 h after the immune challenge, indicating that metabolic changes rapidly occurred in response to LPS, regardless of sex or disease status. Within LPS-treated mice, some separation between sexes was also seen, regardless of genotype (Fig. 3B), suggesting that the response to LPS was in part, sex-dependent. Metabolic differences between genotypes were not apparent. Thirty-seven metabolites with VIP values above 1.5 were identified from the global OPLS-DA model. Thirty-two of them showed statistically significant overall effects of treatment, revealing major changes in amino acids, carbohydrate, nucleotide, lipid and energy metabolism in response to LPS, regardless of sex and genotype (Table 1).

Subsequent 2-class OPLS-DAs between PBS- and LPS-treated mice within each sex, also gave strong models with a variance explained of 100% and a predicted variance above 85%. Five components were identified in males (1 predictive + 4 orthogonal; R^2^ = 1.00, Q^2^ = 0.857) and 7 in females (1 predictive and 6 orthogonal; R^2^ = 1.00, Q^2^ = 0.863). Loading plots of predictive *vs* first orthogonal components revealed a clear separation between treatment groups, regardless of genotype, in both sexes (Fig. 3C&D, for males and females, respectively). The hippocampal metabolic response of males to LPS was characterised by significant changes in 53 metabolites (Table 1). Thirty-six discriminant metabolites with VIP values above 1.5 were identified from the 2-class OPLS-DA between PBS- and LPS-treated males, and confirmed with ANOVAs. Statistical significance between these groups was also confirmed for another 13 metabolites identified from the global OPLS-DA model, and for 4 the 11 metabolites with confirmed Sex × Treatment interaction effects. The hippocampal metabolic response to LPS in females was characterised by statistically significant changes in 50 metabolites (Table 1). Twenty discriminant metabolites with VIP values above 1.5 were identified from the 2-class OPLS-DA model, and confirmed with ANOVAs. Statistical significance between PBS- and LPS-treated females was also confirmed for another 20 metabolites identified from the global OPLS-DA model, and for 9 of the 11 metabolites showing sexually dimorphic responses to LPS.

##### 3.3.1.2 Discriminant metabolites between sexes in PBS-treated mice

Since the metabolic response to LPS was found to be, at least in part, sex-dependent, a 2-class OPLS-DA was also carried out between PBS-treated males and females in order to identify whether the hippocampal metabolic profile of males and females differs in the absence of immune stimulation. This gave a strong model with 1 predictive and 2 orthogonal components (R^2^ = 0.985, Q^2^ = 0.809) and clear separation between sexes, regardless of genotype (Fig. 3E). Sex differences in hippocampal metabolism were characterised by significant changes in the levels of 40 metabolites, showing major differences in amino acids, carbohydrate and fatty acyls metabolism (Table 1). While forty-three metabolites with VIP values above 1.5 were identified from the 2-class OPLS-DA model, 36 were confirmed with statistically significant sex effects in PBS-treated mice. Sex differences in PBS-treated mice were also confirmed for another 4 metabolites for which significant effects of sex or sex × treatment interaction were revealed by individual ANOVAs.

##### 3.3.1.3 Lack of major metabolic perturbations in the hippocampus of 4.5-month-old APP/PS1 mice

Next we carried out a 2-class OPLS-DA between genotypes in PBS-treated mice to confirm the lack of apparent differences in the hippocampal metabolic profile of WT and APP/PS1 mice. This gave a weak model explaining 24.4% of the variance between genotypes (3 predictive, 0 orthogonal components; R^2^ = 0.697, Q^2^ = 0.244), revealing a lack of complete separation between WT and APP/PS1 mice (Fig. 3F). This indicates that the metabolic profile of PBS-treated WT and APP/PS1 mice was not strongly influenced by the disease status, consistent with our previous study in males showing a lack of clear differences in hippocampal metabolism between WT and APP/PS1 mice at 4 and 8 months of age (Maroof et al., 2014).

Accordingly, only 2 metabolites with VIP values above 1.5 could be identified with this 2-class OPLS-DA model and confirmed with ANOVAs. Significant genotype effects were also found for another 3 out of the 98 validated metabolites, with confirmed statistical significance within PBS-treated mice (Table 1). Although some separation in hippocampal metabolism appear to be emerging between 4.5-months-old WT and APP/PS1 males (Fig. 3F), statistically significant genotype differences were predominantly seen in females (Suppl. metabolomics results and Suppl. Fig. 2). This apparent distinct clustering, which cannot be explained by orthogonal variation within males, may be due to a combination of borderline differences that are not sufficiently severe to reach statistical significance in individual ANOVAs. Indeed, 59 additional metabolites showed VIP values comprised between 1 and 1.5 (Table1).

#### 3.3.2 Metabolic pathways with sex differences and responsive to systemic LPS

**Fig. 4:**
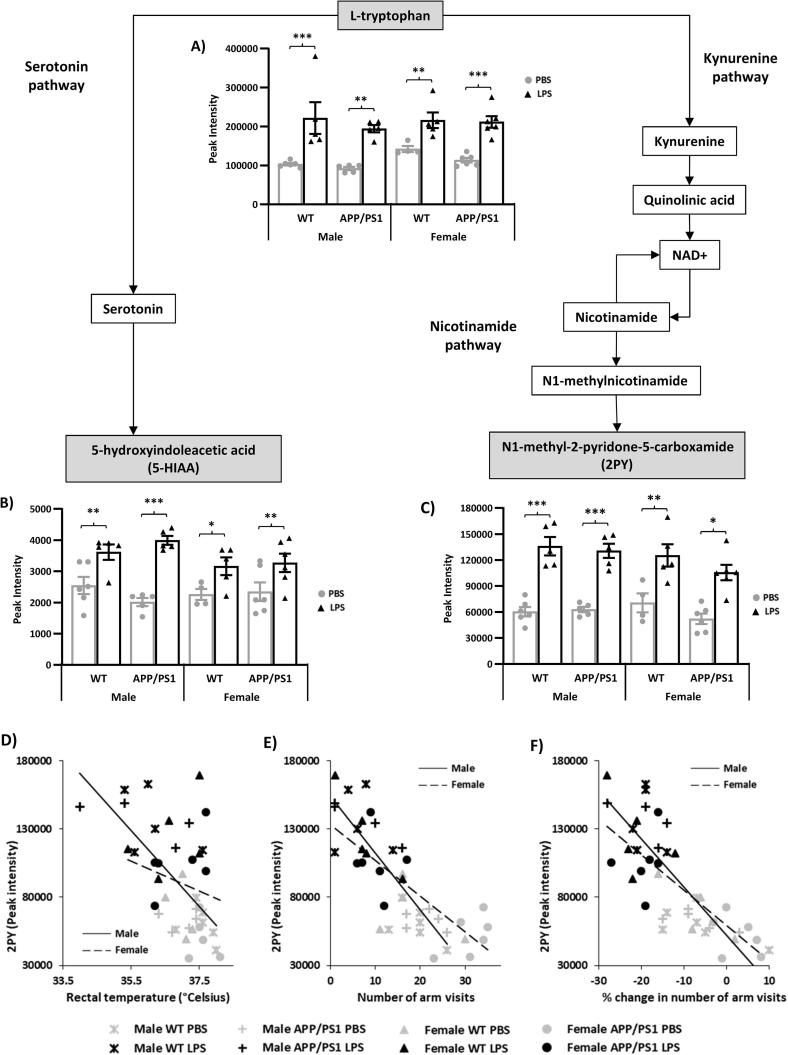
Increased hippocampal tryptophan metabolism 4 h after systemic LPS administration. 4.5-month-old male and female APP/PS1 mice and their wild-type (WT) littermates were challenged with LPS (100µ/kg *i.v.*) or its vehicle PBS. Schematic representation of the anti-inflammatory serotonin, and pro-inflammatory kynurenine, pathways of tryptophan metabolism. At 4 h post-injection, LPS-treated mice showed significant upregulation of l-tryptophan (A) as well as of 5-Hydroxylindoleacetic acid (B) and N1-Methyl-2-pyridone-5-carboxamide (2PY, C), the end metabolites of the serotonin and kynurenine pathways, respectively. Changes in 2PY levels were negatively correlated to D) rectal temperature in males (r = −0.718, p = 0.0004) which exhibited an hypothermic response to LPS but not females 9r = -0.21, p = 0.37); E) the number of arms visited in the spontaneous alternation test 4 h after the injection in both males (r = −0.837, p < 0.0001) and females (r = -0.791, p < 0.0001); and F) sickness scores for arm visits in both males (r = −0.741, p < 0.0001) and females (r = −0.824, p < 0.0001), suggesting that increased 2PY levels is associated with the severity of LPS-induced sickness. Data are expressed as Means ± SEM. Dots represent individual animals. Discriminant metabolites are highlighted by grey text boxes. Pairwise comparisons following 3-way ANOVAs: *, p < 0.05; **, p < 0.01; **, p < 0.0001 compared to PBS-treated mice of same sex and genotype.

**Fig. 5:**
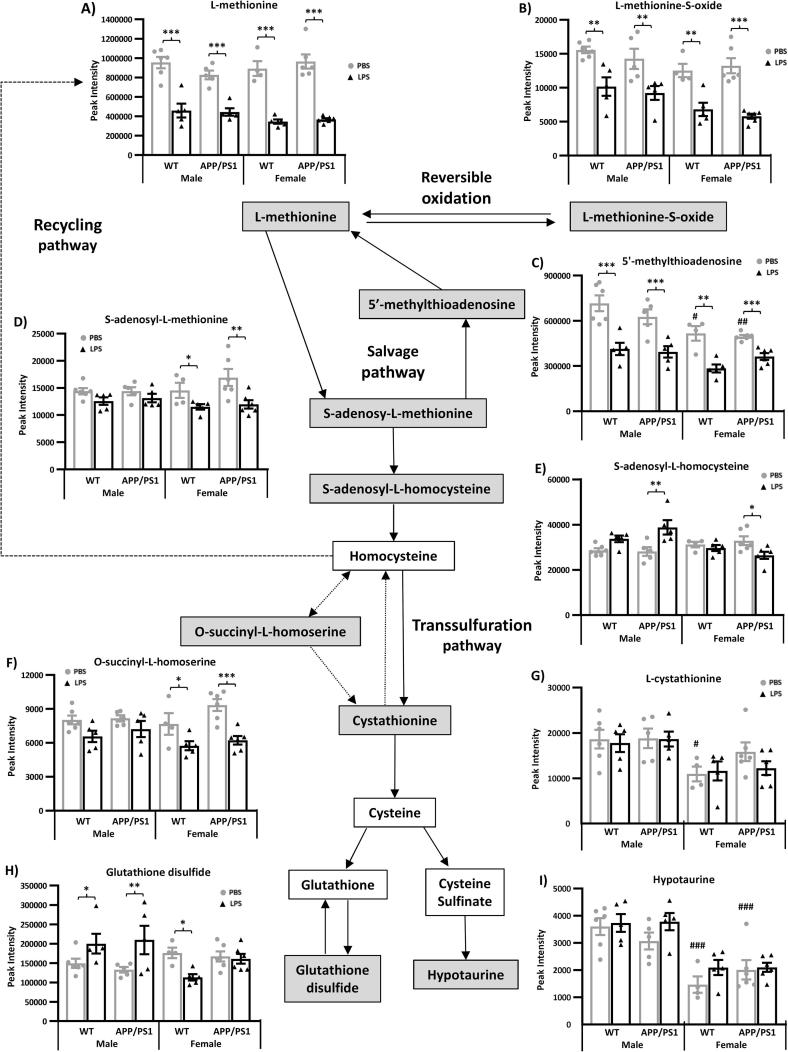
Reduced hippocampal methionine metabolism 4 h after systemic LPS administration. 4.5-month-old male and female APP/PS1 mice and their wild-type (WT) littermates were challenged with LPS (100 µ/kg *i.v.*) or its vehicle PBS. Schematic representation of methionine metabolism showing downregulation of 4 key metabolites from this pathway in LPS-treated mice, at 4 h post-injection, regardless of sex or genotype (A–D). Two of these metabolites, l-methionine-S-Oxide (B) and 5′-Methylthioadenosine (D), as well as 2 methionine derivatives involved in taurine metabolism, l-Cystathionine (E) and hypotaurine (F), were also found in significantly reduced levels in females compared to males. Data are expressed as Means ± SEM. Dots represent individual animals. Discriminant metabolites are highlighted by grey text boxes. Pairwise comparisons following 3-way ANOVAs: *, p < 0.05; **, p < 0.01; **, p < 0.0001 compared to PBS-treated mice of same sex and genotype; ^++^, p < 0.01, ^+++^, p < 0.0001 compared to males. ^#^, p < 0.05; ^##^, p < 0.01; ^###^, p < 0.0001; compared to PBS-treated males of same genotype.

**Fig. 6:**
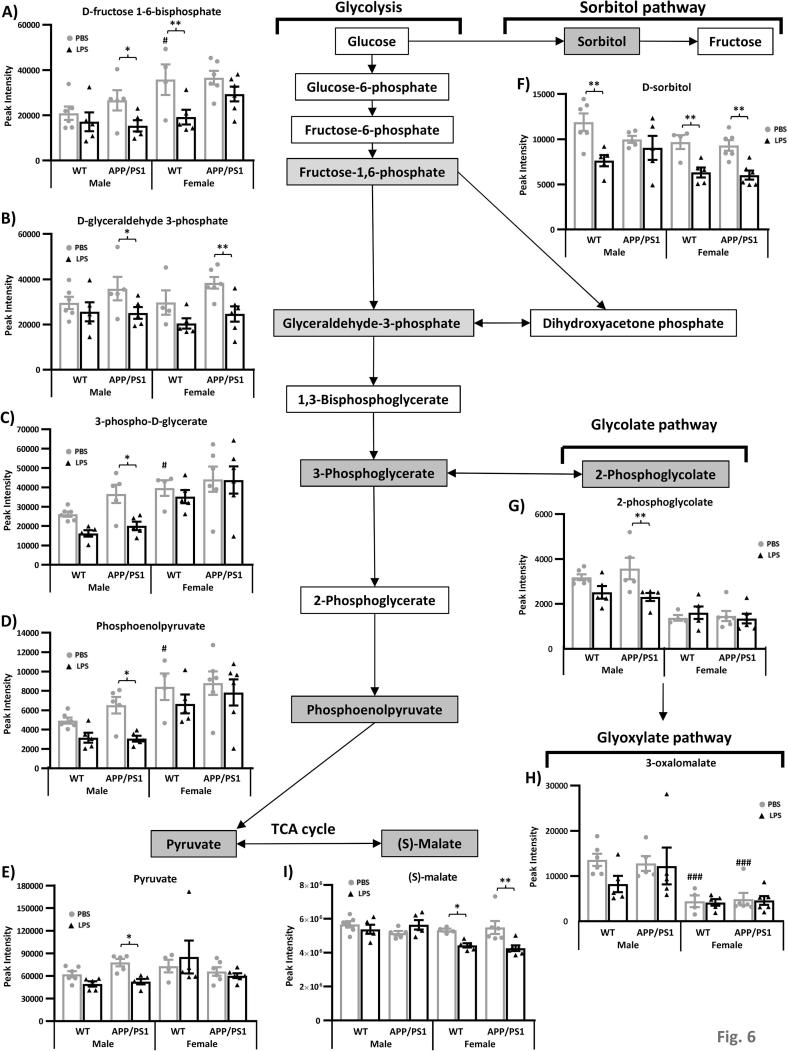
Reduced pyruvate metabolism in the hippocampus of APP/PS1 male 4 h after systemic LPS administration. 4.5-month-old male and female APP/PS1 mice and their wild-type (WT) littermates were challenged with LPS (100 µ/kg *i.v.*) or its vehicle PBS. Schematic representation of the pyruvate metabolic pathway and its links with the sorbitol and glycolate pathways. At 4 h post-injection, LPS-treated APP/PS1 male mice failed to show a reduction in d-sorbitol levels (A), but in contrast, exhibited downregulation of 4 key metabolites of the pyruvate metabolic pathway: 3-Phospho-d-glycerate (B), 2-phosphoglycolate (C), phosphoenolpyruvate (D) and pyruvate (E). Data are expressed as Means ± SEM. Dots represent individual animals. Discriminant metabolites are highlighted by grey text boxes. Pairwise comparisons following 3-way ANOVAs: *, p < 0.05; **, p < 0.01; compared to PBS-treated mice of same sex and genotype. ^#^, p < 0.05; ^##^, p < 0.01; ^###^, p < 0.0001; compared to PBS-treated males of same genotype.

**Fig. 7:**
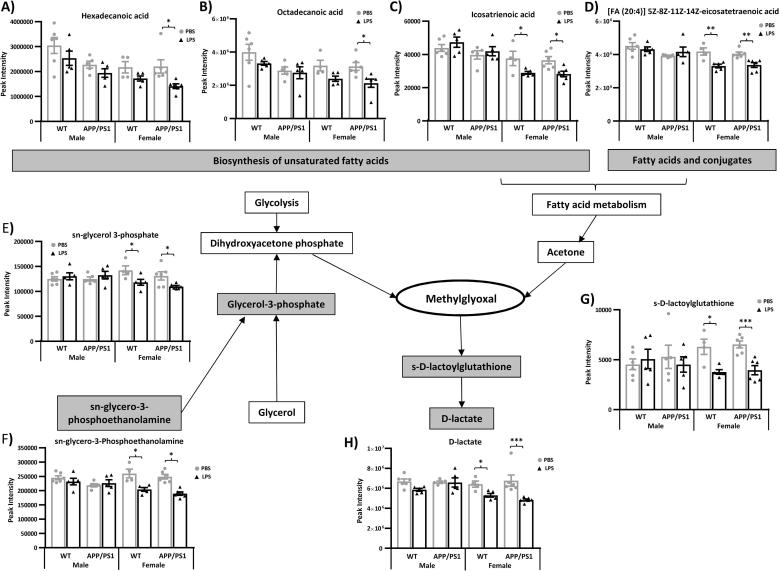
Reduced methylglyoxal metabolism in the hippocampus of WT and APP/PS1 female 4 h after systemic LPS administration. 4.5-month-old male and female APP/PS1 mice and their wild-type (WT) littermates were challenged with LPS (100 µ/kg *i.v.*) or its vehicle PBS. Schematic representation of the main pathways regulating methylglyoxal metabolism. At 4 h post-injection, LPS-treated APP/PS1 female mice showed a reduction in lipid metabolism, with downregulation of 5 key metabolites involved in fatty acid and glycerolipid metabolism: hexadecanoic acid (A), octadecanoic acid (B), icosatrienoic acid (C), [FA (17:0)] heptadecanoic acid (D) and *sn*-Glycerol 3-phosphate (E). This was associated with reduced levels of (d)-S-Lactoylglutathione (F) and (d)-Lactate (G), the reduction products of methylglyoxal. Data are expressed as Means ± SEM. Dots represent individual animals. Discriminant metabolites are highlighted by grey text boxes. Pairwise comparisons following 3-way ANOVAs: *, p < 0.05; **, p < 0.01; ***, p < 0.0001 compared to PBS-treated mice of same sex and genotype. ^#^, p < 0.05; compared to PBS-treated males of same genotype.

The analyses revealed that regardless of sex and disease status, LPS predominantly affected the activity of four metabolic pathways: tryptophan (Fig. 4) and methionine (Fig. 5), regardless of sex and disease status, pyruvate in males (Fig. 6) and methylglyoxal in females (Fig. 7); while sex differences were also found in the absence of immune stimulation within the methionine (Fig. 5) and pyruvate (Fig. 6) metabolic pathways.

Changes in other metabolites as well as their role in brain function and implications in sex differences, AD progression and immune processes, are reported in Suppl. metabolomics results, Suppl. Fig. 3 and Suppl. Table 3, respectively.

##### 3.3.2.1 LPS-induced tryptophan metabolism regardless of sex and disease status

Tryptophan metabolic pathways are represented Fig. 4. Tryptophan is an essential amino acid involved in protein synthesis and substrate of a number of bioactive substances. It is the precursor of the monoaminergic neurotransmitter serotonin which plays a critical beneficial role in modulating behaviour, cognition, mood, stress and inflammatory responses (Hoglund et al., 2019). The majority of tryptophan is, however, catabolised by the kynurenine pathway, the first part of the tryptophan nicotinamide pathway (Fukuwatari and Shibata, 2013), which has been linked to impaired behavioural and stress responses, and proinflammatory changes to the brain (Hoglund et al., 2019). Kynurenine metabolism leads to activation of nicotinamide adenine dinucleotide (NAD) metabolism, an important regulator of various energy metabolism pathways and cellular homeostasis, *via* the biosynthesis of quinolinic acid, forming the second part of the tryptophan-nicotinamide pathway (Fukuwatari and Shibata, 2013, Yaku et al., 2018).

Both the serotonin and nicotinamide pathways of tryptophan metabolism, illustrated Fig. 4, were found to be stimulated in the hippocampus of LPS-treated mice. This was reflected by elevated l-tryptophan levels (Fig. 4A), associated with higher levels of 5-Hydroxylindoleacetate (5-HIAA), the end product of the serotonin pathway of tryptophan metabolism, as well as of N1-methyl-2-pyridone-5-carboxamide (2PY, Fig. 4B&C), a toxic degradation product of nicotinamide (Lenglet et al., 2016) whose levels reflect the amount of nicotinamide biosynthesized from tryptophan (Shibata and Matsuo, 1990) and correlate with upstream activation of the kynurenine pathway of tryptophan metabolism (Mayneris-Perxachs et al., 2016).

Correlation analyses indicated that fluctuations in 2PY levels were associated with a number of parameters related to the sickness response to LPS. We found negative associations between 2PY levels and i) rectal temperature (Fig. 4D) in males, which exhibited LPS-induced hypothermia (males: r = −0.718, p = 0.0004; females: r = −0.21, p = 0.37); ii) the number of arms visited in the spontaneous alternation test 4 h after the injection (r = −0.80, p < 0.0001; Fig. 4E), in both males (r = −0.837, p < 0.0001) and females (r = −0.791, p < 0.0001); and iii) sickness scores for arm visits (r = −0.773, p < 0.0001; Fig. 4F) in both males (r = −0.741, p < 0.0001) and females (r = −0.824, p < 0.0001).

##### 3.3.2.2 LPS-induced alterations in methionine metabolism are in part sex-dependent

Methionine is an essential amino acid involved in protein synthesis and required for growth and tissue repair, immune responses, protection against oxidative stress as well as epigenetic regulation in the brain (Martinez et al., 2017, McGowan et al., 2008). It is also a substrate for other key amino acids, such as taurine and cysteine, as well as the antioxidant molecule glutathione (Fig. 5).

Significant reductions in l-methionine (Fig. 5A), l-methionine S-oxide (Fig. 5B), a toxic oxidation product of methionine (Stadtman et al., 2005), and 5′-methylthioadenosine (Fig. 5C), a methionine precursor in the salvage pathway (Christa et al., 1986), indicated that LPS attenuated the production and metabolism of l-methionine, regardless of sex and disease status.

Levels of S-adenosy-l-homocysteine, an intermediate in methionine biosynthesis and degradation by the recycling and transsulfuration pathways, respectively, were increased by LPS in APP/PS1 males with opposite effects seen in APP/PS1 females (Genotype × Sex × Treatment: F_(1,34)_ = 4.13, p = 0.49; Fig. 5E). Methionine is a substrate for the anti-oxidant molecule glutathione whose toxic oxidation product glutathione disulfide (Stadtman et al., 2005) was more found more abundant in the hippocampus of LPS-treated males, regardless of genotype, but less abundant in the hippocampus of WT females (Sex × Treatment: F_(1,34)_ = 14.52, p = 0.0006, Fig. 5H).

Effects of LPS were more pronounced in females which also showed downregulation of other metabolites involved in the synthesis of methionine *via* both the salvage and recycling pathways. LPS-treated females exhibited reduced hippocampal levels of S-adenosy-l-methionine (Fig. 5D), an intermediate in methionine salvage also involved in the synthesis of homocysteine, key intermediate in methionine metabolism located at the branch point between the recycling pathway and transsulfuration pathway, as well as of O-succinyl-l-homoserine (Fig. 5F), also involved in l-methionine recycling and degradation *via* modulation of homocysteine biosynthesis (Flavin and Slaughter, 1967).

In the absence of immune stimulation, females also presented with reduced levels of l-5′-methylthioadenosine (Fig. 5C), the first step in the methionine salvage pathway, as well as l-cystathionine and hypotaurine (Fig. 5G&I, respectively), two methionine derivatives and key intermediates in the synthesis of taurine, an amino acid found in very high concentrations in most cells (Schaffer and Kim, 2018); but l-methionine levels were not affected by sex differences (Fig. 5A).

##### 3.3.2.3 LPS lowers pyruvate metabolism in APP/PS1males

Pyruvate is a key metabolite in several metabolic pathways important for glucose and energy homeostasis, with potent anti-oxidant and anti-inflammatory properties (Das, 2006). It is made from glucose and is the end-product of glycolysis (Fig. 6).

In males, and more specifically APP/PS1 males, LPS rapidly lowered pyruvate metabolism by downregulating several intermediates in the glycolysis pathway. d-fructose 1,6-bisphosphate (Fig. 6A), and downstream metabolites, d-glyceraldehyde 3-phosphate (Fig. 6B), 3-phospho-d-glycerate (Fig. 6C), phosphoenolpyruvate (Fig. 6D) and ultimately of pyruvate (Fig. 6E) were all significantly less abundant in the hippocampus of LPS-treated APP/PS1 males 4 h after LPS administration. A baseline, however, there was a trend for these metabolites to be more abundant in the hippocampi of APP/PS1 males, explaining the greater effect of LPS, but post-LPS levels of these intermediates in pyruvate metabolism were similar in males from both genotypes. This was associated with reduced levels of 2-phosphoglycolate (Sex × Treatment: F_(1,34)_ = 7.63, p = 0.009, Fig. 6G), which can be converted into the glycolytic intermediate 3-phospho-d-glycerate (Fig. 6C) *via* activation of glycolate metabolism. Conversion of glucose into fructose is a two-step process in which glucose is reduced to sorbitol, which is then converted to fructose. LPS-treated APP/PS1 males also failed to show the decreased in d-sorbitol contents observed in all other LPS-treated groups (WT males, WT and APP/PS1 females, Fig. 6F). In females of both genotypes, LPS also reduced the levels of S-malate (Sex × Treatment: F_(1,34)_ = 11.62, p = 0.0017, Fig. 6I), a metabolite of the KREBS cycle, which can be recycled into pyruvate.

##### 3.3.2.4 LPS lowers methylglyoxal metabolism in females

Methylglyoxal is a neurotoxic by-product of glycolysis, fructose, fatty acid and protein metabolism and potent inducer of inflammation and oxidative stress which can be detoxified by degradation in d-lactate *via* conversion into d-S-lactoylglutathione [(Allaman et al., 2015, Desai et al., 2010), Fig. 7].

In females, LPS induced a downregulation of a number of metabolites upstream and downstream of methylglyoxal production. This includes d-sorbitol, which is involved in fructose metabolism (Fig. 6F), metabolites involved in the biosynthesis of unsaturated fatty acids, particularly in APP/PS1 females [hexadecanoic acid, octadecanoic acid, icosatrienoic acid (Sex × Treatment: F_(1,34)_ = 10.67, p = 0.002), Fig. 7A-C, respectively], the fatty acid and conjugate [FA (20:4)] 5Z,8Z,11Z,14Z-eicosatetraenoic acid (Sex × Treatment: F_(1,34)_ = 11.42, p = 0.0018, Fig. 7D) as well as *sn*-glycerol 3-phosphate (Sex × Treatment: F_(1,34)_ = 10.94, p = 0.002, Fig. 7E), which is synthesised by both glycerol and *sn*-glycero-3-phosphoethanolamine (Sex × Treatment: F_(1,34)_ = 18.49, p = 0.002, Fig. 7F) to form dihydroxyacetone phosphate and ultimately, methylglyoxal. This was associated with reduced levels of its degradation product (d)-S-lactoylglutathione and (d)-lactate (Sex × Treatment: F_(1,34)_ = 6.17, p = 0.02 & F_(1,34)_ = 5.62, p = 0.02; Fig. 7G&H, respectively).

### 3.4 Lack of glial response to LPS at 4 h post-injection

We used immunohistochemistry to detect Iba-1 positive cells, quantify their number, the area they occupied and the size of their soma (used as a morphological marker of microglial activation) and to determine the area occupied by GFAP-positive astrocytes, in the hippocampus of 4.5-month-old male and female WT and APP/PS1 mice 4 h after LPS or PBS administration. Results of the three-way ANOVAs applied to these measures are presented in Suppl. Table 4.

**Fig. 8:**
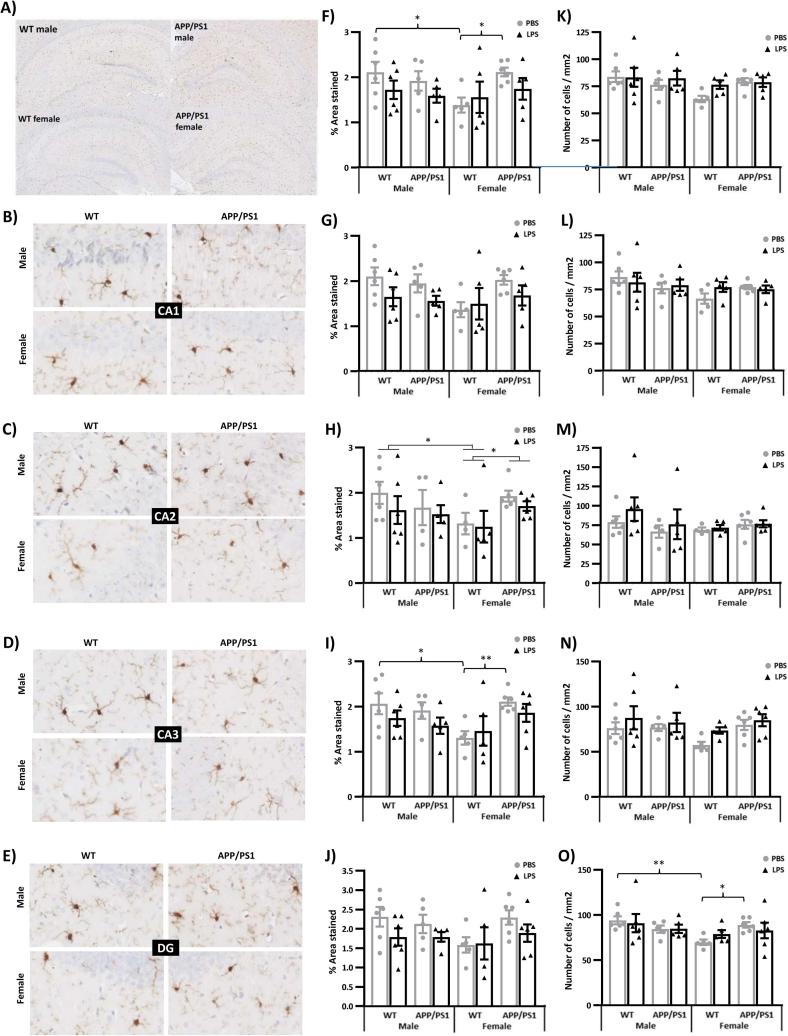
Lack of microglial response to LPS in the hippocampus at 4 h post-injection. 4.5-month-old male and female APP/PS1 mice and their wild-type (WT) littermates were challenged with LPS (100 µ/kg *i.v.*) or its vehicle PBS. Their brain were collected 4 h later, immediately after behavioural assessment, and one hemisphere was processed for immunostaining of Iba1 positive microglia. Representative images of Iba1 immunostaining in the whole hippocampus (A), CA1 (B), CA2 (C), CA3 (D), and dentate gyrus (DG, E) subfields extracted and analysed using a Matlab tool. LPS had no significant effects on microglial density in any hippocampal areas, estimated through the quantification of the percentage area covered by Iba1 positive microglia (F–J) and number of microglial cells per mm^2^ (K–O). The area covered by microglia, was, however, significantly lower in the hippocampus of WT females (F), particularly in the CA2 (H) and CA3 (G) subfields, but lower microglial numbers were only observed in the dentate gyrus (O). Data are expressed as Means ± SEM. Dots represent individual animals. Pairwise comparisons: *p < 0.05; **p < 0.01.

We report that the area occupied by Iba-1 positive microglia was lower in the hippocampus of WT female mice compared to WT males and APP/PS1 females (Genotype × Sex: F_(1,35)_ = 4.14, p = 0.049, Fig. 8A&F), with significant reductions being particularly evident in the CA2 (Genotype × Sex: F_(1,34)_ = 4.24, p = 0.047, Fig. 8C&H) and CA3 (Genotype × Sex: F_(1,36)_ = 7.37, p = 0.01, Fig. 8D&I) subfields. The smaller area covered by microglia seen in WT females was particularly evident in PBS-treated mice for both the whole hippocampus (p = 0.02 *vs* WT males and APP/PS1 females, Fig. 8F) and CA3 subfield (p = 0.01 *vs* WT males and p = 0.007 APP/PS1 females, Fig. 8I). LPS caused non-significant reductions in the area covered by Iba-1 throughout the hippocampus of APP/PS1 females, as well as of males from both genotypes (Fig. 8F–J). We also found a lower number of Iba1 positive cells in the DG of PBS-treated females compared to PBS-treated WT males (p = 0.008, Fig. 8O) and APP/PS1 females (p = 0.03, Genotype × Sex: F_(1,36)_ = 5.02, p = 0.03, Fig. 8O). The area of microglial somas did not differ between the sex, genotype and treatment conditions in any of the hippocampal subfields (Suppl. Fig. 5B–F), and there were very few microglial clusters, albeit significantly more in the hippocampi of APP/PS1 mice compared to their WT littermates (F_(1,35)_ = 10.05, p = 0.003; Suppl. Fig. 5G), consistent with the relatively low Aβ plaque load at 4.5 months of age (Suppl. Fig. 5H&I).

We did not detect differences in the area occupied by GFAP after LPS in discrete hippocampal regions of 4.5-month-old male and female WT and APP/PS1 mice and there was no genotype or sex dependency in this measure (Suppl. Fig. 6A–J).

## 4 Discussion

Given the role of systemic inflammation in AD pathogenesis and known sex differences in the risk of AD and immune responses, we tested whether the behavioural and hippocampal metabolic responses to a systemic challenge with LPS would be exacerbated in young APP/PS1 female in the early hours post-inoculation. Here, we first show that the hippocampal metabolic signature of APP/PS1 mice, in the absence of immune stimulation, did not clearly differ from that of WT mice, at this early pathological stage, revealing only subtle differences, but predominantly in females. This is consistent with our previous study in males (Maroof et al., 2014). Differences in hippocampal metabolism have been previously reported in this model at 6 months of age, and in comparison to C57BL/6j mice rather than WT littermates, but without testing for potential sex differences although both males and females were included in that study (Gonzalez-Dominguez et al., 2014). Second, we also show in the absence of immune stimulation, a sexual dimorphism in the hippocampal metabolic profile of 4.5-month-old mice, particularly affecting methionine and pyruvate metabolism, but independent of the genotype. The major finding, however, was that 4 h after onset of systemic inflammation, several aspects of the LPS response were also sex-dependent. Importantly, we found at this time point that males and females exhibited comparable behavioural responses to LPS, regardless of the genotype, but the temperature change was greater in males and the cytokine response, particularly the secretion of IL-10, was greater in females. Metabolic data indicated that LPS induced a comparable activation of both the serotonin and nicotinamide pathways of tryptophan metabolism in the hippocampus of WT and APP/PS1 mice of both sexes, with hippocampal levels of the toxic nicotinamide metabolite 2PY being positively associated with the severity of the sickness response. And while all LPS-treated mice exhibited a downregulation in methionine levels, reversible oxidation and salvage, sex-differences were observed in the response of the recycling and transsulfuration pathways of methionine metabolism. Males also exhibited a downregulation of pyruvate metabolism after LPS, exacerbated in APP/PS1 males, while females showed downregulation of methylglyoxal metabolism.

### 4.1 Sex differences in hippocampal metabolism in the absence of immune stimulation

Spontaneous alternation was preserved in APP/PS1 mice regardless of sex, in agreement with our previous findings in both males and females at the same pathological stage (Bonardi et al., 2011, Maroof et al., 2014). In the present study, however, we observed an overall reduction in spontaneous alternation performance in females, suggesting a lower spatial working memory ability as was also previously reported in C57BL/6j mice (Tucker et al., 2016), the genetic background of our APP/PS1 mice. Males are indeed generally found to outperform females for spatial working memory, and this has been related to differences in hippocampal morphology and function (Koss and Frick, 2017). Accordingly, we have also shown that the hippocampal metabolic profile of females differed from that of males for 41 metabolites, but similarly in WT and APP/PS1 mice.

The most significant changes affected the metabolism of methionine, an essential amino acid for protein synthesis and epigenetic regulation in the brain, and key regulator of antioxidant protection and immune responses at physiological levels (Martinez et al., 2017, McGowan et al., 2008). Sex differences were seen in the salvage and transsulfuration pathways of methionine metabolism, with robustly downregulated levels of 5′-methylthioadenosine and hypotaurine, respectively. Methionine levels are in part regulated by the salvage pathway which recycles 5′-methylthioadenosine back into methionine (Albers, 2009). Reduced levels of 5′-methylthioadenosine in females were not associated with altered methionine levels, suggesting that they could be maintained *via* an alternative biosynthetic route and/or through reduced catabolism. The latter hypothesis is supported by the associated reduced levels of l-cystathionine and hypotaurine in females, two key intermediates in the synthesis of taurine from the methionine derivative homocysteine, during their degradation *via* the transsulfuration pathway (Stipanuk and Ueki, 2011). While methionine levels were preserved in females, the specific changes observed in downstream metabolites in females may predispose them to immune dysfunction and cell damage. 5′-Methylthioadenosine, is indeed now seen as a key regulator of immune responses to inflammation and systemic infections (Albers, 2009, Wang et al., 2017), proven to mediate protection against LPS-induced inflammation in vitro (Hevia et al., 2004), but also to inhibit inflammation and reduce brain damage in animal models of neuroinflammation (Moreno et al., 2006). Furthermore, hypotaurine which has well established antioxidant properties (Fontana et al., 2004), was also found to effectively suppress inflammatory and neuropathic pain (Hara et al., 2012). Taurine and hypotaurine are present in elevated levels in the brain of the long-lived Snell Dwarf mouse (Vitvitsky et al., 2013), which exhibits reduced oxidative damage to the brain (Brown-Borg, 2006) and hypothalamic inflammation (Sadagurski et al., 2015). Taurine also plays a protective role against age-related cognitive decline (El Idrissi, 2008), and therefore, the downregulated transsulfuration pathway of methionine metabolism could, in part, contribute to the lower spatial working memory performance seen in females.

Increased abundance in members of the glycolytic metabolic pathway, which provides energy for cellular metabolism in the form of pyruvate and ATP, was also observed in female hippocampi with particularly elevated levels of 3-phosphoglycerate and phosphoenolpyruvate in WT females. This was accompanied by unaltered pyruvate and ATP levels, but reduced levels of 2-phosphoglycolate, regardless of the genotype, possibly reflecting a metabolic shift towards enhanced regeneration of 3-phosphoglycerate from 2-phosphoglycolate, at the expense of glyoxylate metabolism, as suggested by the associated downregulation in 3-oxalomalate levels. Glycolate and glyoxylate metabolism have been linked to oxidative stress in peroxisomes (Schrader and Fahimi, 2006), essential organelles mediating biosynthetic and biodegradative reactions in a variety of cells (Terlecky et al., 2012). 2-phosphoglycolate is produced as a by-product of oxidative DNA damage (Segerer et al., 2016), and is also toxic for cells as a source of glycolate accumulation (Flugel et al., 2017). Conversion of glycolate into glyoxylate, a precursor of 3-oxalomate (Irace et al., 2007), is reduced in rats subjected to oxidative stress (Recalcati et al., 2003). This may lead to adverse effects as oxalomalate is known to prevent LPS-induced production of nitric oxide by activated macrophages (Irace et al., 2007). Peroxisome-associated oxidative stress is a mechanism thought to contribute to neurotoxicity, inflammation, cognitive dysfunction, and accelerated brain aging (Moruno-Manchon et al., 2018, Terlecky et al., 2012), but whether the metabolic changes seen in females reflect a pro- or anti-oxidant status will need to be addressed in further studies.

In PBS-treated mice, metabolic differences were not associated with significant sex differences in the number of astrocytes and microglia, or microglial soma size, a morphological activation marker. The area covered by microglia was, however, lower in the hippocampus of WT females, which could reflect reduced ramification per cell, as previously reported in females (Young et al., 2018). This reduction was not seen in APP/PS1 female mice, consistent with recent observations. Microglia of APP/PS1 mice was indeed found to develop ramifications in the presence of Aβ plaques, regardless of sex, but this response occurs earlier in females than in males (Frigerio et al., 2019), which can be related to the faster progression of cerebral amyloidosis consistently seen in females from this genotype (Frigerio et al., 2019, Li et al., 2016, Wang et al., 2003). APP/PS1 female mice also slightly differed from WT mice in their hippocampal metabolic profile, as well as with their reduced body weight and lack of habituation to repeated exposure to the Y-maze. But, while sex differences in brain metabolism seen in the ageing WT mouse brain have been hypothesised to contribute to the greater susceptibility of females to AD-like pathology (Zhao et al., 2016), our data do not support a link between differences in hippocampal metabolism and early-stage amyloidosis. The implication of the few metabolites found to be less abundant in the hippocampus of female APP/PS1 mice in Aβ plaques deposition is currently unknown. In contrast, global differences in hippocampal metabolism appeared to be emerging at 4.5 months of age between WT and APP/PS1 males, expected to develop the pathology at a slower rate than female, but in the absence of behavioural and/or physiological changes. Furthermore, the role of the metabolites found altered in female APP/PS1 mice in microglial morphology or function is also unknown, and we did not found any association between metabolite levels and microglial density and/or activation.

### 4.2 Metabolic effects of LPS independent of sex and disease status

4.5-month-old WT and APP/PS1 mice, regardless of their sex, exhibited a robust behavioural suppression 4 h after inoculation with a low systemic LPS dose of 100ug/kg, without concomitant changes in the number and morphology of glial cells in the hippocampus. This is consistent with findings in 4-month-old C57BL/6J females (Hart et al., 2012), but the discrepancy with our previous data showing enlargement of microglial soma in WT, but not APP/PS1 males at the same time point (Pardon et al., 2016), could be due to the exacerbating impact of anaesthesia (Ye et al., 2013), as previously discussed (Pardon et al., 2016). Indeed, morphological activation of microglia would generally occur 6 h after systemic administration of higher doses of LPS in the healthy brain (Hoogland et al., 2015), but within 4 h in the primed hippocampus (Murray et al., 2011). Thus, the fact that we did not find differences in microglial number and morphology between sexes and genotypes is consistent with the lack of microglial response at 4 h post-LPS, and published reports showing that changes in microglial phenotypes, occurring with the progression of amyloidosis in this APP/PS1 model, manifest after the age of 5 months (Martin et al., 2017). A subset of microglia, however, shows signs of activated morphology and phenotype prior to that, when clustering around Aβ plaques (Martin et al., 2017, Ruan et al., 2009), but our data show that both are rare in 4.5-month-old APP/PS1 mice.

As consistently reported with a range of systemic LPS doses, hippocampal tryptophan levels rapidly increased in the hippocampus of LPS-treated mice, independently of sex and disease status. This was associated with elevated levels of degradation products of both the serotonin and nicotinamide pathways of tryptophan metabolism, suggesting their co-activation, as seen previously (Guo et al., 2016, O'Connor et al., 2009, Parrott et al., 2016). A shift in the balance of brain tryptophan metabolism towards the kynurenine pathway is thought to be a major mediator of pro-inflammatory changes following systemic inflammation, and to ultimately cause serotonin deficiency (Kim and Jeon, 2018). Although serotonin levels were not measured here, as this would require the optimisation of a single LC/MS method specifically designed to address behavioural and structural differences between tryptophan metabolites and related monoamines (Fuertig et al., 2016), increased levels of its degradation product 5-HIAA could instead suggest an increase in serotonin turnover. Several studies have shown that elevated hippocampal 5-HIAA levels occurring in the first 24 h after inoculation with systemic LPS were associated with stable serotonin levels (Pitychoutis et al., 2009, Sens et al., 2017), suggesting an increased rate of serotonin synthesis (Brodie et al., 1966). Transient region-specific increases in the activity of enzymes involved in serotonin synthesis were indeed seen 2 h after systemic LPS in the frontal cortex and midbrain of rats challenged with the same 100ug/kg dose, but returning to baseline levels by the 6th hour post-inoculation (Nolan et al., 2000). In agreement with this, a time course microdialysis study showed that systemic LPS-induced a gradual increase in extracellular hippocampal tryptophan and 5-HIAA levels over 8 h, which was associated with a transient increase in serotonin levels, peaking 3–4 h after administration (Guo et al., 2016). Interestingly, the subsequent decline towards baseline serotonin levels was associated with a downregulation of the serotonin/tryptophan ratio and concomitant upregulation of the kynurenine/tryptophan ratio, indicating a metabolic shift towards kynurenine metabolism (Guo et al., 2016). Increased 2PY levels, reflecting the amount of nicotinamide biosynthesized from tryptophan (Shibata and Matsuo, 1990), have been linked to activation of tryptophan metabolism through the kynurenine pathway and associated with systemic inflammation in malnutrition (Guerrant et al., 2016, Mayneris-Perxachs et al., 2016). We can, therefore, hypothesise that the elevated hippocampal 2PY levels we saw 4 h after LPS administration would reflect an activation of the tryptophan-nicotinamide pathway, but whether or not its association with elevated hippocampal 5-HIAA levels predict a metabolic shift towards kynurenine metabolism will need to be determined in future studies, by measuring the levels of serotonin and key intermediates of the kynurenine pathway at later points. This is particularly important because activation of the serotonin pathway of tryptophan metabolism is protective to the brain, whereas activation of the kynurenine pathway leads to detrimental effects, the former being anti-inflammatory (Dominguez-Soto et al., 2017) and latter pro-inflammatory (Davis and Liu, 2015) and a major driver of LPS-induced sickness (O'Connor et al., 2009). This is also consistent with our observed association between 2PY levels and the severity of the behavioural and temperature response to LPS. The time course of changes in tryptophan metabolic pathways also has implication for our understanding of the mechanisms underlying the precipitating influence of systemic infection and inflammation in AD. Recent findings indeed suggest that reduced serotonin neurotransmission contributes to the development of early cognitive symptoms of AD (Smith et al., 2017), while upregulation of key components of the kynurenine pathway are associated with Aβ plaques and neurofibrillary tangles in the brain of AD patients (Wu et al., 2013).

We also showed, for the first time, that systemic LPS rapidly inhibited the synthesis and metabolism of methionine in the hippocampus, illustrated by the lowering of both l-methionine and its downstream metabolites. Regardless of sex, LPS altered methionine reversible oxidation and salvage. Methionine reversible oxidation, the process whereby methionine is oxidized into methionine sulfoxide, which is then reduced back to methionine, is thought to play a key regulatory role in mediating activity-dependent plastic changes in cellular excitability (Hoshi and Heinemann, 2001). The reduction in the levels of the methionine oxidation product l-methionine S-oxide by LPS could be seen as a protective response since reversible methionine oxidation becomes impaired in ageing and related diseases, leading to the accumulation of toxic methionine oxidation products, oxidative damage to cells (Stadtman et al., 2005) and Aβ accumulation (Moskovitz et al., 2016). Methionine sulfoxide reductase, the antioxidant enzyme which reduces methionine sulfoxide back to methionine, is also known to alleviate LPS-induced inflammation in microglia (Fan et al., 2015). However, we found here that this was associated with reduced abundance of l-methionine, suggesting that the lack of l-methionine availability, rather than enhanced reversible oxidation, caused the lowering of l-methionine S-oxide levels. The salvage pathway plays a critical role in maintaining optimal methionine levels as proven by the ability of 5′-methylthioadenosine to replenish the cellular methionine pool within 24 h of methionine deprivation (Shiraki et al., 2014). Reduced levels of both S-adenosyl-l-methionine and 5′-methylthioadenosine, as we saw here associated with methionine deficiency 4 h after LPS administration, are thought to be early events reflecting the activation of the salvage pathway for the rescue of methionine levels, but this could only confirmed by looking at later time points (Shiraki et al., 2014). A failure of this mechanism would lead to detrimental effects, by compromising cell differentiation, growth and survival (Shiraki et al., 2014) as well as protein synthesis and epigenetic reactions. The methionine salvage pathway indeed recycles the sulphur of 5′-methylthioadenosine back into methionine, which is critically needed for protein synthesis (Kabil et al., 2014). S-adenosyl-l-methionine, whose levels were particularly decreased by LPS in female mice, is a precursor for this reaction but also a methyl donor for epigenetic reactions and key regulator of metabolism, proliferation, and apoptosis (Albers, 2009). Persistent downregulation of the salvage pathway could also be damaging due to the anti-inflammatory properties of both S-adenosyl-l-methionine and 5′-methylthioadenosine (Ge et al., 2018, Hevia et al., 2004, Moreno et al., 2006, Pfalzer et al., 2014), whose levels were also found reduced in the AD brain (Morrison et al., 1996). However, while methionine deficiency, if persistent, can impair multiple aspects of cell function, and ultimately cell viability (Shiraki et al., 2014), and as such contribute to the development of AD, its excess is also neurotoxic leading to inflammation and exacerbation of behavioural and neurological markers of AD (Tapia-Rojas et al., 2015), perhaps questioning the functional significance of our findings. Reduced availability and metabolism of methionine is indeed thought to be the main driver of lifespan and healthspan enhancement by dietary restriction through improved lipid metabolism as well as reduced oxidative stress, inflammation and susceptibility to immune and central nervous disorders (Martinez et al., 2017, Orgeron et al., 2014). Methionine metabolism is indeed reduced in the long-lived naked-mole rat (Lewis et al., 2018) but it is enhanced in the long-lived Ames dwarf mouse (Uthus and Brown-Borg, 2003), and both show activation of anti-inflammatory pathways (Cheng et al., 2017, Dhahbi et al., 2007).

Thus, whether our observed rapid downregulation of methionine metabolism by LPS constitutes a beneficial or detrimental response in the brain cannot be fully answered in this study and our data also suggest that this may be, in part, sex-dependent. Indeed, O-succinyl-l-homoserine contributes to the synthesis of both homocysteine and l-methionine (Flavin and Slaughter, 1967), and in the present study, it was less abundant in LPS-treated females. This is particularly relevant to the link between immune responses and AD progression, since homocysteine can trigger neuroinflammation and microglial activation (Chen et al., 2017) and its levels are positively associated with the risk of dementia (Smith et al., 2018), therefore suggesting a protective downregulation of methionine metabolism in females. In contrast, LPS-treated males instead presented with increased levels of glutathione disulfide, a toxic oxidized form of glutathione and an end product of methionine metabolism, whose circulating levels were found to be elevated in inflammatory conditions (Ikegami et al., 1994), and reduced in the long-lived naked-mole rat (Lewis et al., 2018), suggesting a more damaging downregulation of methionine metabolism.

### 4.3 Sexual dimorphism in the hippocampal metabolic response to LPS

Changes in body temperature, pyruvate and methylglyoxal metabolism 4 h after onset of systemic inflammation were clearly sex-dependent. Thermoregulatory responses to LPS in rodents are made of one to three phases of hyperthermia and/or hypothermia, although fever is less likely to occur in mice than in rats (Blanque et al., 1996, Romanovsky et al., 2005). LPS-induced hypothermia is seen as a thermoregulatory “failure”, thought to reflect the inability of the brain to regulate body temperature in shock (Romanovsky et al., 2005). It is also an indicator of the severity of LPS responses, as it was found to be dose-dependent and more pronounced in mouse strains susceptible to this endotoxin (Blanque et al., 1996). Consistent with our findings, LPS-induced hypothermia is generally found to be more severe in male than female mice (Cai et al., 2016, Card et al., 2006), suggesting that they experience more severe acute effects of LPS. Hypothermia is thought to be in part induced in response to changes in the brain as it did not appear to be related to variations in circulating levels of IL-1β, IL-6 or IL-10 (Blanque et al., 1996, Skelly et al., 2013). Our data showing that LPS-induced hypothermia is correlated with increased 2PY levels, thought to reflect an activation of the tryptophan-nicotinamide pathway, are consistent with previous reports showing that sickness responses to LPS are in part mediated by activation of the kynurenine pathway (O'Connor et al., 2009) and the ability of kynurenine and its metabolites to potentiate drug-induced hypothermic responses (Lapin, 2003). Here, hypothermia was sex-dependent and this was also associated with a reduction in hippocampal pyruvate metabolism particularly affecting APP/PS1 males. A possible link cannot be ruled out as hypothermia was found to cause a progressive decrease in cerebral pyruvate contents in the rat (Nilsson et al., 1975), although central administration of pyruvate did not significantly alter body temperature (Soto et al., 2018). Pyruvate is an intermediate energy metabolite of glucose with potent anti-oxidant and anti-inflammatory actions (Das, 2006). Sex differences in pyruvate metabolism have been associated with a higher mitochondrial respiration rate and reduced oxidative stress in females (Gaignard et al., 2015), as well as protection against oxidative damage induced by excitotoxic injury whereby the ratio of lipid peroxidation markers over pyruvate increased in males but decreased in females in response to an ischemic insult (Wagner et al., 2004). Evidence of anti-inflammatory effects of pyruvate in the brain include the demonstration that treatment with its ethyl pyruvate derivatives exerts robust neuroprotective effects, in both the post-ischemic brain and LPS-treated animals, by alleviating microglial activation and neutrophil infiltrations *in vivo*, and inhibiting LPS-induced pro-inflammatory changes in these cells *in vitro* (Lee et al., 2017, Lee et al., 2013). In addition, dietary supplementation with pyruvate was found to improve spatial memory impairments and brain energy metabolism in males of the same APP/PS1 mouse line as used here (Koivisto et al., 2016), as well as in male and female 3xTg-AD mice, while also reducing oxidative stress, albeit without effects on Aβ and tau pathology (Isopi et al., 2015). Although systemic inflammation has been linked to cognitive decline and AD progression in both males and females (Holmes et al., 2009, Trollor et al., 2010), sex-specific differences were found in the association between pro-inflammatory mediators and cognitive function in mild cognitively impaired patients (Trollor et al., 2010). Thus, the present finding that male APP/PS1 mice were more susceptible to downregulation of pyruvate metabolism in the early hours post-inoculation with LPS could constitute a male-specific mechanism underlying exacerbation of cognitive and neurodegenerative changes after systemic inflammation.

In contrast, females displayed a downregulation of methylglyoxal metabolism illustrated by reduced levels of its upstream regulators, with the exception of glycolysis, and of its reduction product d-lactate. Methylglyoxal is a cytotoxic and pro-inflammatory glycotoxin whose deleterious effects are due to its role as a major precursor of advanced glycation end-products, and have been associated with several pathologies including diabetes, ageing and neurodegenerative diseases (Allaman et al., 2015, Angeloni et al., 2014, Beeri et al., 2011). Cerebrospinal fluid as well as serum methylglyoxal concentrations are increased in AD patients (Beeri et al., 2011, Kuhla et al., 2005) and the latter has been found to be associated with cognitive decline regardless of sex (Beeri et al., 2011). Direct administration of methylglyoxal was also found to cause cognitive deficits in rats (Hansen et al., 2016) and its accumulation promotes inflammation (Vulesevic et al., 2016) as well as Aβ aggregation (Woltjer et al., 2003). Methylglyoxal is also produced by Aβ, contributing to cell death (Tajes et al., 2014), providing a link between inflammation and AD exacerbation. In this context, we can hypothesise that the inhibition of methylglyoxal metabolism seen in females in response to systemic inflammation would constitute a protective response that, if persistent, may limit inflammation-induced exacerbation of AD-like pathology. The mechanism behind a sexual dimorphism in this response, is however unknown. Females were found to be more susceptible to the acute toxicity of methylglyoxal (Peters et al., 1978), but the present study is the first show a sex-dependent response of this metabolic pathway to immune stimulation. Nevertheless, methylglyoxal metabolism has been implicated in obesity and diabetes (Matafome et al., 2013), the risk of which is exacerbated by systemic inflammation (Esser et al., 2014), and females have been found less susceptible to diet-induced obesity and its metabolic and pro-inflammatory consequences (Dorfman et al., 2017). Our finding is, however, consistent with the elevated levels of the anti-inflammatory cytokine IL-10 that we found in LPS-treated females, but not males, at the 4 h time point. Indeed, methylglyoxal was found to particularly inhibit the secretion of IL-10 and TNFα by myeloid cells (Price et al., 2010), whereas IL-10 can suppress pro-inflammatory and toxic effects of methylglyoxal (Onishi et al., 2015).

## 5 Conclusions

Taken together, our data indicate that while hippocampal metabolism in females, compared to males, may reflect a shift towards a pro-inflammatory and pro-oxidant signature, and while subtle metabolic differences in APP/PS1 mice compared to their WT littermates were only seen in females, in the early hours following inoculation with LPS, the physiological and metabolic responses of males are more pronounced, regardless of the genotype. This is consistent with recent findings in a model of traumatic brain injury, whereby males exhibited a more aggressive neuroinflammatory profile than female mice during the acute and subacute phases post-injury (Villapol et al., 2017), and also with the recently established sexual dimorphism in microglia. The molecular signature of male microglia was indeed found to be skewed towards pro-inflammatory activation and that of females found to be neuroprotective, expressing proteins related to the inhibition of inflammatory responses and promotion of repair mechanisms (Guneykaya et al., 2018, Villa et al., 2018a, Villa et al., 2018b). Metabolic changes in the hippocampus occurred in the present study before morphological signs of microglial activation could be detected, affecting pathways known to modulate microglial activation. This provides insights into how systemic LPS, whose brain penetration is poor at low dose and in the absence of blood brain barrier dysfunction (Banks and Robinson, 2010, Varatharaj and Galea, 2017), can trigger rapid inflammatory responses in the brain. Importantly, we have found here that some of the metabolic changes are sex-specific, highlighting the importance of taking gender into consideration when studying susceptibility to inflammatory conditions and AD exacerbation. Moreover, this study is the first to show an association between onset of systemic inflammation and downregulation of hippocampal methionine metabolism, but the protective or detrimental nature of this change needs to be determined in future studies. No major differences in the response to LPS were seen here between WT and APP/PS1 mice, which can be due to the early pathological stages under investigation, since differences in microglial activation are subtle before the age of 4 months (Martin et al., 2017, Ruan et al., 2009), and/or the early time point, as the resolution of neuroinflammation is an active process particularly impaired in neurodegenerative diseases (Schwartz and Baruch, 2014). However, some of the metabolic changes that we saw here in the hippocampus, a brain area critically affected by AD, 4 h after induction of systemic inflammation with LPS, are relevant to AD pathogenesis, particularly the activation of the tryptophan-nicotinamide pathway, reduced methionine availability and salvage as well as the reduction in pyruvate metabolism in males. The extent to which a single infectious episode is sufficient to drive the progression of AD in susceptible individuals is not currently known. Looking at the persistence of these metabolic alterations in relation to the progression of behavioural and neurological hallmarks of AD may help answering into this question.
