# Supplementary material for: Sex-specific hippocampal metabolic signatures at the onset of systemic inflammation with lipopolysaccharide in the APPswe/PS1dE9 mouse model of Alzheimer’s disease
Source: Brain Behav Immun. 2020 Jan;83:87–111. doi: 10.1016/j.bbi.2019.09.019 (PMC6928588; doi:10.1016/j.bbi.2019.09.019)
Supplement: Supplementary data 1 [file mmc1.pdf]

Supplementary information

Sex-specific hippocampal metabolic signatures at the onset of systemic inflammation with lipopolysaccharide in the APPswe/PS1dE9 mouse model of Alzheimer's disease

Alessandra Agostini, Ding Yuchun, Bai Li, David A. Kendall and Marie-Christine Pardon

Supplementary Figure 1. Body weight, baseline and post-injection performance in the spontaneous alternation task

Supplementary Table 1. Results of the ANOVAs for behavioural and physiological variables.

Supplementary Table 2. Results of the ANOVAs for plasma cytokine levels

Supplementary metabolomics results

Supplementary Table 3. Potential function of discriminant metabolites in the brain and implication in sex differences in brain function, AD progression and immune status

References for supplementary metabolomics results

Supplementary Figure 2. Discriminant metabolites for genotype differences

Supplementary Figure 3. Discriminant metabolites for sex differences

Supplementary Figure 4. Discriminant metabolites for LPS effects

Supplementary Table 4. Results of the ANOVAs for glial measures

Supplementary Figure 5. Microglial soma size

Supplementary Figure 6. GFAP immunostaining results

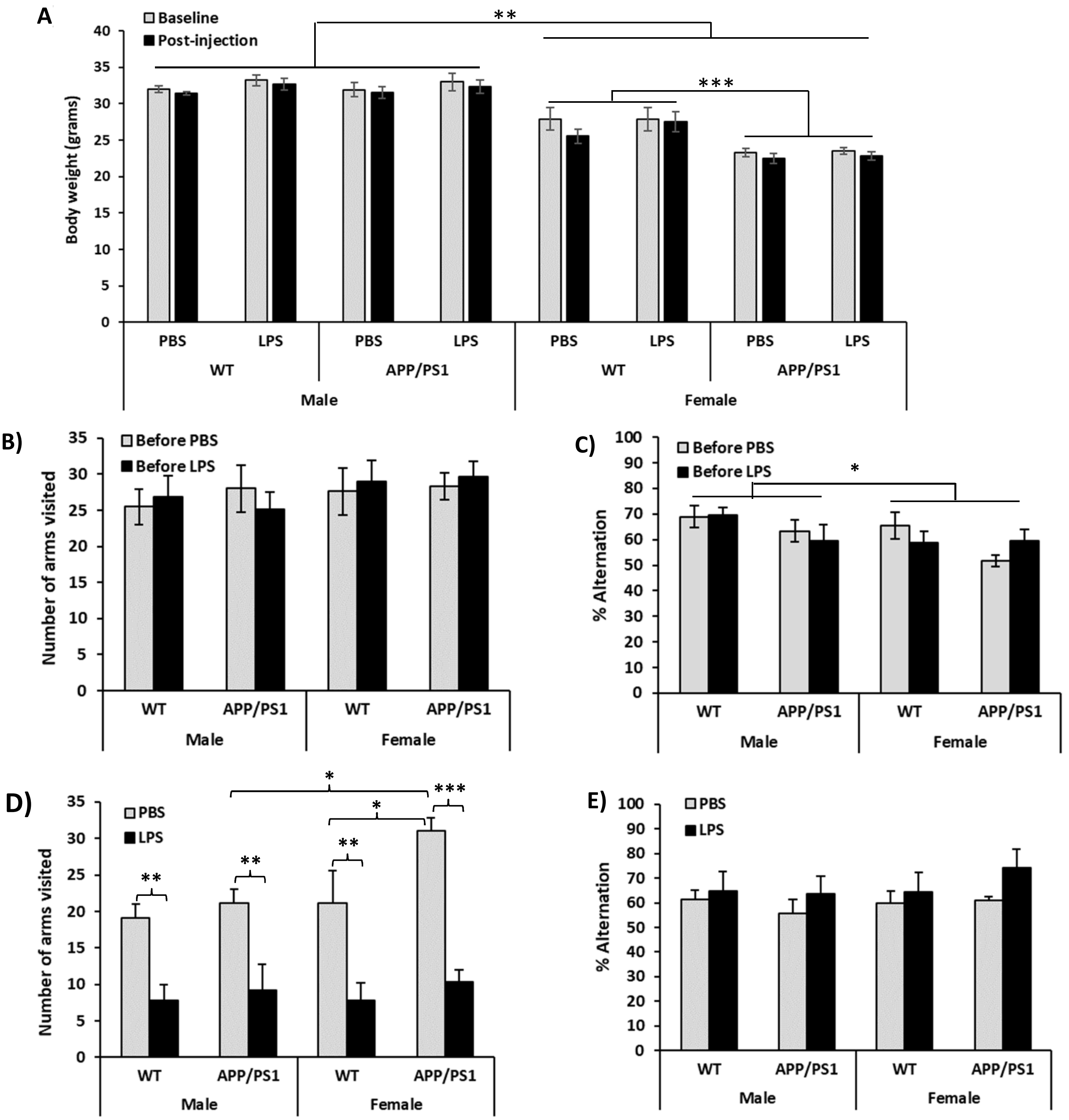

Supplementary Figure 1. Body mass (A) was recorded prior to and 4 hours after the PBS or LPS challenge. The two-way ANOVA with repeated measures revealed a significant Genotype x Sex interaction effect ($F_{(1,36)}$ = 12.22, p=0.0013). Females were overall lighter than males regardless of genotype ($F_{(1,36)}$ = 152.67, p<0.0001) but within females, the APP/PS1 mice were also lighter than their WT littermates (p<0.0001). None of the experimental groups showed significant weight loss at 4 hours following the LPS or PBS challenge. Before injection with PBS or LPS, none of the groups differed for the number of arm visits in the Y-maze (B), but females showed an overall reduction in spatial alternation performance (B; Sex effect: $F_{(1,35)}$ = 4.65, p=0.04). At 4 hours post injection, LPS-treated mice visited significantly less arms of the Y-maze than PBS-treated mice ($F_{(1,36)}$ = 65.53, p<0.0001) and but PBS-treated APP/PS1 mice were also hyperactive compared to PBS-treated APP/PS1 males and WT females (p=0.009 in both cases, C), but their spontaneous alternation performance (D) did not differ. Data are expressed as Means $\pm$ SEM. *, p<0.05; **, p<0.01; **, p<.0001

Supplementary table 1. Results of the two-way repeated measure ANOVAs on behavioral measures.

	**Body weight**	**Food Burrowing***	**Arm entries**	**Alternation rate**
**Covariate (arm entries)**				$F_{(1,31)}$=0.80 p=0.38
**Genotype**	$F_{(1,36)}$=14.4 **p=0.0006**	$F_{(1,36)}$=0.03 p=0.86	$F_{(1,36)}$=2.12 p=0.15	$F_{(1,36)}$=0.88 p=0.36
**Sex**	$F_{(1,36)}$=152.67 **p<0.0001**	$F_{(1,36)}$=2.30 p=0.14	$F_{(1,36)}$=3.17 p=0.08	$F_{(1,36)}$=0.11 p=0.74
**Treatment**	$F_{(1,36)}$=2.30 p=0.14	$F_{(1,36)}$=22.2 **p<0.0001**	$F_{(1,36)}$=20.66 **p<0.0001**	$F_{(1,36)}$=0.41 p=0.53
**Time**	$F_{(1,35)}$=9.37 **p=0.004**	$F_{(1,36)}$=15.92 **p=0.003**	$F_{(1,36)}$=145.25 **p<0.0001**	$F_{(1,31)}$=0.18 p=0.68
**Genotype X Sex**	$F_{(1,36)}$=12.22 **p=0.0013**	$F_{(1,36)}$=1.22 p=0.28	$F_{(1,36)}$=0.59 p=0.45	$F_{(1,36)}$=1.09 p=0.30
**Genotype X Treatment**	$F_{(1,36)}$=0.19 p=0.67	$F_{(1,36)}$=0.20 p=0.66	$F_{(1,36)}$=0.97 p=0.33	$F_{(1,36)}$=0.85 p=0.36
**Genotype X Time**	$F_{(1,35)}$=0.35 p=0.56	$F_{(1,36)}$=1.62 p=0.21	$F_{(1,36)}$=3.09 p=0.09	$F_{(1,31)}$=2.98 p=0.09
**Sex X Treatment**	$F_{(1,36)}$=0.17 p=0.68	$F_{(1,36)}$=0.37 p=0.55	$F_{(1,36)}$=0.28 p=0.60	$F_{(1,36)}$=0.18 p=0.67
**Sex X Time**	$F_{(1,35)}$=1.06 p=0.31	$F_{(1,36)}$=0.06 p=0.81	$F_{(1,36)}$=0.25 p=0.62	$F_{(1,31)}$=4.25 **p=0.048**
**Treatment X Time**	$F_{(1,35)}$=1.02 p=0.32	$F_{(1,36)}$=9.47 **p=0.004**	$F_{(1,36)}$=58.55 **p<0.0001**	$F_{(1,31)}$=0.65 p=0.43
**Genotype X Sex X Treatment**	$F_{(1,36)}$=0.04 p=0.84	$F_{(1,36)}$=3.13 p=0.08	$F_{(1,36)}$=0.04 p=0.84	$F_{(1,36)}$=0.97 p=0.33
**Genotype X Sex X Time**	$F_{(1,35)}$=0.32 p=0.57	$F_{(1,36)}$=0.46 p=0.50	$F_{(1,36)}$=1.20 p=0.28	$F_{(1,30)}$=0.73 p=0.40
**Genotype X Treatment X Time**	$F_{(1,35)}$=1.05 p=0.31	$F_{(1,36)}$=0.12 p=0.73	$F_{(1,36)}$=0.24 p=0.63	$F_{(1,30)}$=0.01 p=0.91
**Sex X Treatment X Time**	$F_{(1,35)}$=1.29 p=0.27	$F_{(1,36)}$=0.30 p=0.59	$F_{(1,36)}$=3.79 p=0.06	$F_{(1,30)}$=0.00 p=0.95
**Genotype X Sex X Treatment X Time**	$F_{(1,35)}$=0.59 p=0.45	$F_{(1,36)}$=0.09 p=0.77	$F_{(1,36)}$=1.94 p=0.17	$F_{(1,30)}$=0.78 p=0.38

*: Food burrowing data were rank-transformed prior to statistical analyses.

Supplementary table 2. Results of the two-way repeated measure ANOVAs on plasma cytokine levels. Data were rank-transformed prior to statistical analyses.

	**IL-6**	**TNF-α**	**INF-γ**	**IL-1β**	**IL-10**
**Genotype**	$F_{(1,30)}$=0.43 p=0.51	$F_{(1,30)}$=0.21 p=0.65	$F_{(1,30)}$=0.59 p=0.45	$F_{(1,30)}$=0.05 p=0.83	$F_{(1,30)}$=0.88 p=0.35
**Sex**	$F_{(1,30)}$=0.76 p=0.39	$F_{(1,30)}$=1.04 p=0.31	$F_{(1,30)}$=0.41 p=0.52	$F_{(1,30)}$=0.93 p=0.34	$F_{(1,30)}$=5.07 **p=0.03**
**Treatment**	$F_{(1,30)}$=116.02 **p<0.0001**	$F_{(1,30)}$=7.82 **p=0.0089**	$F_{(1,30)}$=2.7 p=0.11	$F_{(1,30)}$=0.17 p=0.68	$F_{(1,30)}$=23.49 **p<0.0001**
**Genotype X Sex**	$F_{(1,30)}$=0.03 p=0.87	$F_{(1,30)}$=0.09 p=0.77	$F_{(1,30)}$=0.37 p=0.55	$F_{(1,30)}$=0.90 p=0.35	$F_{(1,30)}$=0.00 p=0.95
**Genotype X Treatment**	$F_{(1,30)}$=4.01 p=0.054	$F_{(1,30)}$=0.48 p=0.49	$F_{(1,30)}$=1.12 p=0.30	$F_{(1,30)}$=0.40 p=0.53	$F_{(1,30)}$=0.18 p=0.67
**Sex X Treatment**	$F_{(1,30)}$=3.13 p=0.09	$F_{(1,30)}$=2.15 p=0.15	$F_{(1,30)}$=0.98 p=0.33	$F_{(1,30)}$=0.77 p=0.38	$F_{(1,30)}$=4.54 **p=0.04**
**Genotype X Sex X Treatment**	$F_{(1,30)}$=3.00 p=0.09	$F_{(1,30)}$=2.69 p=0.11	$F_{(1,30)}$=0.90 p=0.35	$F_{(1,30)}$=2.26 p=0.14	$F_{(1,30)}$=1.68 p=0.20

Supplementary metabolomics results

Lack of major metabolic perturbations in the hippocampus of 4.5-month-old APP/PS1 mice

5 of the 98 selected metabolites were found to significantly discriminate between PBS-treated WT and APP/PS1 mice (Table1), albeit predominantly in females. Levels of L-beta-aspartyl-L-glutamic acid, which belongs to the family of N-acyl-alpha amino acids and derivatives which are known for their anti-inflammatory action (1), were particularly reduced in female APP/PS1 mice (Suppl. Fig. 2A), but its function and implication in AD pathology is, to the best of our knowledge, unknown. 1-Methyladenosine, an oxidized nucleoside known to be immunosuppressive on macrophage function (2) and found in elevated levels in the urine of patients with mild-to-moderate AD (3), was more abundant in the hippocampus of female APP/PS1 mice compared to their WT female littermates (Suppl. Fig. 2B).

Significant Genotype X Treatment interactions were also found for N-acetyl-(L)-arginine ($F_{(1,34)}$=12.07, p=0.001), whose levels were significantly lower in PBS-treated APP/PS1 females compared to PBS-treated WT females (Suppl. Fig. 2C), and for the hydrophobic tetrapeptide Asp-Phe-Thr-Thr ($F_{(1,34)}$=5.40, p=0.03), whose levels were significantly increased in PBS-treated APP/PS1 males compared to their PBS-treated counterparts (Suppl. Fig. 2D). Their function and potential roles in AD pathology are also, to the best of our knowledge, unknown.

Sex differences in the hippocampal metabolic profile are independent of the APP/PS1 genotype.

Forty-one metabolites with sex differences were identified revealing major changes in amino acids, carbohydrate metabolism and fatty acyls (Table 1). A few metabolites from other chemical classes and many unknown metabolites were also found in different levels between PBS-treated males and females (Table 1). Metabolic differences in the methionine and pyruvate metabolic pathways are described in the main manuscript and illustrated Fig. 5 and 6, respectively. Changes in other metabolites with previously associated with differences immune function are described below. Their potential role in brain function or implication in AD progression is presented in Suppl. Table 3.

These included reduced levels of (3R)-beta leucine (Suppl. Fig. 3), a degradation product of the anti-inflammatory amino acid L-Leucine (4), D-alanyl-D-alanine (Suppl. Fig. 3B), an anti-inflammatory antibiotic-binding protein (5), and N(pi)-methyl-L-histidine (Suppl. Fig. 3C), a metabolic product of the amino acid histidine known to be negatively associated with inflammation in obese women (6). Females also presented with an increased abundance of N-Succinyl-L-glutamate 5-semialdehyde (Suppl. Fig. 3D), a metabolite found to be elevated in the plasma of lung cancer patients harbouring a mutation in the epidermal growth factor receptor (7) that also exacerbate their pro-inflammatory status (8).

Lysine, whose dietary restriction was found to trigger pro-inflammatory changes (9), indicated the major anti-inflammatory pathway upregulated in females as seen by elevated levels in three metabolites involved in lysine biosynthesis :N6-(L-1,3-Dicarboxypropyl)-L-lysine, N2-Acetyl-L-aminoadipate and L-2-Aminoadipate (Suppl. Fig. 3E-G), and reduced levels of L-pipecolate (Suppl. Fig. 3H), a degradation product of L-lysine whose urine levels are positively associated with low grade inflammation (10). Differences in amino acid metabolism also indicative of anti-inflammatory effects in females included increased abundance of pantothenate (vitamin B5; Suppl. Fig. 3I), whose dietary intake was found to alleviate chronic low grade inflammation (11) and norepinephrinesulfate (Suppl. Fig. 3O), a metabolite of the anti-inflammatory neurotransmitter norepinephrine (12), as well as reduced levels of (Z)-4-

Hydroxyphenylacetaldehyde-oxime (Suppl. Fig. 3J), an enzyme involved in tyrosine metabolism found in increased levels in inflammatory bowel disease (13), and homoarginine (Suppl. Fig. 3K), known to be negatively associated with pro-inflammatory changes (14).

Changes in carbohydrate metabolism and fatty acyls seen in females were indicative of a pro-inflammatory status. Furthermore, isocitrate, a substrate of the tricarboxylic acid (TCA) cycle found to exert anti-inflammatory effects in a rat model of mild anemia of inflammation (15) and itaconate, a potent anti-inflammatory TCA derivative found in immune cells (16), were also less abundant in the female hippocampus (Suppl. Fig. 3M&N, respectively). Hippocampal concentrations of formyl 3-hydroxy-butanoate, a fatty ester, were also significantly lower in females (Suppl. Fig. 3L) and fatty esters are thought to be anti-inflammatory (17).

Metabolites differentially expressed in PBS- and LPS-treated mice regardless of sex and genotype.

Thirty six metabolites were altered to similar extents by LPS in all experimental groups (Table 1). The most significant changes were those affecting tryptophan and methionine metabolism, as described in the main manuscript and represented Figs. 4&5, respectively. Changes to other metabolites associated with immune status are described below and represented Suppl. Fig. 4, and the potential association of these metabolites in brain function, sex differences and/or AD progression is described in Suppl. Table 3.

These metabolic differences included increased levels of thymidine and thymine (Suppl. Fig. 4A&B), two derivatives of the anti-inflammatory nucleotide pyrimidine (18) as well as reduced levels of the inflammation signalling molecule adenosine triphosphate (ATP) (19), particularly in WT mice (Suppl. Fig. 4C) and of succinate (Suppl. Fig. 4D), a pro-inflammatory intermediate of the TCA cycle which plays a crucial role in ATP generation (20).

Pro-inflammatory metabolic changes included increased levels of N(pi)-methyl-L-histidine (Suppl. Fig. 4C), an histidine derivative positively associated with levels of pro-inflammatory markers (21), urate (Suppl. Fig. 4E) known to cause cognitive deficits through enhancing hippocampal inflammation (22), and of its downstream metabolite urea-1-carboxylate (Suppl. Fig. 4F). LPS-treated mice also had elevated hippocampal levels of L-phenylalanine (Suppl. Fig. 4G), whose circulating levels are increased in elderly people with chronic low grade inflammation (23) and prostaglandin-H2-ethanolamide (PGH2-EA) (Suppl. Fig. 4H), a precursor of prostaglandin E2 (PGE_2), which plays a major role in acute inflammation and transition to chronic inflammation (24), and is a key mediator of LPS-induced sickness (25).

Metabolites sex-dependently affected by LPS

Forty-six metabolites showed sex-dependent effects of LPS, of which twenty-one were selectively altered in males, twenty were selectively altered in females, and five showed opposite effects in the two sexes (Table 1). These changes particularly affected two metabolic pathways: pyruvate (Fig. 6) and methylglyoxal (Fig. 8). Changes in other metabolites known to be associated with immunomodulation are reported below and illustrated Suppl. Fig. 4.

Metabolic effects of LPS in males

Levels of methylimidazoleacetic acid (Sex X Treatment: $F_{(1,34)}$=13.22, p=0.0009, Suppl. Fig. 4J), a metabolite of the pro-inflammatory mediator histamine (26) were increased by LPS in APP/PS1 males.

Other metabolic changes found in LPS-treated males include increased levels of norepinephrinesulfate (Sex X Treatment: $F_{(1,34)}=6.64$, $p=0.01$, Suppl. Fig. 3O), a metabolite of the anti-inflammatory neurotransmitter norepinephrine (12), choline (Sex X Treatment: $F_{(1,34)}=19.89$, $p<0.0001$, Suppl. Fig. 4K), a precursor of acetylcholine and agonist of α7 nicotinic receptors expressed in neurons and macrophages with established anti-inflammatory effects (27, 28), found to dose-dependently inhibit LPS-induced TNFa production by macrophages (29), L-rhamnose (Sex X Treatment: $F_{(1,34)}=7.25$, $p=0.01$, Suppl. Fig. 4L), previously shown able to inhibit pro-inflammatory cytokines production (30), and hypoxantine (Sex X Treatment: $F_{(1,34)}=10.06$, $p=0.003$, Suppl. Fig. 4M), whose levels are negatively correlated with the severity of mucosal inflammation (31).

Metabolic effects of LPS in females

6-Phospho-D-gluconate (Suppl. Fig. 4N), an intermediate of the pentose phosphate pathway known to trigger pro-inflammatory cytokines secretion in LPS-activated macrophages (32), was less abundant in LPS-treated females

Pro-inflammatory metabolic changes found in LPS-treated females include reduced levels of N2-Succinyl-L-ornithine (Sex X Treatment: $F_{(1,34)}=6.25$, $p=0.02$, Suppl. Fig. 4N), a degradation product of the anti-inflammatory amino acid arginine (33), and, in WT LPS-treated females, and homoarginine (Suppl. Fig. 3K), known to be negatively associated with pro-inflammatory changes (14).

Metabolites showing opposite pattern in LPS-treated males and females

Two of the five metabolites that showed opposite effects of LPS in males and females, S-Adenosy-L-homocysteine Fig. 4E) and N-Succinyl-L-glutamate 5-semialdehyde (Suppl. Fig. 3D), have been reported to be associated with increased inflammation. They both were found more abundant in male hippocampi but less abundant in female hippocampi 4 hours after LPS administration.

Suppl. Table 3. Physiological role of metabolites from known metabolic pathways differently expressed between PBS-treated WT and APP/PS1 mice, PBS-treated males and females and in response to LPS.

Putative metabolite	Metabolic Pathway	Physiological role in the brain	Implication in sex differences in brain function	Implication in Alzheimer's disease (AD)	Implication in immune status
Amino acid metabolism					
(3R)-beta-Leucine	Valine, leucine and isoleucine degradation	Degradation product of L-Leucine which is produced by muscle protein catabolism and serves as a donor for brain glutamate synthesis by astrocytes and cerebral protein synthesis (34).	Not known	Increased serum levels of l-leucine in AD patients and in the 3xTg mouse model of AD (35). L-leucine up-regulates tau phosphorylation in 3xTg mice (35).	L- leucine reduces inflammation and increases repair after muscle injury in rats (4).
Choline	Glycine, serine and threonine metabolism	Precursor for the cerebral synthesis of acetylcholine, a neurotransmitter essential for cognitive function, and phospholipid phosphatidylcholine, a major constituent of biological membranes in neurons and glial cells (36, 37).	Higher choline concentrations in the hippocampus of cognitively intact elderly females (38).	Loss of cholinergic function in AD is associated with memory decline (39). Dietary intake of choline improves cognitive function in AD patients and mouse models (37).	Agonist of α7 nicotinic receptors expressed on macrophages, supresses LPS-induced TNFa production by macrophages (29).
L-cystathionine	Glycine, serine and threonine metabolism<br>Methionine metabolism	Intermediate in the transsulfuration pathway which decreases neurotoxic homocysteine concentrations (40)<br>Mediates the conversion of homocysteine into cysteine (Fig. 5).	Not known	Increased levels in the temporal cortex of post-mortem AD brains (41).	Inhibits the expression of the pro-inflammatory cytokine MCP-1 in macrophages *in vitro* (42).
L-methionine	Methionine metabolism	Key role in epigenetic regulation in the brain through conversion into homocysteine via S-adenosyl-L-methionine (43).	No differences in mouse brain concentrations (44).	Decreased levels in the temporal cortex of post-mortem AD brains (41). Elevated CSF levels in MCI and AD (45). Excess dietary methionine induces cognitive and neurological hallmarks of AD in mice (46).	Excess dietary methionine induces astrocyte and microglia activation in the hippocampus (46). Induces pro-inflammatory activation in macrophages in vitro (47).
L-methionine S-oxide	Methionine metabolism	Toxic oxidation product of methionine (48).	Not known	Increased production triggers Aβ aggregation (49).	Inhibition reduces TNFa and IL1β secretion in LPS-stimulated microglia (50).
S-adenosyl-L-homocysteine	Methionine metabolism	Biosynthetic precursor of homocysteine (Fig. 5) which is neurotoxic and pro-inflammatory in microglia (51). Formed by demethylation of S-adenosyl-L-methionine.	No differences in mouse brain concentrations (44).	Increased levels in the post-mortem AD brain are associated with cognitive dysfunction and neurological hallmarks of AD (52). Increase Aβ formation in BV-2 microglial cells (53).	Induces pro-inflammatory activation in endothelial cells in vitro (54).
O-succinyl-L-homoserine	Methionine metabolism	Mediates the conversion of homocysteine into cystathionine (Fig. 5).	Not known	Not known	Not known

S-adenosyl-L-methionine	Methionine metabolism<br>Arginine and proline metabolism	Main donor of methyl groups for DNA methylation in the brain (43).<br>Dietary supplementation improves cognitive abilities in mice (55).	No differences in mouse brain concentrations (44).	Decreased levels in the post-mortem AD brain (56) and CSF of AD patients (57).	Inhibits TNFa production and enhances IL-6 and IL-10 secretion in LPS-stimulated human macrophages and/or murine monocytes (58, 59).
5'-methylthioadenosine	Methionine metabolism<br>Arginine and proline metabolism	Neuro-protective and anti-inflammatory derivative of methionine.	Not known	Increased CSF levels in MCI impaired patients (60).	Reduces brain damage; inhibits INFg and TNFa production and enhances IL-10 production in animal models of neuroinflammation (61).
N-carbamoylsarcosine	Arginine and proline metabolism	Not known	Not known	Not known	Not known
N-succinyl-L-glutamate 5-semialdehyde	Arginine and proline metabolism	Not known	Not known	Not known	Elevated in plasma from lung cancer patients harbouring a genetic mutation (7) which increases their susceptibility to inflammation (8).
Urea-1-carboxylate	Arginine and proline metabolism	Not known	Not known	Not known	Not known
Homoarginine	Arginine and proline metabolism	Precursor of the free radical nitric oxide.<br>Unclear role in healthy brain function (62).	Not known	Not known	Reduced plasma levels associated with increased C-reactive protein levels in chronic kidney disease patients (14).
L-aspartate	Arginine and proline metabolism<br>Lysine biosynthesis	Excitatory amino acid and selective glutamatergic NMDA receptor agonist (63).	Not known	Not known	No known
N6-(L-1,3-Dicarboxypropyl)-L-lysine	Lysine biosynthesis	Not known	Not known	Not known	Not known
N2-acetyl-L-aminoadipate	Lysine biosynthesis	Not known	Not known	Not known	Not known
L-2-aminoadipate	Lysine degradation	Intermediate in lysine degradation. Antagonises excitatory NMDA receptors and reduces kynurenine levels in the hippocampus (64).	Not known	Increased plasma levels in MCI and AD patients (65).	Produced by peritoneal cells in response to acute inflammation (66).<br>Inhibits kynurenine production by astrocytes (64).
L-pipecolate	Lysine degradation<br>Alkaloid biosynthesis I	Major degradation product of lysine in the murine brain (67).	Not known	Reduced CSF levels in MCI, but not AD, patients (60).	Urine levels are positively associated with low grade inflammation in healthy individuals (10).
(Z)-4-hydroxyphenylacetaldehyde-oxime	Tyrosine metabolism	Not known	Not known	Not known	Increased gut levels in inflammatory bowel disease (13).
[FA hydroxy,oxo(7:0/2:0)]	Tyrosine metabolism	Not known	Not known	Not known	Not known

4-hydroxy-2-oxo-Heptanedioic acid					
L-phenylalanine	Phenylalanine, tyrosine and tryptophan biosynthesis	Dietary precursor of catecholamines. Accumulation in the brain due to impaired degradation causes brain damage and mental retardation (68).	Not known	Increased circulating levels correlate with inflammation in a subgroup of AD patients (69).	Increased circulating levels in elderly people with chronic low grade inflammation (23).
L-tryptophan	Phenylalanine, tyrosine and tryptophan biosynthesis Tryptophan metabolism	Dietary precursor of serotonin and vitamin B3 (nicotinic acid). Improves mood and cognition by enhancing serotoninergic neurotransmission (70) and nicotinamide pathway (71)	Women are more susceptible to episodic memory impairment caused by acute tryptophan depletion (72). Lower plasma tryptophan levels in females associated with reduced serotonin synthesis rate throughout the brain (73).	Reduced CSF levels in MCI, but not AD, patients (60). Reduced serotoninergic neurotransmission associated with the development of cognitive symptoms in AD (74). Upregulation of kynurenine pathway associated with neurological hallmarks of AD (75).	Increases inflammation via stimulation of the kynurenine pathway (76). Serotoninergic neurotransmission thought to protect against neuroinflammation (77).
5-hydroxyindoleacetate	Tryptophan metabolism	End metabolite of the serotonin pathway of tryptophan metabolism (Fig. 6).	See L-tryptophan	Elevated CSF levels in MCI and AD (45).	See L-tryptophan
Pantothenate	beta-Alanine metabolism Pantothenate and CoA biosynthesis	Vitamin B5. Substrate for the biosynthesis of coenzyme A which contributes to the structure and function of brain cells *via* its role in the synthesis and oxidation of fatty acids (71).	Not known	Dietary intake positively associated with cerebral Aβ burden in MCI patients (78).	Dietary intake lower systemic inflammation (C-reactive protein levels) in healthy adults over 40 (11).
D-alanyl-D-alanine	D-Alanine metabolism Peptidoglycan biosynthesis	Not known	Not known	Not known	Anti-inflammatory antibiotic-binding protein (5).
Glutathione disulphide (GSSG)	Glutamate metabolism Glutathione metabolism	Toxic oxidation product of the anti-oxidant glutathione produced and exported by astrocytes in the brain (79).	No sex differences in brain tissue content with aging in mice despite the most pronounced decline in glutathione concentrations seen in males (80).	Higher activity of glutathione reductase activity, which catalyses the reduction of GSSG disulphide in glutathione, in the temporal cortex of AD patients (81), but unaltered GSSG contents (82).	Increased circulating levels during acute systemic inflammation in the rats (83).
Methylimidazoleacetic acid	Histidine metabolism	Main metabolite of histamine, a neuromodulator, also involved in cognition, wakefulness and anxiety and motivated behaviours (84, 85).	Not known	Degeneration of histaminergic nerve fibres in AD (86).	Histamine is produced by immune cells in the brain, induces pro-inflammatory microglial activation but inhibits LPS-induced microglial activation (87).
N(pi)-methyl-L-histidine	Histidine metabolism	Derivative of histidine, a precursor of brain histamine (85).	Not known	Not known	Serum histidine levels are negatively associated with systemic inflammation (C-reactive protein levels) in obese women (6).
Hypotaurine	Taurine and	Intermediate in the synthesis of	Not known	Not known	Suppresses inflammatory and

	hypotaurine metabolism	taurine from the methionine derivative cysteine (Fig. 5) in neurons, astrocytes and microglia (88, 89).			neuropathic pain (90).
Carbohydrate metabolism					
Succinate	Citrate cycle (TCA cycle)<br>Glyoxylate and dicarboxylate metabolism	Support brain energy metabolism by promoting ATP generation in mitochondria (91).<br>Ameliorates metabolic deficits of glial cells with mitochondrial dysfunction (92).	Not known	Reduced whole brain content from 9 months of age in an APP/PS1 mouse model (93).	Pro-inflammatory mediator in macrophages mediating LPS-induced IL-1β production (20, 94).
Isocitrate	Citrate cycle (TCA cycle)<br>Glyoxylate and dicarboxylate metabolism	Not known	Not known	Increased CSF levels in a transgenic rat model of tauopathy (95).	Anti-inflammatory in rat model of anaemia of inflammation (15).<br>Microglial deficiency in isocitrate dehydrogenase, the enzyme that catalyses oxidative decarboxylation of isocitrate, suppresses LP-induced pro-inflammatory cytokine production (TNFa, IL-6, IL-1β) (96).
(S)-malate	Citrate cycle (TCA cycle)<br>Pyruvate metabolism<br>Glyoxylate and dicarboxylate metabolism	Metabolite of the KREBS cycle which promotes mitochondrial ATP generation and can be recycled into pyruvate (97).	Not known	Not known	Produced by pro-inflammatory activation of macrophages (98).
(D)-lactate	Pyruvate metabolism	Produced by methylglyoxal metabolism. Excess levels can cause encephalopathy (99).	Sex differences in D-lactate metabolism may contribute to reduced association between microbiota and neurological symptoms in females (100).	Methylglyoxal can cause Aβ aggregation (101) and its neurotoxicity is associated with AD (102).	Methylglyoxal is pro-inflammatory and activates glial cells in the brain (103).
(D)-S-lactoylglutath ione	Pyruvate metabolism	Intermediate in the formation of D-lactate from methylglyoxal.	Not known	Not known	Not known
Phosphoenolpyruvate	Citrate cycle (TCA cycle)<br>Pyruvate metabolism<br>Glycolysis / Gluconeogenesis	Intermediate in glycolysis and gluconeogenesis.	Not known	Not known	Systemic LPS increases brain levels of phosphoenolpyruvate carboxykinase (104), which catalyses the conversion of oxaloacetate to phosphoenolpyruvate in gluconeogenesis and has anti-inflammatory effects on LPS-induced circulating pro-inflammatory cytokines levels (104) and macrophages phenotype

					(105).
Pyruvate	Citrate cycle (TCA cycle)<br>Glycolysis / Gluconeogenesis	Intermediate metabolite of glucose with potent antioxidant and anti-inflammatory properties (106).	Sex differences in pyruvate metabolism associated with reduced oxidative stress and damage in females (107, 108).	Improves cognitive performance in mouse models of AD without affecting tau or Aβ pathology (109, 110).	Ethyl derivatives of pyruvate alleviate pro-inflammatory changes in the brain (111, 112).
D-glyceraldehyde 3-phosphate	Glycolysis / Gluconeogenesis	Intermediate in glycolysis and gluconeogenesis.	Not known	Not known	Not known
3-phospho-D-glycerate	Glycolysis /Gluconeogenesis<br>Glyoxylate and dicarboxylate metabolism	Conversion of 3-phospho-D-glycerate to phosphohydroxypyruvate by the enzyme 3-phosphoglycerate dehydrogenase (3PGDH) is the first step in serine production.<br>Deficiency in 3PGDH causes brain atrophy, seizures and psychomotor retardation (113).	Not known	Not known	3PGDH is an astrocytic enzyme that catalases the production of serine by neurons and glia (114) and is anti-inflammatory in fibroblasts (115).
2-phosphoglycolate	Glyoxolate and dicatboxylate metabolism	Possible indicator of damage and repair of DNA ends (116).	Not known	Decreased expression and activity in AD brain of phosphoglucomutase 1 (PGM1) the glycolytic enzyme that catalyses the conversion of 3-phosphoglycerate to 2-phosphoglycerate (117).	Not known
3-oxalomalate	Glyoxolate and dicatboxylate metabolism	Not known	Not known	Not known	Antioxidant in LPS-activated macrophages (118).
L-rhamnose	Fructose and mannose metabolism	Not known	Not known	Not known	Inhibits pro-inflammatory cytokines production in macrophages (119).
D-sorbitol	Fructose and mannose metabolism	Intermediate in the production of fructose from glucose in the brain (120).	Not known	Elevated levels in the post-mortem AD brain (121).	Anti-inflammatory properties in resident cells from articular cartilage (122).
6-phospho-D-gluconate	Pentose phosphate pathway	Regulatory control of brain metabolism (123).	Not known	Increased activity of the pentose phosphate pathway associated with increased pro-oxidant activity in the post mortem AD brain (124)	Triggers pro-inflammatory cytokines secretion in LPS-activated macrophages (32).
Propanoyl phosphate	Propanoate metabolism<br>C5-Branched dibasic acid metabolism	Not known	Not known	Not known	Not known
Itaconate	C5-Branched dibasic acid metabolism<br>Citrate cycle (TCA cycle)	Endogenous antibiotic in the brain (125).	Not known	Not known	Anti-inflammatory metabolite found in macrophages (16) and produced by microglia (125).

Methyloxaloacetate	C5-Branched dibasic acid metabolism	Not known	Not known	Not known	Not known
Nucleotide metabolism					
Hypoxanthine	Purine metabolism	Endogenous ligand of benzodiazepine-binding sites in the brain (126).	Not known	Increased brain concentration in a transgenic rat model of tauopathy (95).<br>Elevated CSF levels in MCI patients (45).	Hypoxanthine is anti-inflammatory and depleted in LPS-stimulated macrophages (127).
Urate	Purine metabolism	Neuroprotective and antioxidant at physiological levels (128).	Reduced brain tissue contents in women (129).	Increased CSF contents in AD (130).<br>Trends towards reduced levels in the post-mortem AD brain (129).	High uric acid diet causes cognitive deficits by inducing hippocampal neuroinflammation (22).<br>Suppresses LPS-induced pro-inflammatory microglial activation in vitro (131).
N6-(1,2-Dicarboxyethyl)-AMP	Purine metabolism	Not known	Not known	Not known	Not known
Orotate	Pyrimidine metabolism	Not known	Not known	Not known	Not known
Thymidine	Pyrimidine metabolism	Not known	Not known	Not known	Not known
Thymine	Pyrimidine metabolism	Not known	Not known	Not known	Not known
5,6-dihydrouracil	Pyrimidine metabolism<br>Beta-Alanine metabolism<br>Pantothenate and CoA biosynthesis	Not known	Not known	Not known	Not known
Lipid metabolism and Fatty acyls					
Octadecanoic acid<br>(18:0 stearic acid)	Fatty acids biosynthesis<br>Biosynthesis of unsaturated fatty acids	Needed for the synthesis of membranes of neurons and astrocytes during brain development (132).	No sex differences in mouse brain content with normal diet (133).	Induces tau phosphorylation in cultured neurons and astrocytes (134).<br>Triggers secretion of Aβ peptide (135).<br>Reduced levels in the post-mortem AD brain (136).	Accumulates primarily in astrocytes (137) where it triggers the release of TNFα and IL-6 (138).
Hexadecanoic acid<br>(16:0 palmitic acid)	Biosynthesis of unsaturated fatty acids	Dietary administration, which enters the brain, improves cognitive and motor function (139).	No sex differences in mouse brain content with normal diet (133).	Induces tau phosphorylation and amyloid processing in cultured neurons and astrocytes (134, 135, 140).<br>Increased levels in the post-mortem AD brain (136).	Triggers astrocytic release of TNFα and IL-6 in vitro (138).<br>Impairs the protective migratory and phagocytic activities of microglia in both males and females (141).

Icosatrienoic acid 20:3	Biosynthesis of unsaturated fatty acids	Increased brain levels associated with reduced brain growth in developing rats fed with an essential fatty acid deficient diet (142). Intermediate in the synthesis of Arachidonic acid.	Not known	Not known	Not known
[FA (20:4)] 5Z,8Z,11Z,14Z-eicosatetraenoic acid (20:4 Arachidonic acid)	Fatty Acids and Conjugates	Contributes in brain growth and function in combination with other fatty acids (143).	No sex differences in mouse brain content with normal diet (133).	Triggers secretion of Aβ peptide (135). Reduced levels in the post-mortem AD brain (136, 144).	Precursor of potent pro-inflammatory eicosanoids (e.g. prostaglandins) in astrocytes (145). Does not trigger pro-inflammatory cytokine release in astrocytes (138).
PGH2-EA	Eicosanoids	Metabolite of the endocannabinoid anandamide known to modulate the brain reward system (146)	Not known	Increases the neurotoxicity of Aβ peptide (147).	Precursor of prostaglandin E2 which mediates LPS-induced sickness (148).
Formyl 3-hydroxy-butanoate	Fatty esters	Not known	Not known	Not known	Not known
sn-glycerol 3-phosphate	Glycerolipid metabolism Glycerophospholipid metabolism	Intermediate in the glycolysis metabolic pathway. Increased biosynthesis in the hippocampus during long-term potentiation (149).	Not known	Not known	Not known
sn-glycero-3-Phosphoethanolamine	Glycerophospholipid metabolism Ether lipid metabolism	Not known.	Not known	Not known	Not known
Energy Metabolism					
ATP	Oxidative phosphorylation Purine metabolism	Main cellular source of energy in the brain, which can improve cognitive function (150).	Greater ATP production in female mitochondria in the rodent brain (107)	Decreased brain contents in a transgenic rat model of tauopathy (95) and mouse model of amyloidosis (151).	Pro-inflammatory signalling molecule in the brain via increased synthesis of prostaglandin E2 (19). Produced by microglia and astrocytes, leading to microglial activation and chemotactic factor for microglia towards tissue injury (152, 153)
D-fructose 1,6-bisphosphate	Carbon fixation	Neuroprotective high energy glycolytic intermediate (154).	Not known	Not known	Anti-inflammatory in pain models (155)
D-sedoheptulose 1,7-bisphosphate	Carbon fixation	Not known	Not known	Not known	Not known
Metabolism of Cofactors and Vitamins					
Iminoglycine	Thiamine metabolism	Not known	Not known	Not known	Not known

N1-methyl-2-pyridone-5-carboxamide	Nicotinate and nicotinamide metabolism	Toxic end metabolite of the tryptophan-nicotinamide pathway (156)	Not known	Not known	Elevated circulating levels associated with systemic inflammation (157).
Peptides					
Gamma glutamylglutamic acid	Peptide	Not known	Not known	Not known	Not known
L-beta-aspartyl-L-glutamicacid	Peptide	Not known	Not known	Not known	Belongs to the family of N-acyl-alpha amino acids and derivatives which are known for their anti-inflammatory action (1)
Asp-Ser-His	Basic peptide	Not known	Not known	Not known	Not known
Asn-Met-Met-Asn	Hydrophobic peptide	Not known	Not known	Not known	Not known
Asp-Phe-Thr-Thr	Hydrophobic peptide	Not known	Not known	Not known	Not known
Asn-Asn-Asn	Polar peptide	Not known	Not known	Not known	Not known
Biosynthesis of Polyketides and nonribosomal Peptides					
Narbomycin	Biosynthesis of 12-, 14- and 16-membered macrolides	Not known	Not known	Not known	Not known
13-dihydrocarminomycin	Biosynthesis of type II polyketide products	Not known	Not known	Not known	Not known
Biosynthesis of Secondary metabolites					
Dihydroclavaminic acid $C_8H_{12}N_2O_4$	Clavulanic acid biosynthesis	Not known	Not known	Not known	Not known
**Not known**					
γ-aminobutyramide $C_4H_{10}N_2O$	Not known	Not known	Not known	Not known	Not known
1-deoxy-D-altro-heptulose 7-phosphate	Not known	Not known	Not known	Not known	Not known
1-methyladenosine	Not known			Increased urinary levels in patients with mild-to-moderate Alzheimer's disease (3).	Immunosuppressive on macrophage function (2).
3, 5-tetradecadiencarnitine	Not known	Not known	Not known	Not known	Not known
3-methylphosphoenol-pyruvate	Not known	Not known	Not known	Not known	Not known
6-methyltetrahydropterin	Not known	Not known	Not known	Not known	Not known

Athamantin	Not known	Not known	Not known	Not known	Not known
Camptothecin	Not known	Not known	Not known	Not known	Not known
Dimethyl citraconate	Not known	Not known	Not known	Not known	Not known
DL-2-sulfoctanoicacid	Not known	Not known	Not known	Not known	Not known
Elaidiccarnitine	Not known	Not known	Not known	Not known	Not known
Glutarylcarnitine	Not known	Not known	Not known	Not known	Not known
Glycerophosphoglycerol	Not known	Not known	Not known	Not known	Not known
Linoelaidylcarnitine	Not known	Not known	Not known	Not known	Not known
N-acetyl-(L)-arginine	Not known	Not known	Not known	Not known	Not known
NG,NG-dimethyl-L-arginine	Not known	Not known	Not known	Not known	Not known
Nocardicin C	Not known	Not known	Not known	Not known	Not known
Nonulose 9-phosphate	Not known	Not known	Not known	Not known	Not known
Norepinephrinesulfate	Not known	Not known	Not known	Not known	Not known
Orotidine	Not known	Not known	Not known	Not known	Not known
Tetradecanoylcarnitine	Not known	Not known	Not known	Not known	Not known
GDP-3,6-dideoxy-D-galactose	Not known	Not known	Not known	Not known	Not known
N-hydroxyvaline	Linamarin biosynthesis	Not known	Not known	Not known	Not known

References

1. Burstein SH. N-Acyl Amino Acids (Elmiric Acids): Endogenous Signaling Molecules with Therapeutic Potential. Mol Pharmacol. 2018;93(3):228-38.
2. Itoh K, Majima T, Edo K, Mizugaki M, Ishida N. Suppressive effect of 1-methyladenosine on the generation of chemiluminescence by mouse peritoneal macrophages stimulated with opsonized zymosan. Tohoku J Exp Med. 1989;157(3):205-14.
3. Lee SH, Kim I, Chung BC. Increased urinary level of oxidized nucleosides in patients with mild-to-moderate Alzheimer's disease. Clin Biochem. 2007;40(13-14):936-8.
4. Kato H, Miura K, Nakano S, Suzuki K, Bannai M, Inoue Y. Leucine-enriched essential amino acids attenuate inflammation in rat muscle and enhance muscle repair after eccentric contraction. Amino Acids. 2016;48(9):2145-55.
5. van der Aart LT, Lemmens N, van Wamel WJ, van Wezel GP. Substrate Inhibition of VanA by d-Alanine Reduces Vancomycin Resistance in a VanX-Dependent Manner. Antimicrob Agents Chemother. 2016;60(8):4930-9.
6. Niu YC, Feng RN, Hou Y, Li K, Kang Z, Wang J, et al. Histidine and arginine are associated with inflammation and oxidative stress in obese women. Br J Nutr. 2012;108(1):57-61.
7. Pamungkas AD, Medriano CA, Sim E, Lee S, Park YH. A pilot study identifying a potential plasma biomarker for determining EGFR mutations in exons 19 or 21 in lung cancer patients. Mol Med Rep. 2017;15(6):4155-61.
8. Jacobs JM, Traeger L, Eusebio J, Simon NM, Sequist LV, Greer JA, et al. Depression, inflammation, and epidermal growth factor receptor (EGFR) status in metastatic non-small cell lung cancer: A pilot study. J Psychosom Res. 2017;99:28-33.
9. Han H, Yin J, Wang B, Huang XG, Yao JM, Zheng J, et al. Effects of dietary lysine restriction on inflammatory responses in piglets. Sci Rep-Uk. 2018;8.
10. Pietzner M, Kaul A, Henning AK, Kastenmuller G, Artati A, Lerch MM, et al. Comprehensive metabolic profiling of chronic low-grade inflammation among generally healthy individuals. BMC Med. 2017;15(1):210.
11. Jung S, Kim MK, Choi BY. The long-term relationship between dietary pantothenic acid (vitamin B-5) intake and C-reactive protein concentration in adults aged 40 years and older. Nutr Metab Cardiovas. 2017;27(9):806-16.
12. Pongratz G, Straub RH. The sympathetic nervous response in inflammation. Arthritis Res Ther. 2014;16(6):504.
13. Jansson J, Willing B, Lucio M, Fekete A, Dicksved J, Halfvarson J, et al. Metabolomics reveals metabolic biomarkers of Crohn's disease. PLoS One. 2009;4(7):e6386.
14. Ravani P, Maas R, Malberti F, Pecchini P, Mieth M, Quinn R, et al. Homoarginine and mortality in pre-dialysis chronic kidney disease (CKD) patients. PLoS One. 2013;8(9):e72694.
15. Richardson CL, Delehanty LL, Bullock GC, Rival CM, Tung KS, Kimpel DL, et al. Isocitrate ameliorates anemia by suppressing the erythroid iron restriction response. J Clin Invest. 2013;123(8):3614-23.
16. Mills EL, Ryan DG, Prag HA, Dikovskaya D, Menon D, Zaslona Z, et al. Itaconate is an anti-inflammatory metabolite that activates Nrf2 via alkylation of KEAP1. Nature. 2018;556(7699):113-+.
17. Kuda O, Brezinova M, Rombaldova M, Slavikova B, Posta M, Beier P, et al. Docosahexaenoic Acid-Derived Fatty Acid Esters of Hydroxy Fatty Acids (FAHFAs) With Anti-inflammatory Properties. Diabetes. 2016;65(9):2580-90.
18. Nofal ZM, Fahmy HH, Zarea ES, El-Eraky W. Synthesis of New Pyrimidine Derivatives with Evaluation of Their Anti-Inflammatory and Analgesic Activities. Acta Pol Pharm. 2011;68(4):507-17.
19. Fiebich BL, Akter S, Akundi RS. The two-hit hypothesis for neuroinflammation: role of exogenous ATP in modulating inflammation in the brain. Front Cell Neurosci. 2014;8:260.
20. Mills E, O'Neill LA. Succinate: a metabolic signal in inflammation. Trends Cell Biol. 2014;24(5):313-20.
21. McKay TB, Hjortdal J, Sejersen H, Asara JM, Wu J, Karamichos D. Endocrine and Metabolic Pathways Linked to Keratoconus: Implications for the Role of Hormones in the Stromal Microenvironment. Sci Rep. 2016;6:25534.
22. Shao XN, Lu WJ, Gao FB, Li DD, Hu J, Li Y, et al. Uric Acid Induces Cognitive Dysfunction through Hippocampal Inflammation in Rodents and Humans. J Neurosci. 2016;36(43):10990-1005.
23. Capuron L, Schroecksnadel S, Feart C, Aubert A, Higueret D, Barberger-Gateau P, et al. Chronic Low-Grade Inflammation in Elderly Persons Is Associated with Altered Tryptophan and Tyrosine Metabolism: Role in Neuropsychiatric Symptoms. Biol Psychiat. 2011;70(2):175-82.
24. Aoki T, Narumiya S. Prostaglandins and chronic inflammation. Trends Pharmacol Sci. 2012;33(6):304-11.
25. Saper CB, Romanovsky AA, Scammell TE. Neural circuitry engaged by prostaglandins during the sickness syndrome. Nat Neurosci. 2012;15(8):1088-95.

26. Branco ACCC, Yoshikawa FSY, Pietrobon AJ, Sato MN. Role of Histamine in Modulating the Immune Response and Inflammation. Mediat Inflamm. 2018.
27. Wang H, Yu M, Ochani M, Amella CA, Tanovic M, Susarla S, et al. Nicotinic acetylcholine receptor alpha 7 subunit is an essential regulator of inflammation. Nature. 2003;421(6921):384-8.
28. Rowley TJ, McKinstry A, Greenidge E, Smith W, Flood P. Antinociceptive and anti-inflammatory effects of choline in a mouse model of postoperative pain. Brit J Anaesth. 2010;105(2):201-7.
29. Parrish WR, Puerta MG, Ochani M, Ochani K, Moskovic D, Lin X, et al. Choline suppresses inflammatory responses. Shock. 2006;25:45-.
30. Watanabe Y, Tateno H, Nakamura-Tsuruta S, Kominami J, Hirabayashi J, Nakamura O, et al. The function of rhamnose-binding lectin in innate immunity by restricted binding to Gb3. Dev Comp Immunol. 2009;33(2):187-97.
31. Lee JS, Wang RX, Alexeev EE, Lanis JM, Battista KD, Glover LE, et al. Hypoxanthine is a checkpoint stress metabolite in colonic epithelial energy modulation and barrier function. J Biol Chem. 2018;293(16):6039-51.
32. Baardman J, Verberk SGS, Prange KHM, van Weeghel M, van der Velden S, Ryan DG, et al. A Defective Pentose Phosphate Pathway Reduces Inflammatory Macrophage Responses during Hypercholesterolemia. Cell Rep. 2018;25(8):2044-52 e5.
33. Wu T, Wang C, Ding L, Shen Y, Cui H, Wang M, et al. Arginine Relieves the Inflammatory Response and Enhances the Casein Expression in Bovine Mammary Epithelial Cells Induced by Lipopolysaccharide. Mediators Inflamm. 2016;2016:9618795.
34. Yudkoff M, Daikhin Y, Nissim I, Horyn O, Luhovyy B, Luhovyy B, et al. Brain amino acid requirements and toxicity: the example of leucine. J Nutr. 2005;135(6 Suppl):1531S-8S.
35. Li HJ, Ye D, Xie W, Hua F, Yang YL, Wu J, et al. Defect of branched-chain amino acid metabolism promotes the development of Alzheimer's disease by targeting the mTOR signaling. Bioscience Rep. 2018;38.
36. Loffelholz K, Klein J, Koppen A. Choline, a Precursor of Acetylcholine and Phospholipids in the Brain. Progress in Brain Research. 1993;98:197-200.
37. Blusztajn JK, Slack BE, Mellott TJ. Neuroprotective Actions of Dietary Choline. Nutrients. 2017;9(8).
38. Chen CS, Kuo YT, Tsai HY, Li CW, Lee CC, Yen CF, et al. Brain Biochemical Correlates of the Plasma Homocysteine Level: A Proton Magnetic Resonance Spectroscopy Study in the Elderly Subjects. Am J Geriat Psychiat. 2011;19(7):618-26.
39. Francis PT, Palmer AM, Snape M, Wilcock GK. The cholinergic hypothesis of Alzheimer's disease: a review of progress. J Neurol Neurosur Ps. 1999;66(2):137-47.
40. Hensley K, Denton TT. Alternative functions of the brain transsulfuration pathway represent an underappreciated aspect of brain redox biochemistry with significant potential for therapeutic engagement. Free Radical Bio Med. 2015;78:123-34.
41. Gueli MC, Taibi G. Alzheimer's disease: amino acid levels and brain metabolic status. Neurol Sci. 2013;34(9):1575-9.
42. Zhu MZ, Du JB, Liu AD, Holmberg L, Chen SY, Bu DF, et al. L-cystathionine inhibits oxidized low density lipoprotein-induced THP-1-derived macrophage inflammatory cytokine monocyte chemoattractant protein-1 generation via the NF-kappa B pathway. Sci Rep-Uk. 2015;5.
43. McGowan PO, Meaney MJ, Szyf M. Diet and the epigenetic (re)programming of phenotypic differences in behavior. Brain Res. 2008;1237:12-24.
44. Witham KL, Butcher NJ, Sugamori KS, Brenneman D, Grant DM, Minchin RF. 5-methyl-tetrahydrofolate and the S-adenosylmethionine cycle in C57BL/6J mouse tissues: gender differences and effects of arylamine N-acetyltransferase-1 deletion. PLoS One. 2013;8(10):e77923.
45. Kaddurah-Daouk R, Zhu H, Sharma S, Bogdanov M, Rozen SG, Matson W, et al. Alterations in metabolic pathways and networks in Alzheimer's disease. Transl Psychiat. 2013;3.
46. Tapia-Rojas C, Lindsay CB, Montecinos-Oliva C, Arrazola MS, Retamales RM, Bunout D, et al. Is L-methionine a trigger factor for Alzheimer's-like neurodegeneration?: Changes in Abeta oligomers, tau phosphorylation, synaptic proteins, Wnt signaling and behavioral impairment in wild-type mice. Mol Neurodegener. 2015;10:62.
47. Dos Santos LM, da Silva TM, Azambuja JH, Ramos PT, Oliveira PS, da Silveira EF, et al. Methionine and methionine sulfoxide treatment induces M1/classical macrophage polarization and modulates oxidative stress and purinergic signaling parameters. Mol Cell Biochem. 2017;424(1-2):69-78.
48. Stadtman ER, Van Remmen H, Richardson A, Wehr NB, Levine RL. Methionine oxidation and aging. Biochim Biophys Acta. 2005;1703(2):135-40.
49. Moskovitz J, Du F, Bowman CF, Yan SS. Methionine sulfoxide reductase A affects beta-amyloid solubility and mitochondrial function in a mouse model of Alzheimer's disease. Am J Physiol Endocrinol Metab. 2016;310(6):E388-93.

50. Fan H, Wu PF, Zhang L, Hu ZL, Wang W, Guan XL, et al. Methionine Sulfoxide Reductase A Negatively Controls Microglia-Mediated Neuroinflammation via Inhibiting ROS/MAPKs/NF-kappa B Signaling Pathways Through a Catalytic Antioxidant Function. Antioxid Redox Sign. 2015;22(10):832-47.
51. Chen S, Dong ZP, Cheng M, Zhao YQ, Wang MY, Sai N, et al. Homocysteine exaggerates microglia activation and neuroinflammation through microglia localized STAT3 overactivation following ischemic stroke. J Neuroinflamm. 2017;14.
52. Kennedy BP, Bottiglieri T, Arning E, Ziegler MG, Hansen LA, Masliah E. Elevated S-adenosylhomocysteine in Alzheimer brain: influence on methyltransferases and cognitive function. J Neural Transm (Vienna). 2004;111(4):547-67.
53. Lin HC, Hsieh HM, Chen YH, Hu ML. S-Adenosylhomocysteine increases beta-amyloid formation in BV-2 microglial cells by increased expressions of beta-amyloid precursor protein and presenilin 1 and by hypomethylation of these gene promoters. Neurotoxicology. 2009;30(4):622-7.
54. Barroso M, Kao D, Blom HJ, de Almeida IT, Castro R, Loscalzo J, et al. S-adenosylhomocysteine induces inflammation through NFkB: A possible role for EZH2 in endothelial cell activation. Bba-Mol Basis Dis. 2016;1862(1):82-92.
55. Montgomery SE, Sepehry AA, Wangsgaard JD, Koenig JE. The Effect of S-Adenosylmethionine on Cognitive Performance in Mice: An Animal Model Meta-Analysis. Plos One. 2014;9(10).
56. Morrison LD, Smith DD, Kish SJ. Brain S-adenosylmethionine levels are severely decreased in Alzheimer's disease. J Neurochem. 1996;67(3):1328-31.
57. Linnebank M, Popp J, Smulders Y, Smith D, Semmler A, Farkas M, et al. S-Adenosylmethionine Is Decreased in the Cerebrospinal Fluid of Patients with Alzheimer's Disease. Neurodegener Dis. 2010;7(6):373-8.
58. Pfalzer AC, Choi SW, Tammen SA, Park LK, Bottiglieri T, Parnell LD, et al. S-adenosylmethionine mediates inhibition of inflammatory response and changes in DNA methylation in human macrophages. Physiol Genomics. 2014;46(17):617-23.
59. Song ZY, Chen T, Uriarte S, Hill D, Barve S, McClain C. S-Adenosylmethionine (SAMe) modulates interleukin-10 and interleukin-6, but not TNF, production by adenosine (A2) receptor. Gastroenterology. 2004;126(4):A684-A.
60. Ibanez C, Simo C, Barupal DK, Fiehn O, Kivipelto M, Cedazo-Minguez A, et al. A new metabolomic workflow for early detection of Alzheimer's disease. J Chromatogr A. 2013;1302:65-71.
61. Moreno B, Hevia H, Santamaria M, Sepulcre J, Munoz J, Garcia-Trevijano ER, et al. Methylthioadenosine reverses brain autoimmune disease. Ann Neurol. 2006;60(3):323-34.
62. Bernstein HG, Jager K, Dobrowolny H, Steiner J, Keilhoff G, Bogerts B, et al. Possible sources and functions of L-homoarginine in the brain: review of the literature and own findings. Amino Acids. 2015;47(9):1729-40.
63. Herring BE, Silm K, Edwards RH, Nicoll RA. Is Aspartate an Excitatory Neurotransmitter? J Neurosci. 2015;35(28):10168-71.
64. Wu HQ, Ungerstedt U, Schwarcz R. L-Alpha-Aminoadipic Acid as a Regulator of Kynurenic Acid Production in the Hippocampus - a Microdialysis Study in Freely Moving Rats. Eur J Pharmacol. 1995;281(1):55-61.
65. Wang G, Zhou Y, Huang FJ, Tang HD, Xu XH, Liu JJ, et al. Plasma Metabolite Profiles of Alzheimer's Disease and Mild Cognitive Impairment. J Proteome Res. 2014;13(5):2649-58.
66. Lin H, Levison BS, Buffa JA, Huang Y, Fu X, Wang Z, et al. Myeloperoxidase-mediated protein lysine oxidation generates 2-aminoadipic acid and lysine nitrile in vivo. Free Radical Bio Med. 2017;104:20-31.
67. Posset R, Opp S, Struys EA, Volkl A, Mohr H, Hoffmann GF, et al. Understanding cerebral L-lysine metabolism: the role of L-pipecolate metabolism in Gcdh-deficient mice as a model for glutaric aciduria type I. J Inherit Metab Dis. 2015;38(2):265-72.
68. Williams RA, Mamotte CD, Burnett JR. Phenylketonuria: an inborn error of phenylalanine metabolism. Clin Biochem Rev. 2008;29(1):31-41.
69. Wissmann P, Geisler S, Leblhuber F, Fuchs D. Immune activation in patients with Alzheimer's disease is associated with high serum phenylalanine concentrations. J Neurol Sci. 2013;329(1-2):29-33.
70. Jenkins TA, Nguyen JC, Polglaze KE, Bertrand PP. Influence of Tryptophan and Serotonin on Mood and Cognition with a Possible Role of the Gut-Brain Axis. Nutrients. 2016;8(1).
71. Kennedy DO. B Vitamins and the Brain: Mechanisms, Dose and Efficacy-A Review. Nutrients. 2016;8(2).
72. Sambeth A, Blokland A, Harmer CJ, Kilkens TO, Nathan PJ, Porter RJ, et al. Sex differences in the effect of acute tryptophan depletion on declarative episodic memory: a pooled analysis of nine studies. Neurosci Biobehav Rev. 2007;31(4):516-29.
73. Nishizawa S, Benkelfat C, Young SN, Leyton M, Mzengeza S, DeMontigny C, et al. Differences between males and females in rates of serotonin synthesis in human brain. P Natl Acad Sci USA. 1997;94(10):5308-13.

74. Smith GS, Barrett FS, Joo JH, Nassery N, Savonenko A, Sodums DJ, et al. Molecular imaging of serotonin degeneration in mild cognitive impairment. Neurobiol Dis. 2017;105:33-41.
75. Wu W, Nicolazzo JA, Wen L, Chung R, Stankovic R, Bao SSS, et al. Expression of Tryptophan 2,3-Dioxygenase and Production of Kynurenine Pathway Metabolites in Triple Transgenic Mice and Human Alzheimer's Disease Brain. Plos One. 2013;8(4).
76. Davis I, Liu AM. What is the tryptophan kynurenine pathway and why is it important to neurotherapeutics? Expert Rev Neurother. 2015;15(7):719-21.
77. Wu H, Denna TH, Storkersen JN, Gerriets VA. Beyond a neurotransmitter: The role of serotonin in inflammation and immunity. Pharmacol Res. 2019;140:100-14.
78. Lee JH, Ahn SY, Lee HA, Won KS, Chang HW, Oh JS, et al. Dietary intake of pantothenic acid is associated with cerebral amyloid burden in patients with cognitive impairment. Food Nutr Res. 2018;62.
79. Dringen R, Hirrlinger J. Glutathione pathways in the brain. Biol Chem. 2003;384(4):505-16.
80. Wang H, Liu HL, Liu RM. Gender difference in glutathione metabolism during aging in mice. Exp Gerontol. 2003;38(5):507-17.
81. Schuessel K, Leutner S, Cairns NJ, Muller WE, Eckert A. Impact of gender on upregulation of antioxidant defence mechanisms in Alzheimer's disease brain. J Neural Transm. 2004;111(9):1167-82.
82. Adams JD, Jr., Klaidman LK, Odunze IN, Shen HC, Miller CA. Alzheimer's and Parkinson's disease. Brain levels of glutathione, glutathione disulfide, and vitamin E. Mol Chem Neuropathol. 1991;14(3):213-26.
83. Ikegami K, Lalonde C, Young YK, Picard L, Demling R. Comparison of plasma reduced glutathione and oxidized glutathione with lung and liver tissue oxidant and antioxidant activity during acute inflammation. Shock. 1994;1(4):307-12.
84. Passani MB, Panula P, Lin JS. Histamine in the brain. Front Syst Neurosci. 2014;8:64.
85. Yoshikawa T, Nakamura T, Shibakusa T, Sugita M, Naganuma F, Iida T, et al. Insufficient intake of L-histidine reduces brain histamine and causes anxiety-like behaviors in male mice. J Nutr. 2014;144(10):1637-41.
86. Panula P, Rinne J, Kuokkanen K, Eriksson KS, Sallmen T, Kalimo H, et al. Neuronal histamine deficit in Alzheimer's disease. Neuroscience. 1998;82(4):993-7.
87. Barata-Antunes S, Cristovao AC, Pires J, Rocha SM, Bernardino L. Dual role of histamine on microglia-induced neurodegeneration. Biochim Biophys Acta Mol Basis Dis. 2017;1863(3):764-9.
88. Vitvitsky V, Garg SK, Banerjee R. Taurine biosynthesis by neurons and astrocytes. J Biol Chem. 2011;286(37):32002-10.
89. Brand A, Leibfritz D, Hamprecht B, Dringen R. Metabolism of cysteine in astroglial cells: synthesis of hypotaurine and taurine. J Neurochem. 1998;71(2):827-32.
90. Hara K, Nakamura M, Haranishi Y, Terada T, Kataoka K, Sata T. Antinociceptive effect of intrathecal administration of hypotaurine in rat models of inflammatory and neuropathic pain. Amino Acids. 2012;43(1):397-404.
91. Stovell MG, Mada MO, Helmy A, Carpenter TA, Thelin EP, Yan JL, et al. The effect of succinate on brain NADH/NAD(+) redox state and high energy phosphate metabolism in acute traumatic brain injury. Sci Rep. 2018;8(1):11140.
92. Giorgi-Coll S, Amaral AI, Hutchinson PJA, Kotter MR, Carpenter KLH. Succinate supplementation improves metabolic performance of mixed glial cell cultures with mitochondrial dysfunction. Sci Rep. 2017;7(1):1003.
93. Forster DM, James MF, Williams SR. Effects of Alzheimer's disease transgenes on neurochemical expression in the mouse brain determined by 1H MRS in vitro. Nmr Biomed. 2012;25(1):52-8.
94. Tannahill GM, Curtis AM, Adamik J, Palsson-McDermott EM, McGettrick AF, Goel G, et al. Succinate is an inflammatory signal that induces IL-1 beta through HIF-1 alpha. Nature. 2013;496(7444):238-+.
95. Karlikova R, Micova K, Najdekr L, Gardlo A, Adam T, Majerova P, et al. Metabolic status of CSF distinguishes rats with tauopathy from controls. Alzheimers Res Ther. 2017;9.
96. Chae U, Kim HS, Kim KM, Lee H, Lee HS, Park JW, et al. IDH2 Deficiency in Microglia Decreases the Pro-inflammatory Response via the ERK and NF-kappaB Pathways. Inflammation. 2018;41(5):1965-73.
97. Manjeri GR, Rodenburg RJ, Blanchet L, Roelofs S, Nijtmans LG, Smeitink JA, et al. Increased mitochondrial ATP production capacity in brain of healthy mice and a mouse model of isolated complex I deficiency after isoflurane anesthesia. J Inherit Metab Dis. 2016;39(1):59-65.
98. O'Neill LA. A broken krebs cycle in macrophages. Immunity. 2015;42(3):393-4.
99. Bosoi CR, Rose CF. Elevated cerebral lactate: Implications in the pathogenesis of hepatic encephalopathy. Metab Brain Dis. 2014;29(4):919-25.
100. Wallis A, Butt H, Ball M, Lewis DP, Bruck D. Support for the microgenderome invites enquiry into sex differences. Gut Microbes. 2017;8(1):46-52.

101. Woltjer RL, Maezawa I, Ou JJ, Montine KS, Montine TJ. Advanced glycation endproduct precursor alters intracellular amyloid-beta/A beta PP carboxy-terminal fragment aggregation and cytotoxicity. J Alzheimers Dis. 2003;5(6):467-76.
102. Angeloni C, Zambonin L, Hrelia S. Role of methylglyoxal in Alzheimer's disease. Biomed Res Int. 2014;2014:238485.
103. Allaman I, Belanger M, Magistretti PJ. Methylglyoxal, the dark side of glycolysis. Front Neurosci-Switz. 2015;9.
104. Sadasivam M, Ramatchandirin B, Balakrishnan S, Selvaraj K, Prahalathan C. The role of phosphoenolpyruvate carboxykinase in neuronal steroidogenesis under acute inflammation. Gene. 2014;552(2):249-54.
105. Ko CW, Counihan D, Wu J, Hatzoglou M, Puchowicz MA, Croniger CM. Macrophages with a deletion of the phosphoenolpyruvate carboxykinase 1 (Pck1) gene have a more proinflammatory phenotype. Journal of Biological Chemistry. 2018;293(9):3399-409.
106. Das UN. Pyruvate is an endogenous anti-inflammatory and anti-oxidant molecule. Med Sci Monitor. 2006;12(5):Ra79-Ra84.
107. Gaignard P, Savouroux S, Liere P, Pianos A, Therond P, Schumacher M, et al. Effect of Sex Differences on Brain Mitochondrial Function and Its Suppression by Ovariectomy and in Aged Mice. Endocrinology. 2015;156(8):2893-904.
108. Wagner AK, Bayir H, Ren DX, Puccio A, Zafonte RD, Kochanek PM. Relationships between cerebrospinal fluid markers of excitotoxicity, ischemia, and oxidative damage after severe TBI: The impact of gender, age, and hypothermia. J Neurotraum. 2004;21(2):125-36.
109. Isopi E, Granzotto A, Corona C, Bomba M, Ciavardelli D, Curcio M, et al. Pyruvate prevents the development of age-dependent cognitive deficits in a mouse model of Alzheimer's disease without reducing amyloid and tau pathology. Neurobiology of Disease. 2015;81:214-24.
110. Koivisto H, Leinonen H, Puurula M, Hafez HS, Barrera GA, Stridh MH, et al. Chronic Pyruvate Supplementation Increases Exploratory Activity and Brain Energy Reserves in Young and Middle-Aged Mice. Front Aging Neurosci. 2016;8:41.
111. Lee HK, Kim ID, Kim SW, Lee H, Park JY, Yoon SH, et al. Anti-inflammatory and anti-excitoxic effects of diethyl oxopropanamide, an ethyl pyruvate bioisoster, exert robust neuroprotective effects in the postischemic brain. Sci Rep. 2017;7:42891.
112. Lee HK, Kim SW, Jin Y, Kim ID, Park JY, Yoon SH, et al. Anti-inflammatory effects of OBA-09, a salicylic acid/pyruvate ester, in the postischemic brain. Brain Res. 2013;1528:68-79.
113. Tabatabaie L, Klomp LWJ, Rubio-Gozalbo ME, Spaapen LJM, Haagen AAM, Dorland L, et al. Expanding the clinical spectrum of 3-phosphoglycerate dehydrogenase deficiency. J Inherit Metab Dis. 2011;34(1):181-4.
114. Ehmsen JT, Ma TM, Sason H, Rosenberg D, Ogo T, Furuya S, et al. D-Serine in Glia and Neurons Derives from 3-Phosphoglycerate Dehydrogenase. J Neurosci. 2013;33(30):12464-9.
115. Hamano M, Haraguchi Y, Sayano T, Zyao C, Arimoto Y, Kawano Y, et al. Enhanced vulnerability to oxidative stress and induction of inflammatory gene expression in 3-phosphoglycerate dehydrogenase-deficient fibroblasts. Febs Open Bio. 2018;8(6):914-22.
116. Knight J, Hinsdale M, Holmes R. Glycolate and 2-phosphoglycolate content of tissues measured by ion chromatography coupled to mass spectrometry. Anal Biochem. 2012;421(1):121-4.
117. Newman SF, Sultana R, Perluigi M, Coccia R, Cai J, Pierce WM, et al. An increase in S-glutathionylated proteins in the Alzheimer's disease inferior parietal lobule, a proteomics approach. J Neurosci Res. 2007;85(7):1506-14.
118. Irace C, Esposito G, Maffettone C, Rossi A, Festa M, Iuvone T, et al. Oxalomalate affects the inducible nitric oxide synthase expression and activity. Life Sci. 2007;80(14):1282-91.
119. Watanabe Y, Tateno H, Nakamura-Tsuruta S, Kominami J, Hirabayashi J, Nakamura O, et al. The function of rhamnose-binding lectin in innate immunity by restricted binding to Gb3. Dev Comp Immunol. 2009;33(2):187-97.
120. Hwang JJ, Jiang L, Hamza M, Dai F, Belfort-DeAguiar R, Cline G, et al. The human brain produces fructose from glucose. JCI Insight. 2017;2(4):e90508.
121. Xu JS, Begley P, Church SJ, Patassini S, McHarg S, Kureishy N, et al. Elevation of brain glucose and polyol-pathway intermediates with accompanying brain-copper deficiency in patients with Alzheimer's disease: metabolic basis for dementia. Sci Rep-Uk. 2016;6.
122. Mongkhon JM, Thach M, Shi Q, Fernandes JC, Fahmi H, Benderdour M. Sorbitol-modified hyaluronic acid reduces oxidative stress, apoptosis and mediators of inflammation and catabolism in human osteoarthritic chondrocytes. Inflamm Res. 2014;63(8):691-701.
123. Kauffman FC, Brown JG, Passonneau JV, Lowry OH. Effects of Changes in Brain Metabolism on Levels of Pentose Phosphate Pathway Intermediates. Journal of Biological Chemistry. 1969;244(13):3647-+.
124. Palmer AM. The activity of the pentose phosphate pathway is increased in response to oxidative stress in Alzheimer's disease. J Neural Transm. 1999;106(3-4):317-28.

125. Michelucci A, Cordes T, Ghelfi J, Pailot A, Reiling N, Goldmann O, et al. Immune-responsive gene 1 protein links metabolism to immunity by catalyzing itaconic acid production. P Natl Acad Sci USA. 2013;110(19):7820-5.
126. Asano T, Spector S. Identification of Inosine and Hypoxanthine as Endogenous Ligands for the Brain Benzodiazepine-Binding Sites. P Natl Acad Sci USA. 1979;76(2):977-81.
127. Rattigan KM, Pountain AW, Regnault C, Achcar F, Vincent IM, Goodyear CS, et al. Metabolomic profiling of macrophages determines the discrete metabolomic signature and metabolomic interactome triggered by polarising immune stimuli. Plos One. 2018;13(3).
128. Fang P, Li X, Luo JJ, Wang H, Yang XF. A Double-edged Sword: Uric Acid and Neurological Disorders. Brain Disord Ther. 2013;2(2):109.
129. McFarland NR, Burdett T, Desjardins CA, Frosch MP, Schwarzschild MA. Postmortem brain levels of urate and precursors in Parkinson's disease and related disorders. Neurodegener Dis. 2013;12(4):189-98.
130. Tohgi H, Abe T, Takahashi S, Kikuchi T. The urate and xanthine concentrations in the cerebrospinal fluid in patients with vascular dementia of the Binswanger type, Alzheimer type dementia, and Parkinson's disease. J Neural Transm Park Dis Dement Sect. 1993;6(2):119-26.
131. Bao LH, Zhang YN, Zhang JN, Gu L, Yang HM, Huang YY, et al. Urate inhibits microglia activation to protect neurons in an LPS-induced model of Parkinson's disease. J Neuroinflamm. 2018;15.
132. Bourre JM, Gozlandevillierre N, Morand O, Baumann N. Importance of Exogenous Saturated Fatty-Acids during Brain-Development and Myelination in Mice. Ann Biol Anim Bioch. 1979;19(Nb1):173-80.
133. Rodriguez-Navas C, Morselli E, Clegg DJ. Sexually dimorphic brain fatty acid composition in low and high fat diet-fed mice. Mol Metab. 2016;5(8):680-9.
134. Patil S, Chan C. Palmitic and stearic fatty acids induce Alzheimer-like hyperphosphorylation of tau in primary rat cortical neurons. Neurosci Lett. 2005;384(3):288-93.
135. Amtul Z, Uhrig M, Rozmahel RF, Beyreuther K. Structural Insight into the Differential Effects of Omega-3 and Omega-6 Fatty Acids on the Production of A beta Peptides and Amyloid Plaques. Journal of Biological Chemistry. 2011;286(8):6100-7.
136. Fraser T, Tayler H, Love S. Fatty Acid Composition of Frontal, Temporal and Parietal Neocortex in the Normal Human Brain and in Alzheimer's Disease. Neurochem Res. 2010;35(3):503-13.
137. Bourre JM, Gozlan-Devillierre N, Pollet S, Maurin Y, Baumann N. In vivo incorporation of exogenous stearic acid in synaptosomes: high occurrence of non-esterified fatty acids. Neurosci Lett. 1977;4(6):309-13.
138. Gupta S, Knight AG, Gupta S, Keller JN, Bruce-Keller AJ. Saturated long-chain fatty acids activate inflammatory signaling in astrocytes. J Neurochem. 2012;120(6):1060-71.
139. Moazedi AA, Hossienzadeh Z, Chinpardaz R. The effects of coadministration palmitic acid and oleic acid (omega 9) on spatial learning and motor activity in adult male rat. Pak J Biol Sci. 2007;10(20):3650-5.
140. Patil S, Sheng L, Masserang A, Chan C. Palmitic acid-treated astrocytes induce BACE1 upregulation and accumulation of C-terminal fragment of APP in primary cortical neurons. Neurosci Lett. 2006;406(1-2):55-9.
141. Yanguas-Casas N, Crespo-Castrillo A, de Ceballos ML, Chowen JA, Azcoitia I, Arevalo MA, et al. Sex differences in the phagocytic and migratory activity of microglia and their impairment by palmitic acid. Glia. 2018;66(3):522-37.
142. Odutuga AA. Long-term deficiency of essential fatty acids in rats and its effect on brain recovery. Clin Exp Pharmacol Physiol. 1979;6(4):361-6.
143. Harauma A, Yasuda H, Hatanaka E, Nakamura MT, Salem N, Jr., Moriguchi T. The essentiality of arachidonic acid in addition to docosahexaenoic acid for brain growth and function. Prostaglandins Leukot Essent Fatty Acids. 2017;116:9-18.
144. Snowden SG, Ebshiana AA, Hye A, An Y, Pletnikova O, O'Brien R, et al. Association between fatty acid metabolism in the brain and Alzheimer disease neuropathology and cognitive performance: A nontargeted metabolomic study. Plos Med. 2017;14(3).
145. Hussain G, Schmitt F, Loeffler JP, de Aguilar JLG. Fatting the brain: a brief of recent research. Frontiers in Cellular Neuroscience. 2013;7.
146. Scherma M, Masia P, Satta V, Fratta W, Fadda P, Tanda G. Brain activity of anandamide: a rewarding bliss? Acta Pharmacol Sin. 2019;40(3):309-23.
147. Boutaud O, Montine TJ, Chang L, Klein WL, Oates JA. PGH2-derived levuglandin adducts increase the neurotoxicity of amyloid beta1-42. J Neurochem. 2006;96(4):917-23.
148. Saper CB, Romanovsky AA, Scammell TE. Neural circuitry engaged by prostaglandins during the sickness syndrome. Nat Neurosci. 2012;15(8):1088-95.
149. Martano G, Murru L, Moretto E, Gerosa L, Garrone G, Krogh V, et al. Biosynthesis of glycerol phosphate is associated with long-term potentiation in hippocampal neurons. Metabolomics. 2016;12(8).

150. Owen L, Sunram-Lea SI. Metabolic Agents that Enhance ATP can Improve Cognitive Functioning: A Review of the Evidence for Glucose, Oxygen, Pyruvate, Creatine, and L-Carnitine. Nutrients. 2011;3(8):735-55.
151. Zhang C, Rissman RA, Feng J. Characterization of ATP alternations in an Alzheimer's disease transgenic mouse model. J Alzheimers Dis. 2015;44(2):375-8.
152. Inoue K. Microglial activation by purines and pyrimidines. Glia. 2002;40(2):156-63.
153. Davalos D, Grutzendler J, Yang G, Kim JV, Zuo Y, Jung S, et al. ATP mediates rapid microglial response to local brain injury in vivo. Nat Neurosci. 2005;8(6):752-8.
154. Izumi Y, Benz AM, Katsuki H, Matsukawa M, Clifford DB, Zorumski CF. Effects of fructose-1,6-bisphosphate on morphological and functional neuronal integrity in rat hippocampal slices during energy deprivation. Neuroscience. 2003;116(2):465-75.
155. Veras FP, Peres RS, Saraiva AL, Pinto LG, Louzada-Junior P, Cunha TM, et al. Fructose 1,6-bisphosphate, a high-energy intermediate of glycolysis, attenuates experimental arthritis by activating anti-inflammatory adenosinergic pathway. Sci Rep. 2015;5:15171.
156. Lenglet A, Liabeuf S, Bodeau S, Louvet L, Mary A, Boullier A, et al. N-methyl-2-pyridone-5-carboxamide (2PY)-Major Metabolite of Nicotinamide: An Update on an Old Uremic Toxin. Toxins. 2016;8(11).
157. Sternak M, Khomich TI, Jakubowski A, Szafarz M, Szczepanski W, Bialas M, et al. Nicotinamide N-methyltransferase (NNMT) and 1-methylnicotinamide (MNA) in experimental hepatitis induced by concanavalin A in the mouse. Pharmacol Rep. 2010;62(3):483-93.

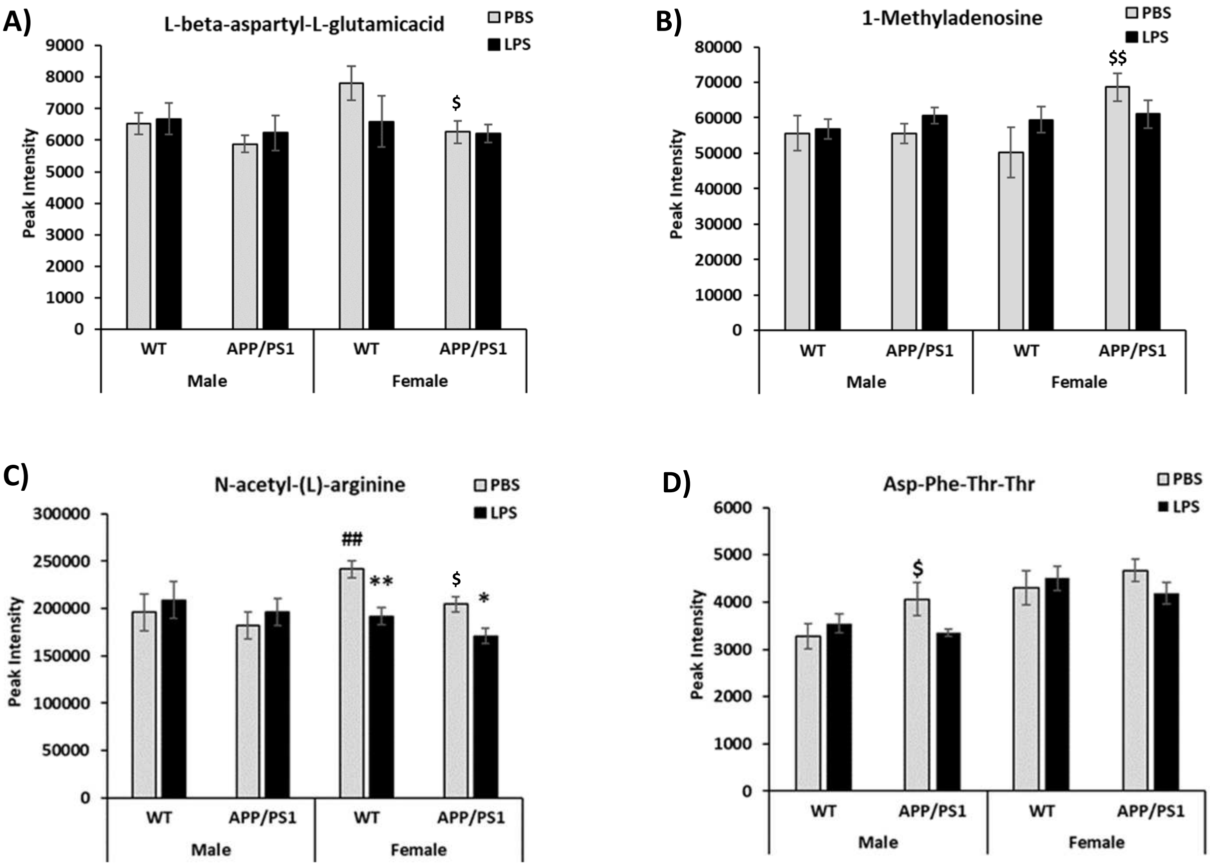

Supplementary. Fig. 2. Metabolites affected in APP/PS1 mice in the absence of immune stimulation. Levels of L-beta-aspartyl-L-glutamic acid, were particularly reduced in female APP/PS1 mice (A). B) 1-Methyladenosine, was more abundant in the hippocampus of female APP/PS1 mice compared to their WT female littermates (B). Significant Genotype X Treatment interactions were also found for N-acetyl-(L)-arginine ($F_{(1,34)}$=12.07, p=0.001), whose levels were significantly lower in PBS-treated APP/PS1 females compared to PBS-treated WT females (C), and for the hydrophobic tetrapeptide Asp-Phe-Thr-Thr ($F_{(1,34)}$=5.40, p=0.03), whose levels were significantly increased in PBS-treated APP/PS1 males compared to their PBS-treated counterparts (D). Data are expressed as Means $\pm$ SEM. Pairwise comparisons following 3-way ANOVAs: $^{\$}$, p<0.05; $^{\$\$}$, p<0.01 compared to WT PBS-treated (same sex); $^{\#\#}$, p<0.01 compared to PBS-treated males (same genotype); *, p<0.05; **, p<0.01 compared to PBS-treated mice of same sex and genotype.

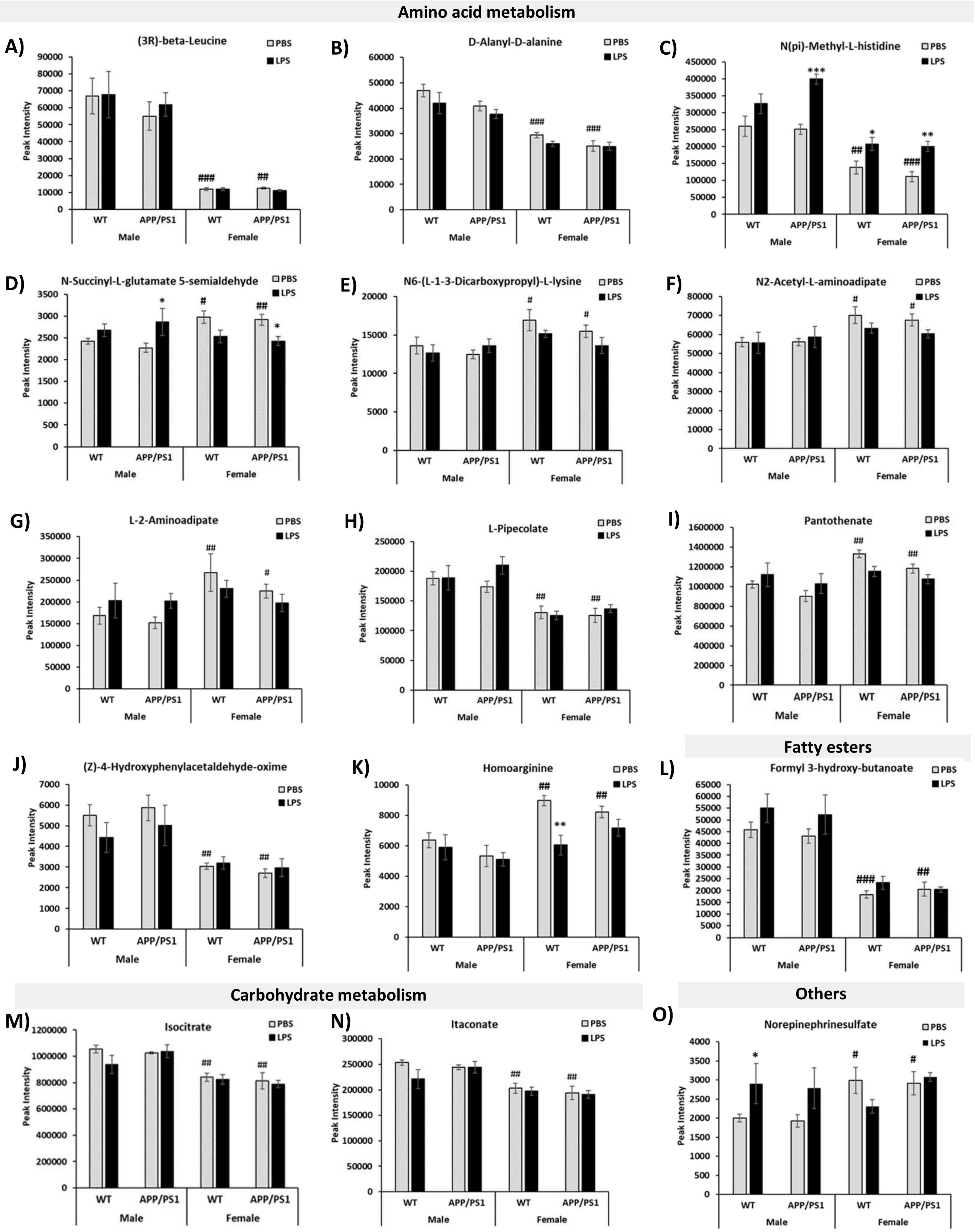

Supplementary Figure 3. Metabolites with sex differences in the hippocampus. Data are expressed as Means ± SEM. Post-hoc tests following 3-way ANOVAs: $^{\#}$, p<0.05, $^{\#\#}$, p<0.01 compared to PBS-treated males (same genotype); **, p<0.01; ***, p<0.0001 compared to PBS-treated mice of same sex and genotype.

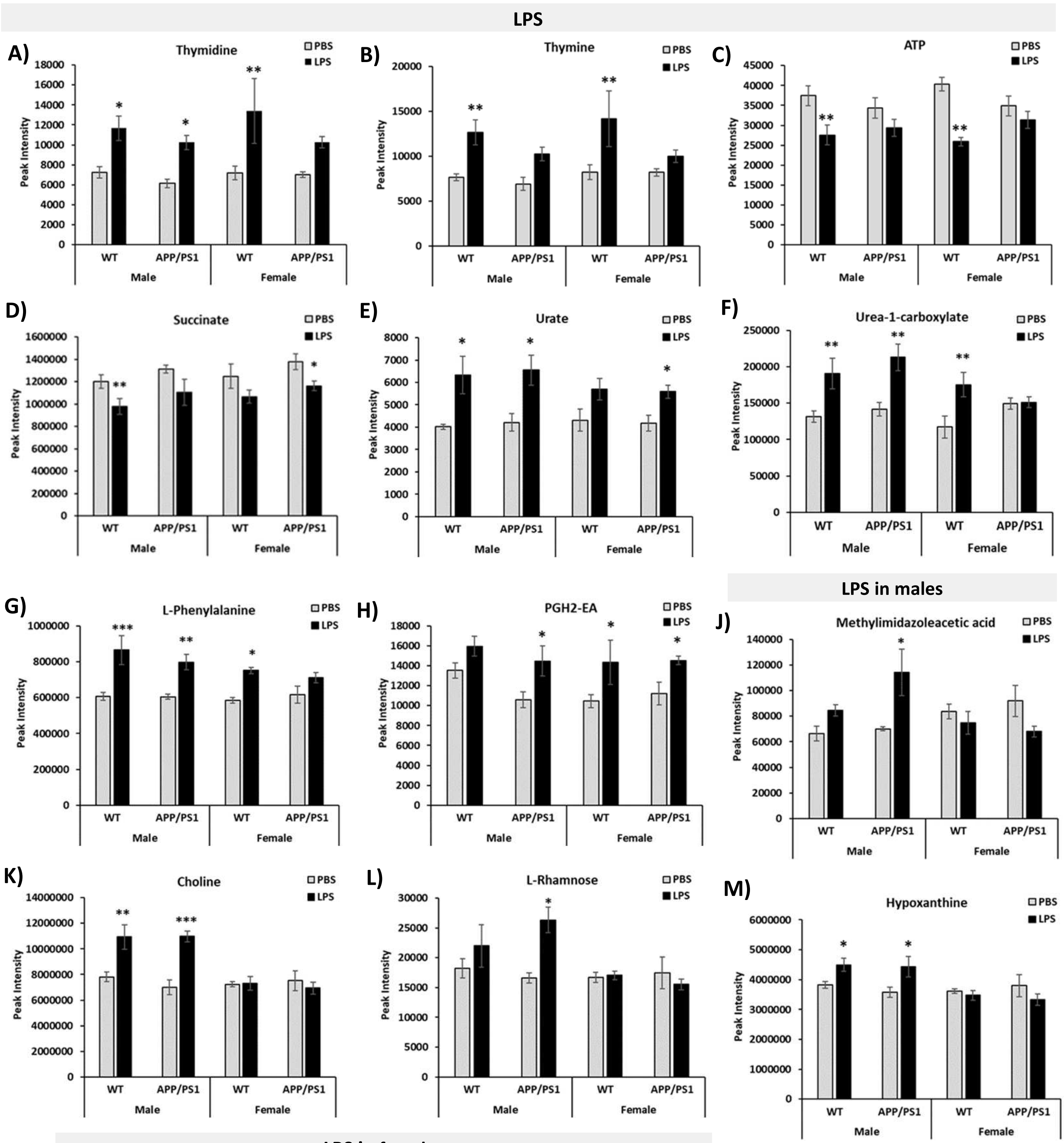

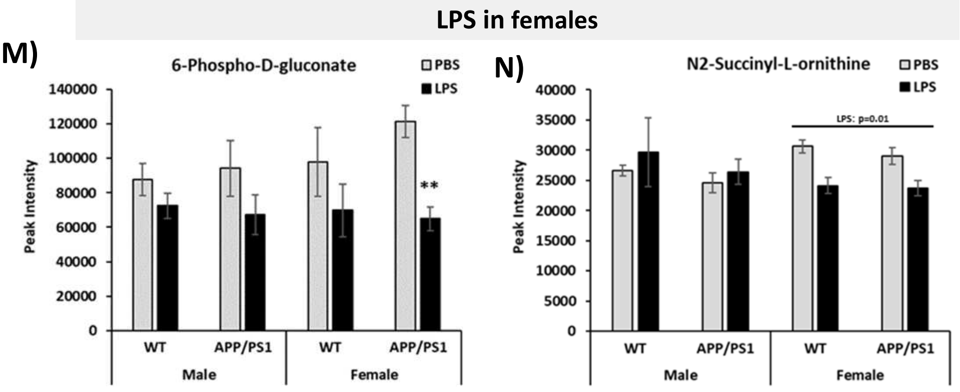

Supplementary Figure 4. Metabolites responding to LPS in the hippocampus. Data are expressed as Means ± SEM. Post-hoc tests following 3-way ANOVAs: *, p<0.05; **, p<0.01; ***, p<0.0001 compared to PBS-treated mice of same sex and genotype.

Supplementary table 3. Results of the two-way repeated measure ANOVAs on glial measures.

	% area stained by Iba1	Number of cells/mm²	Microglial soma area	% area stained by GFAP
Whole hippocampus				
**Genotype**	$F_{(1,35)}$=0.96; p=0.33	$F_{(1,35)}$=0.43; p=0.51	$F_{(1,35)}$=0.00; p=0.95	$F_{(1,33)}$=3.81; p=0.059
**Sex**	$F_{(1,35)}$=0.80; p=0.38	$F_{(1,35)}$=3.28; p=0.08	$F_{(1,35)}$=2.16; p=0.15	$F_{(1,33)}$=1.02; p=0.32
**Treatment**	$F_{(1,35)}$=2.26; p=0.14	$F_{(1,35)}$=1.39; p=0.24	$F_{(1,35)}$=0.20; p=0.66	$F_{(1,33)}$=0.16; p=0.69
**Genotype X Sex**	**$F_{(1,35)}$=4.14; p=0.049**	$F_{(1,35)}$=3.01; p=0.09	$F_{(1,35)}$=0.20; p=0.75	$F_{(1,33)}$=0.87; p=0.36
**Genotype X Treatment**	$F_{(1,35)}$=0.65; p=0.42	$F_{(1,35)}$=0.22; p=0.64	$F_{(1,35)}$=0.10; p=0.70	$F_{(1,33)}$=0.13; p=0.72
**Sex X Treatment**	$F_{(1,35)}$=0.71; p=0.40	$F_{(1,35)}$=0.23; p=0.64	$F_{(1,35)}$=0.75; p=0.39	$F_{(1,33)}$=0.66; p=0.42
**Genotype X Sex X Treatment**	$F_{(1,35)}$=0.98; p=0.33	$F_{(1,35)}$=1.75; p=0.19	$F_{(1,35)}$=0.42; p=0.52	$F_{(1,33)}$=0.06; p=0.80
CA1				
**Genotype**	$F_{(1,35)}$=1.08; p=0.31	$F_{(1,35)}$=0.07; p=0.79	$F_{(1,35)}$=0.02; p=0.90	$F_{(1,33)}$=4.13; p=0.0503
**Sex**	$F_{(1,35)}$=1.45; p=0.24	$F_{(1,35)}$=3.21; p=0.08	$F_{(1,35)}$=1.60; p=0.21	$F_{(1,33)}$=1.03; p=0.32
**Treatment**	$F_{(1,35)}$=3.30; p=0.08	$F_{(1,35)}$=0.19; p=0.66	$F_{(1,35)}$=0.15; p=0.70	$F_{(1,33)}$=0.16; p=0.69
**Genotype X Sex**	$F_{(1,35)}$=3.56; p=0.07	$F_{(1,35)}$=1.95; p=0.17	$F_{(1,35)}$=0.06; p=0.80	$F_{(1,33)}$=0.43; p=0.52
**Genotype X Treatment**	$F_{(1,35)}$=0.51; p=0.48	$F_{(1,35)}$=0.13; p=0.72	$F_{(1,35)}$=0.03; p=0.86	$F_{(1,33)}$=0.03; p=0.85
**Sex X Treatment**	$F_{(1,35)}$=1.15; p=0.29	$F_{(1,35)}$=0.50; p=0.48	$F_{(1,35)}$=0.42; p=0.52	$F_{(1,33)}$=0.20; p=0.66
**Genotype X Sex X Treatment**	$F_{(1,35)}$=0.89; p=0.35	$F_{(1,35)}$=1.76; p=0.19	$F_{(1,35)}$=0.52; p=0.48	$F_{(1,33)}$=0.68; p=0.42
CA2				
**Genotype**	$F_{(1,35)}$=0.82; p=0.37	$F_{(1,34)}$=0.45; p=0.51	$F_{(1,34)}$=0.34; p=0.56	$F_{(1,32)}$=0.18; p=0.67
**Sex**	$F_{(1,35)}$=0.73; p=0.40	$F_{(1,34)}$=0.66; p=0.42	$F_{(1,34)}$=2.01; p=0.16	$F_{(1,32)}$=0.13; p=0.72
**Treatment**	$F_{(1,35)}$=1.29; p=0.26	$F_{(1,34)}$=1.00; p=0.32	$F_{(1,34)}$=0.22; p=0.64	$F_{(1,32)}$=1.93; p=0.17
**Genotype X Sex**	**$F_{(1,35)}$=4.24; p=0.047**	$F_{(1,34)}$=2.26; p=0.14	$F_{(1,34)}$=0.04; p=0.84	$F_{(1,32)}$=0.03; p=0.87
**Genotype X Treatment**	$F_{(1,35)}$=0.02; p=0.90	$F_{(1,34)}$=0.10; p=0.75	$F_{(1,34)}$=0.09; p=0.77	$F_{(1,32)}$=0.00; p=0.99
**Sex X Treatment**	$F_{(1,35)}$=0.11; p=0.74	$F_{(1,34)}$=0.61; p=0.44	$F_{(1,34)}$=0.93; p=0.34	$F_{(1,32)}$=1.54; p=0.22
**Genotype X Sex X Treatment**	$F_{(1,35)}$=0.29; p=0.59	$F_{(1,34)}$=0.03; p=0.85	$F_{(1,34)}$=0.95; p=0.33	$F_{(1,32)}$=0.13; p=0.71
CA3				
**Genotype**	$F_{(1,36)}$=2.50; p=0.12	$F_{(1,36)}$=1.79; p=0.19	$F_{(1,36)}$=0.00; p=0.99	$F_{(1,33)}$=1.21; p=0.28
**Sex**	$F_{(1,36)}$=0.97; p=0.33	$F_{(1,36)}$=1.55; p=0.22	$F_{(1,36)}$=3.62; p=0.06	$F_{(1,33)}$=0.41; p=0.53
**Treatment**	$F_{(1,36)}$=1.77; p=0.19	$F_{(1,36)}$=3.08; p=0.09	$F_{(1,36)}$=0.01; p=0.93	$F_{(1,33)}$=0.24; p=0.63
**Genotype X Sex**	**$F_{(1,36)}$=7.37; p=0.01**	$F_{(1,36)}$=3.04; p=0.09	$F_{(1,36)}$=0.00; p=0.96	$F_{(1,33)}$=0.76; p=0.39
**Genotype X Treatment**	$F_{(1,36)}$=0.56; p=0.46	$F_{(1,36)}$=0.59; p=0.45	$F_{(1,36)}$=0.16; p=0.69	$F_{(1,33)}$=0.09; p=0.76
**Sex X Treatment**	$F_{(1,36)}$=0.98; p=0.33	$F_{(1,36)}$=0.03; p=0.85	$F_{(1,36)}$=0.68; p=0.41	$F_{(1,33)}$=1.15; p=0.29
**Genotype X Sex X Treatment**	$F_{(1,36)}$=0.47; p=0.49	$F_{(1,36)}$=0.05; p=0.82	$F_{(1,36)}$=1.08; p=0.30	$F_{(1,33)}$=0.01; p=0.93
Dentate Gyrus				
**Genotype**	$F_{(1,36)}$=1.33; p=0.26	$F_{(1,36)}$=0.17; p=0.68	$F_{(1,36)}$=0.04; p=0.83	$F_{(1,33)}$=3.43; p=0.07
**Sex**	$F_{(1,36)}$=0.82; p=0.37	$F_{(1,36)}$=3.69; p=0.06	$F_{(1,36)}$=2.82; p=0.10	$F_{(1,33)}$=0.15; p=0.70
**Treatment**	$F_{(1,36)}$=3.15; p=0.08	$F_{(1,36)}$=0.00; p=0.99	$F_{(1,36)}$=0.75; p=0.39	$F_{(1,33)}$=0.09; p=0.77
**Genotype X Sex**	$F_{(1,36)}$=2.81; p=0.10	**$F_{(1,36)}$=5.02; p=0.03**	$F_{(1,36)}$=0.01; p=0.92	$F_{(1,33)}$=0.68; p=0.41
**Genotype X Treatment**	$F_{(1,36)}$=0.14; p=0.71	$F_{(1,36)}$=0.45; p=0.50	$F_{(1,36)}$=0.50; p=0.48	$F_{(1,33)}$=0.94; p=0.34
**Sex X Treatment**	$F_{(1,36)}$=0.51; p=0.48	$F_{(1,36)}$=0.11; p=0.74	$F_{(1,36)}$=0.96; p=0.34	$F_{(1,33)}$=1.75; p=0.19
**Genotype X Sex X Treatment**	$F_{(1,36)}$=0.83; p=0.36	$F_{(1,36)}$=1.17; p=0.29	$F_{(1,36)}$=0.02; p=0.90	$F_{(1,33)}$=0.10; p=0.76

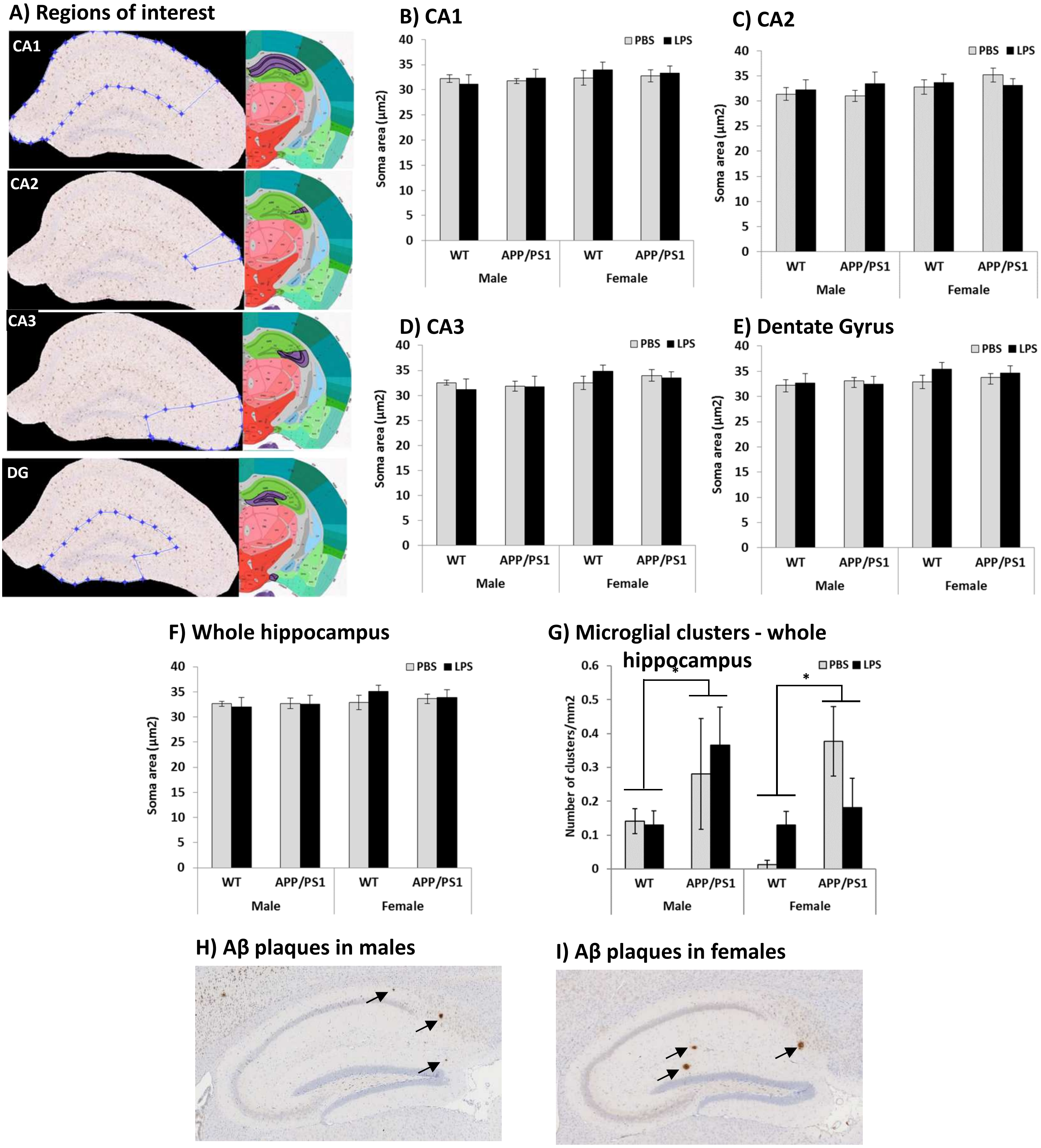

Supplementary Figure 5. Microglial soma size a morphometric marker of microglial activation was measured using Iba1 immunostaining at 4 hours after the PBS or LPS challenge. Illustration of the regions of interests used for microglia and astrocytes segmentation, delineated using a custom made Matlab tool. No differences were found in the whole hippocampus (B), CA1(C), CA2 (D), CA3 (E) or Dentate Gyrus (F) subfields. The number of microglial clusters was elevated in APP/PS1 mice ($F_{(1,35)}$=10.05, p=0.003; G), which displayed relatively few plaques (white arrows) at 4.5 months of age (H, I). *, p<0.05. Data are expressed as Means $\pm$ SEM.

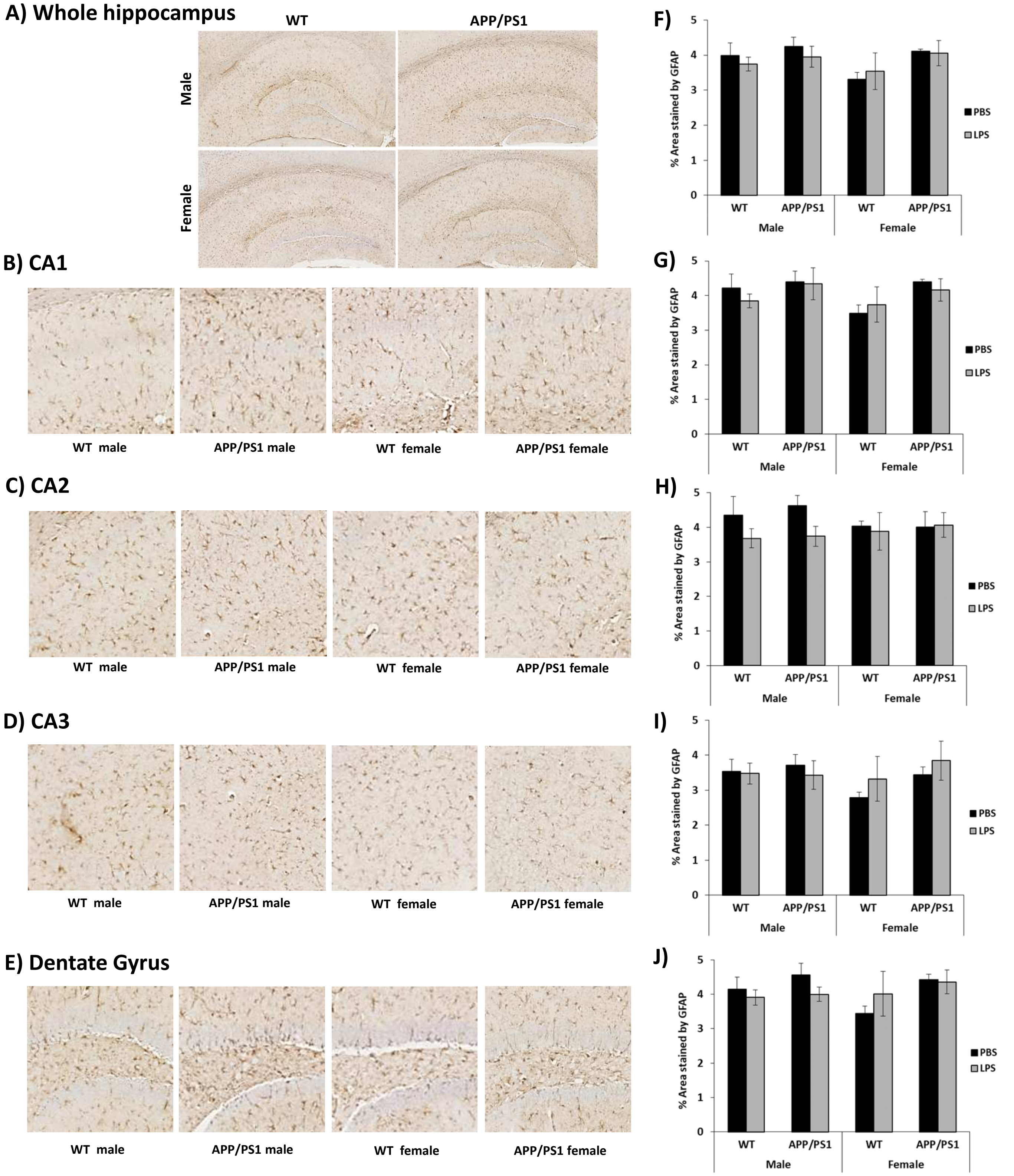

Supplementary Figure 6. The area occupied by astrocytes was quantified using GFAP immunostaining 4 hours after the PBS or LPS challenge. Representative image of GFAP positive astrocytes in the whole hippocampus (A), CA1(B), CA2 (C), CA3 (D) or Dentate Gyrus (E) subfields. No differences were found between any of the experimental conditions in these regions of interests: whole hippocampus (F), CA1(G), CA2 (H), CA3 (I) or Dentate Gyrus (J). Data are expressed as Means $\pm$ SEM.
